# WSGC@FA@PEG/PEI‐SPIONs Mitigate Chemoresistance in Gastric Adenocarcinoma by Modulating the Notch Signaling Pathway and Mitophagy

**DOI:** 10.1002/advs.202415840

**Published:** 2025-08-19

**Authors:** Dongjian Song, Qiuliang Liu, Zechen Yan, Qi Wang, Meng Su, Hui Zhang, Longyan Shi, Yingzhong Fan, Qian Zhang, Heying Yang, Da Zhang

**Affiliations:** ^1^ Department of Pediatric Surgery the First Affiliated Hospital of Zhengzhou University Zhengzhou Henan 450052 P. R. China; ^2^ Institute of Molecular Cancer Surgery Zhengzhou University Henan Province Engineering Research Center Zhengzhou Henan 450052 P. R. China; ^3^ Department of Urology the First Affiliated Hospital of Zhengzhou University Zhengzhou Henan 450052 P. R. China

**Keywords:** chemotherapy resistance, gastric adenocarcinoma, mitophagy, nanoparticles, Notch signaling pathway, WSGC Peptides

## Abstract

This study investigates the molecular mechanisms by which superparamagnetic iron oxide nanoparticles (SPIONs) loaded with the WSGC peptide (WSGC@FA@PEG/PEI‐SPIONs)—a 40‐amino acid polypeptide derived from apoC‐III—modulate chemotherapy resistance in gastric adenocarcinoma (GA). Emphasis is placed on their role in regulating mitophagy and mitochondrial homeostasis via the Notch signaling pathway. The physicochemical properties of WSGC@FA@PEG/PEI‐SPIONs are thoroughly characterized, demonstrating favorable biocompatibility, stable size distribution, and efficient peptide loading. In vitro experiments show that these nanoparticles significantly inhibit GA cell proliferation, migration, and invasion by downregulating mitophagy‐associated proteins (LC3, PINK1, and Parkin), primarily through modulation of the Notch pathway. In vivo studies, using a GA nude mouse model, confirm the therapeutic potential of WSGC@FA@PEG/PEI‐SPIONs, revealing marked tumor growth inhibition and increased apoptotic activity. Collectively, the findings highlight the WSGC peptide as a promising therapeutic agent for overcoming chemotherapy resistance in GA by targeting the Notch signaling pathway and suppressing mitophagy, thereby presenting a novel strategy for polypeptide‐based cancer therapy.

## Graphical abstract

WSGC peptide reverses drug resistance in GA by modulating the Notch signaling pathway and mitophagy at the molecular level.

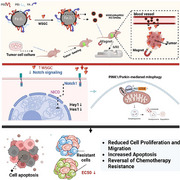

## 1 Introduction

Gastric adenocarcinoma (GA) is one of the most prevalent malignant tumors worldwide, ranking among the leading causes of cancer incidence and mortality in numerous countries.^[^ ^1^, ^2^ ^]^ Epidemiological data show that GA accounts for millions of new cases and a substantial proportion of cancer‐related deaths annually.^[^ ^3^, ^4^, ^5^ ^]^ Although progress in comprehensive treatment strategies—including surgery, radiotherapy, and chemotherapy—the overall prognosis for GA patients remains poor, especially in advanced stages.^[^ ^6^, ^7^, ^8^ ^]^ Chemotherapy remains a cornerstone in GA treatment, capable of suppressing tumor cell proliferation to some extent. However, the widespread occurrence of drug resistance continues to limit its long‐term effectiveness.^[^ ^9^, ^10^ ^]^ This resistance not only complicates therapeutic management, but also significantly compromises patient survival.^[^ ^11^, ^12^, ^13^ ^]^ Therefore, there is an urgent need to elucidate the underlying mechanisms of chemotherapy resistance in GA in order to develop more effective therapeutic strategies.

Mitophagy—a selective form of autophagy that degrades damaged or dysfunctional mitochondria—plays a critical role in maintaining cellular homeostasis.^[^ ^14^ ^]^ Under physiological conditions, mitophagy supports energy metabolism and helps cells respond to various stressors.^[^ ^15^ ^]^ However, in cancer cells, dysregulated mitophagy is closely linked to tumor development, progression, and resistance to therapy.^[^ ^16^ ^]^ Existing studies have shown that mitophagy is frequently activated in tumor cells, enabling them to evade the cytotoxic effects of chemotherapeutic agents.^[^ ^17^, ^18^ ^]^ This evidence suggests that mitophagy contributes to drug resistance, highlighting it as a promising target for overcoming chemotherapy failure.^[^ ^19^, ^20^, ^21^ ^]^

The Notch signaling pathway is a highly conserved cellular communication mechanism that plays a fundamental role in cell differentiation, proliferation, and apoptosis during embryonic development and adult tissue homeostasis.^[^ ^22^, ^23^, ^24^ ^]^ Aberrant activation of this pathway has been observed in various malignancies, particularly in GA.^[^ ^25^, ^26^, ^27^ ^]^ In GA, dysregulation of the Notch signaling pathway not only promotes tumor cell proliferation and invasion, but also modulates cellular metabolism and survival by influencing autophagy processes.^[^ ^28^ ^]^ Increasing evidence suggests a strong association between Notch signaling and the expression of mitophagy‐related proteins, identifying this pathway as a potential therapeutic target.^[^ ^29^ ^]^ Inhibition of Notch signaling may thus represent a viable strategy to regulate mitophagy and overcome chemotherapy resistance in tumor cells.^[^ ^30^, ^31^ ^]^

Superparamagnetic iron oxide nanoparticles (SPIONs) have emerged as promising tools for integrated cancer diagnosis and therapy due to their unique physicochemical properties and excellent biocompatibility.^[^ ^32^, ^33^, ^34^ ^]^ SPIONs can act as drug delivery vehicles that improve drug accumulation at tumor sites, while also enabling early detection and precise tumor localization through magnetic resonance imaging (MRI).^[^ ^35^, ^36^, ^37^ ^]^ In recent years, polypeptide‐modified SPIONs have attracted growing interest in targeted cancer therapy.^[^ ^38^, ^39^ ^]^ Among these, the WSGC peptide—a polypeptide with high specificity for tumor cells—has demonstrated strong targeting capabilities. When modified with FA@PEG/PEI, the resulting WSGC@SPIONs nanoparticles exhibit excellent targeting precision, enhanced stability, and improved drug‐loading and release efficiency. These features position polypeptide‐functionalized nanoparticles as a promising new strategy in cancer treatment.

This study aims to explore the molecular mechanisms by which SPIONs loaded with the WSGC peptide (WSGC@FA@PEG/PEI‐SPIONs) modulate chemotherapy resistance in GA, with a particular focus on their regulation of mitophagy and mitochondrial homeostasis via the Notch signaling pathway. The physicochemical characteristics of WSGC@FA@PEG/PEI‐SPIONs were systematically evaluated to ensure their stability and functional performance in biological environments. In vitro assays were conducted to assess their effects on GA cell proliferation, migration, invasion, and mitophagy. In addition, transcriptome sequencing and in vivo animal models were employed to elucidate the underlying molecular mechanisms and evaluate anti‐tumor efficacy. The findings demonstrate that WSGC@FA@PEG/PEI‐SPIONs significantly suppress GA cell proliferation, migration, and invasion, and effectively reverse chemotherapy resistance by downregulating the expression of mitophagy‐associated proteins (LC3, PINK1, and Parkin) through inhibition of the Notch signaling pathway. This research presents a novel approach and potential therapeutic target for peptide‐based cancer treatments, offering significant scientific and clinical value. These results may provide renewed hope for improving outcomes in patients with GA.

## 2 Results

### 2.1 Characterization of WSGC@FA@PEG/PEI‐SPIONs

SPIONs, when modified with suitable polymers, serve as efficient drug carriers and can facilitate targeted chemotherapy by leveraging the enhanced permeability and retention (EPR) effect.^[^ ^49^, ^50^ ^]^

In this study, we focused on the WSGC peptide—a non‐inflammatory serum biomarker with a specific mass‐to‐charge ratio (m/z) identified in GA patients at various clinical stages, including preoperative, postoperative, recurrent, and inflammatory conditions such as SIRS. Using proteomic mass spectrometry, HPLC, MALDI‐TOF‐MS, and MALDI‐TOF/TOF‐MS, WSGC was confirmed to be a peptide fragment derived from apoC‐III.^[^ ^51^ ^]^

**Figure 1:**
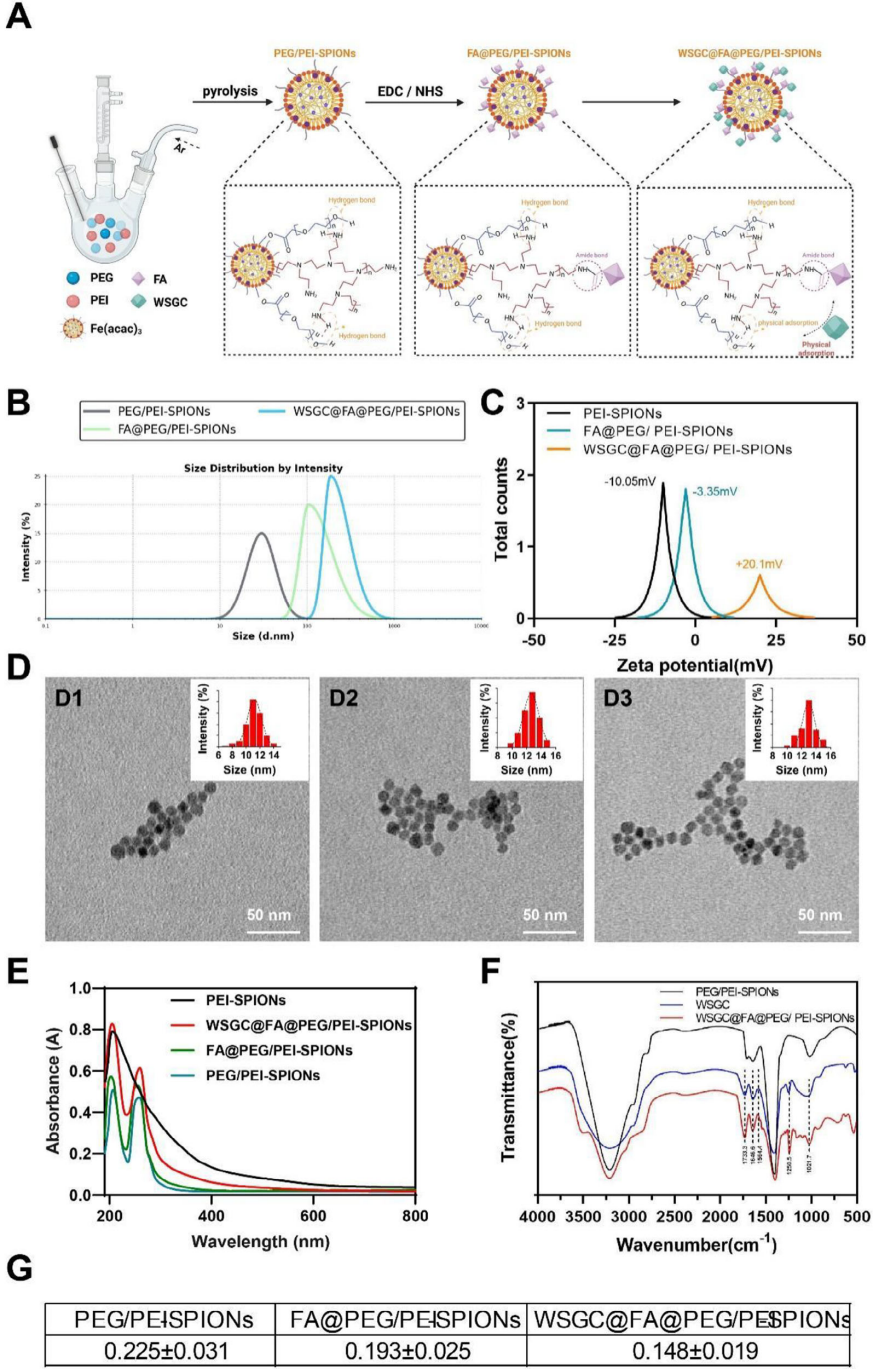
Characteristics of WSGC@FA@PEG/PEI‐SPIONs. Note: A) Schematic representation of the synthesis of WSGC@FA@PEG/PEI‐SPIONs; B) Particle size, C) zeta potential, TEM images, and size distribution (D) of D1) PEG/PEI‐SPIONs, D2) FA@PEG/PEI‐SPIONs, and D3) WSGC@FA@PEG/PEI‐SPIONs; E) PEG/PEI‐SPIONs, FA@PEG/PEI‐SPIONs, UV and F) FTIR spectra of WSGC@FA@PEG/PEI‐SPIONs; G) PDI of Nanoparticles.

Building on previous work,^[^ ^42^ ^]^ we developed a nanoplatform based on PEG/PEI‐coated SPIONs, capable of loading WSGC peptide via physical adsorption and releasing it in a pH‐sensitive manner. The synthesis scheme for WSGC@FA@PEG/PEI‐SPIONs is illustrated in **Figure** **1**A. DLS analysis revealed that the hydrodynamic diameters of PEG/PEI‐SPIONs, FA@PEG/PEI‐SPIONs, and WSGC@FA@PEG/PEI‐SPIONs in PBS were ≈29.2 nm, 100.9 nm, and 183.1 nm, respectively, with relatively narrow size distributions (Figure 1B).

Zeta potential measurements showed a progressive decrease in surface charge from PEG/PEI‐SPIONs (+20 mV) to FA@PEG/PEI‐SPIONs (−3.3 mV) and WSGC@FA@PEG/PEI‐SPIONs (−10 mV), likely due to the incorporation of negatively charged functional groups from FA and WSGC peptides (Figure 1C). TEM images demonstrated well‐dispersed, spherical nanoparticles with core sizes ranging from 10 to 13 nm (Figure 1D), which falls within the optimal size range for efficient tumor‐targeted drug delivery.^[^ ^52^ ^]^

Ultraviolet (UV) and FTIR spectroscopy were employed to examine molecular interactions.^[^ ^53^ ^]^ The WSGC peptide contains aromatic residues such as tryptophan and tyrosine, yielding characteristic absorption at 280 nm, while FA exhibits a peak at 260 nm (Figure 1E). UV‐Vis‐NIR spectra confirmed the presence of these characteristic peaks across the various nanoparticle formulations (Figure 1F).

FTIR spectra revealed no significant shift in characteristic bands upon WSGC conjugation, indicating that WSGC binding did not alter the core structure of the nanoparticles (Figure 1F). Additionally, PDI values for PEG/PEI‐SPIONs, FA@PEG/PEI‐SPIONs, and WSGC@FA@PEG/PEI‐SPIONs were 0.213, 0.185, and 0.162, respectively (Figure 1G).

Taken together, the experimental results demonstrate that the WSGC@FA@PEG/PEI‐SPIONs nano platform possesses good biocompatibility, stable size distribution, and effective polypeptide loading capabilities.

### 2.2 Stability and In Vitro Drug Release Study of WSGC@FA@PEG/PEI‐SPIONs

To evaluate the in vitro stability of WSGC@FA@PEG/PEI‐SPIONs, nanoparticles were incubated under three conditions: (1) 0.01 mol L⁻^1^ PBS, (2) PBS containing 10% fetal bovine serum (FBS), and (3) complete cell culture medium (with 10% FBS), either at 37 °C for 72 h or at 4 °C for 28 days. At 37 °C, a slight increase in particle size was observed over 72 h, indicating minor nanoparticle aggregation (Figure S1A, Supporting Information). Conversely, particles stored at 4 °C remained relatively stable, with negligible size changes throughout the 28‐day period, suggesting excellent long‐term storage stability (Figure S1C, Supporting Information).

Drug retention studies further demonstrated that WSGC remained stably encapsulated within the nanoparticles. At 37 °C, over 80% of the drug was retained within the nanocarrier for up to 48 h (Figure S1B, Supporting Information). At 4 °C, drug content remained above 90% for the first 7 days and declined modestly to ≈85% thereafter (Figure S1D, Supporting Information). These results suggest high serum and storage stability, likely due to the amphiphilic nature and favorable physicochemical balance of the formulation components.^[^ ^54^ ^]^

The pH‐responsive release profile of WSGC from WSGC@FA@PEG/PEI‐SPIONs was assessed in PBS at physiological (pH 7.4) and acidic conditions (pH 6.0 and 5.0) to simulate the tumor microenvironment. As shown in Figure S1E (Supporting Information), drug release at pH 7.4 was minimal over 72 h, with no significant burst release, indicating stability during circulation and reduced premature drug leakage. In contrast, at pH 5.0, over 82% of the WSGC payload was released within 48 h. This pH‐sensitive release is likely driven by protonation‐induced disruption of the physical adsorption between the coating polymer and the WSGC peptide.

In conclusion, these findings demonstrate that WSGC@FA@PEG/PEI‐SPIONs possess excellent in vitro stability, serum stability, and pH‐sensitive drug release properties, suggesting broad potential applications in targeted cancer chemotherapy and related fields.

### 2.3 WSGC@FA@PEG/PEI‐SPIONs Exhibit Enhanced Cellular Uptake and Potent Cytotoxicity

**Figure 2:**
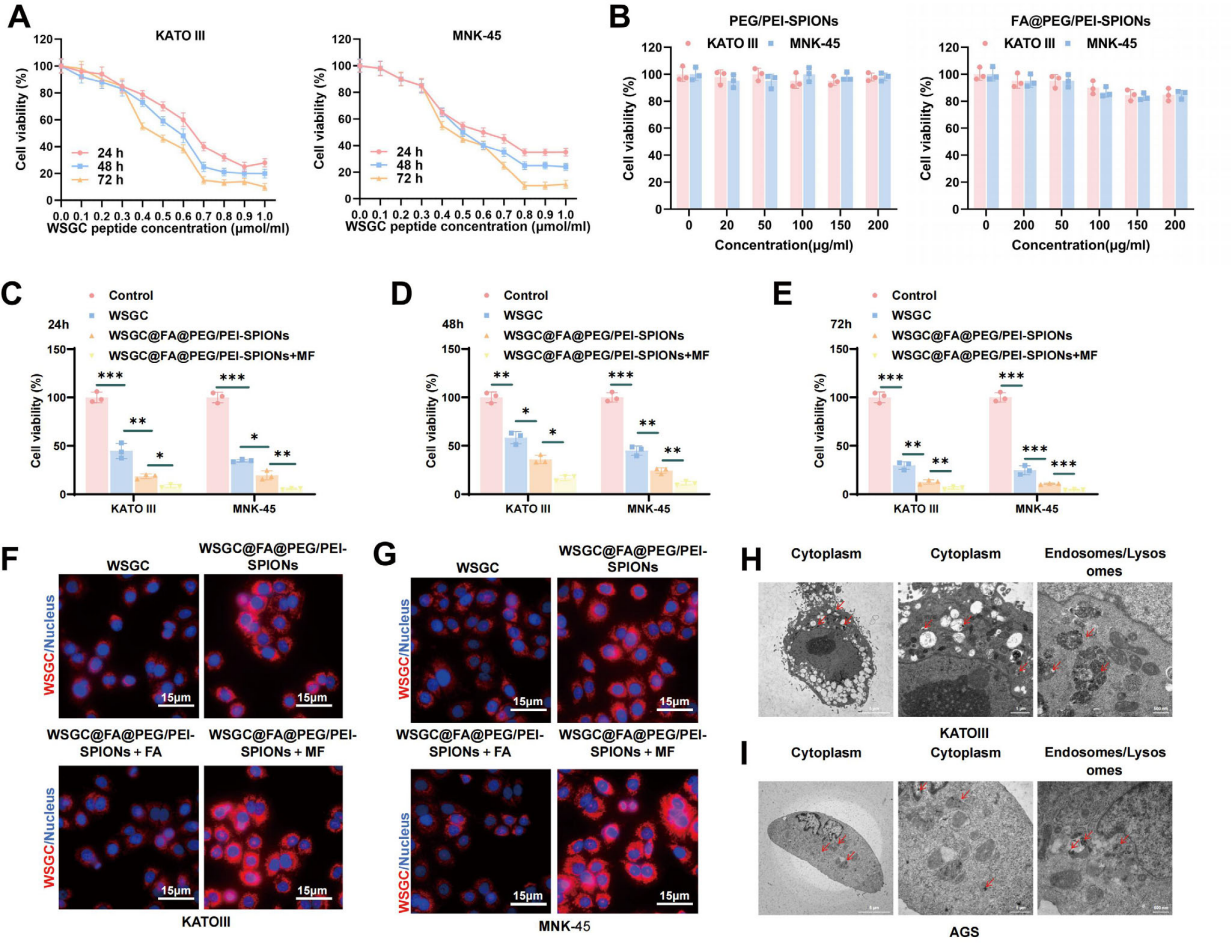
In vitro cytotoxicity and cellular uptake experiments of WSGC@FA@PEG/PEI‐SPIONs. Note: A) Influence of WSGC at gradient concentrations on the viability of human GA cells KATO III (left) and MNK‐45 (right) after treatment for 24, 48, and 72; B) MTT assay to evaluate the cell viability of human GA cells (KATO III and MNK‐45) after incubation with PEG/PEI‐SPIONs (left) and FA@PEG/PEI‐SPIONs (right) at Fe concentrations of 0–100 µg mL⁻^1^ for 24 h; C–E) In vitro cytotoxicity of WSGC@FA@PEG/PEI‐SPIONs (equiv. WSGC concentration of 600 µm) after treatment for C) 24 h, D) 48 h, or E) 72 h on human GA cells (KATO III and MNK‐45); F,G) Fluorescence images showing cellular uptake of free a) WSGC, b) WSGC@FA@PEG/PEI‐SPIONs, c) WSGC@FA@PEG/PEI‐SPIONs + FA, and WSGC@FA@PEG/PEI‐SPIONs incubated with F) KATO III and G) MNK‐45 cells under the influence of d) MF (0.15T) for 1 h, with an equiv. WSGC concentration of 600 µm. The images display WSGC fluorescence in red, DAPI‐stained cell nuclei in blue, and the overlay of the two images (scale bar:15 µm; magnification 100X in bottom right corner); H,I) TEM images of human GA cells H) KATO III and I) MNK‐45 incubated with WSGC@FA@PEG/PEI‐SPIONs, showing SPIONs in the a,b) cytoplasm and in the c) nucleus/lysosomes. Data were presented as mean ± SD (*n* = 3). * Indicates statistically significant differences between groups, with *p < 0.05, **p < 0.01, ***p < 0.001; All cell experiments were performed in triplicate (*n* = 3), and results were expressed as Mean ± SD. One‐way ANOVA was used, while two‐way ANOVA was used for comparing data at different time points.

We first assessed the cytotoxicity of WSGC on human GA cell lines KATO III and MNK‐45 at various time points and concentrations. The half‐maximal inhibitory concentration (IC50) values of WSGC on KATO III cells were determined to be 670 µm (24 h), 610 µm (48 h), and 560 µm (72 h), while the IC50 values for MNK‐45 cells were 630 µm (24 h), 570 µm (48 h), and 510 µm (72 h) (**Figure** **2**A).

To evaluate the biocompatibility of the nanocarriers, we conducted an MTT assay using PEG/PEI‐SPIONs and FA@PEG/PEI‐SPIONs at iron concentrations ranging from 0 to 100 µg mL⁻^1^. Both nanoparticle formulations exhibited minimal cytotoxicity after 24 h of incubation, maintaining cell viability above 80% across all concentrations. Even at 200 µg mL⁻^1^, FA@PEG/PEI‐SPIONs demonstrated inhibition rates below 20% for both KATO III and MNK‐45 cells (Figure 2B), confirming their excellent biocompatibility.

Subsequently, we explored the in vitro cytotoxicity of WSGC@FA@PEG/PEI‐SPIONs (equiv. WSGC concentration of 600 µm) in GA cells. Compared to free WSGC, the nanoformulation displayed significantly higher cytotoxicity across 24, 48, and 72 h (Figure 2C–E). Notably, the application of an external magnetic field (MF) further enhanced cytotoxicity, suggesting that MF facilitates greater nanoparticle accumulation at the cellular interface and promotes internalization (Figure 2C–E).

Fluorescence microscopy revealed that cells treated with WSGC@FA@PEG/PEI‐SPIONs exhibited markedly stronger red fluorescence intensity than those treated with free WSGC, under equiv. peptide concentrations (Figure 2F,G). This indicates a more efficient cellular uptake of the nanoparticle formulation, likely via folate receptor‐mediated endocytosis. In contrast, pre‐treatment with free FA partially inhibited WSGC@FA@PEG/PEI‐SPIONs uptake.

Moreover, after a 2‐h incubation of cells treated with WSGC@FA@PEG/PEI‐SPIONs, the WSGC fluorescence was brighter in cells treated with free WSGC compared to those treated with WSGC@FA@PEG/PEI‐SPIONs+FA. This suggests that the endocytosis of WSGC@FA@PEG/PEI‐SPIONs was partially inhibited by FA occupying the FA receptors (Figure 2F,G). The above results imply that the FA receptors occupied by free FA do not allow the internalization of WSGC@FA@PEG/PEI‐SPIONs, indicating the involvement of FA receptor‐mediated endocytosis in the cellular uptake of WSGC@FA@PEG/PEI‐SPIONs.

Furthermore, in the presence of an MF, the WSGC fluorescence in human GA cells was brighter compared to the situation without MF application (Figure 2F,G). TEM confirmed the intracellular localization of WSGC@FA@PEG/PEI‐SPIONs, with abundant electron‐dense SPION clusters observed within the cytoplasm, endosomes, and lysosomes of treated cells in the absence of MF (Figure 2H,I).

In conclusion, the magnetic nanocomposite WSGC@FA@PEG/PEI‐SPIONs exhibit significant cytotoxic effects on human GA cells through an FA receptor‐mediated endocytosis mechanism. The application of an MF enhances its cytotoxicity, offering new possibilities for cancer cell treatment strategies.

### 2.4 WSGC@FA@PEG/PEI‐SPIONs Effectively Inhibit Proliferation, Migration, and Invasion of GA Cells

**Figure 3:**
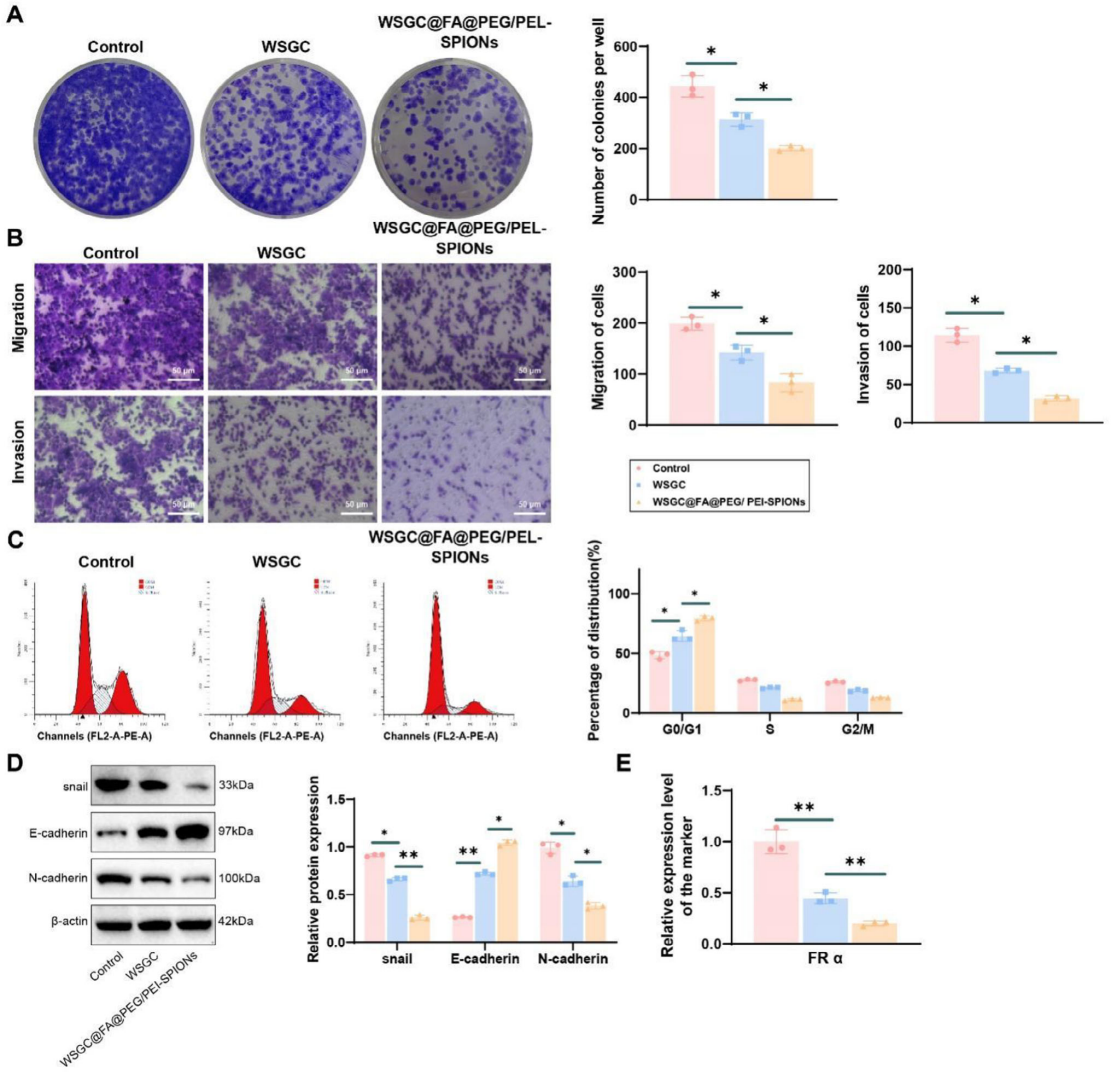
Impact of WSGC@FA@PEG/PEI‐SPIONs on proliferation, migration, and invasion of GA cell line KATO III. Note: A) Clonogenic assay to assess the clonogenic capacity of KATO III cells in each group, Scale bar = 50 µm; B) Transwell assay to evaluate the migratory and invasive capabilities of KATO III cells in each group, Scale bar = 200 µm; C) Flow cytometry analysis of cell cycle distribution in KATO III cells in each group; D) Western blot analysis of the protein expression levels of Snail, E‐cadherin, and N‐cadherin in KATO III cells in each group; E) RT‐qPCR analysis showed a significant reduction in FRα (mRNA) expression in KATO III cells following treatment. *p < 0.05. All cellular experiments were performed in triplicate (*n* = 3). Data were presented as mean ± SD. Comparisons among three or more groups were performed using one‐way ANOVA.

To comprehensively evaluate the functional effects of WSGC@FA@PEG/PEI‐SPIONs on GA cells, a series of assays were conducted, including clonogenic assays, transwell migration and invasion assays, and cell cycle analysis. Clonogenic assay results demonstrated a marked reduction in the colony‐forming ability of GA cells treated with either WSGC alone or WSGC@FA@PEG/PEI‐SPIONs, compared to untreated controls. Notably, WSGC@FA@PEG/PEI‐SPIONs exhibited a more pronounced inhibitory effect, indicating enhanced suppression of GA cell proliferation when WSGC is delivered via the nanoparticle platform (**Figure** **3**A; Figure S2A, Supporting Information).

Transwell assays further revealed that both the WSGC‐treated group and the WSGC@FA@PEG/PEI‐SPIONs‐treated group significantly suppressed the migratory and invasive capacities of GA cells relative to the control group. The inhibition was more robust in the WSGC@FA@PEG/PEI‐SPIONs‐treated group, suggesting that the nanoformulation augments WSGC's anti‐metastatic potential (Figure 3B; Figure S2B, Supporting Information).

Flow cytometric analysis of the cell cycle showed that treatment with either WSGC or WSGC@FA@PEG/PEI‐SPIONs led to a significant accumulation of cells in the G0/G1 phase, accompanied by a corresponding reduction in the S and G2/M phases. These results suggest that both treatments induce cell cycle arrest at the G0/G1 phase, thereby inhibiting tumor cell proliferation, with WSGC@FA@PEG/PEI‐SPIONs eliciting a more substantial effect (Figure 3C; Figure S2C, Supporting Information).

Western blot analysis was employed to assess the expression of key epithelial‐mesenchymal transition (EMT) markers. Compared to the control group, both treatment groups exhibited a notable increase in E‐cadherin expression—a marker of epithelial phenotype—alongside significant reductions in N‐cadherin and Snail levels, which are associated with mesenchymal characteristics and invasiveness.^[^ ^55^ ^]^ Western blot analysis results demonstrate that, compared to the control group, both the WSGC‐treated group and the WSGC@FA@PEG/PEI‐SPIONs‐treated group exhibit a significant increase in the expression levels of E‐cadherin protein, along with a decrease in the expression levels of N‐cadherin and Snail proteins. Moreover, the effect of the WSGC@FA@PEG/PEI‐SPIONs‐treated group is more pronounced than that of the WSGC‐treated group, further corroborating the aforementioned experimental findings (Figure 3D; Figure S2D, Supporting Information). In addition, we analyzed the expression of folate receptor alpha (FRα), a glycosylphosphatidylinositol‐anchored membrane protein involved in folate uptake and known to regulate proliferation and invasiveness in cancer cells.^[^ ^56^ ^]^ Although FRα is overexpressed in over one‐third of gastric cancer patients and rarely expressed in normal tissues, its role in tumor progression remains to be fully elucidated.^[^ ^57^ ^]^ RT‐qPCR analysis revealed that FRα mRNA expression was significantly downregulated following treatment with either WSGC or WSGC@FA@PEG/PEI‐SPIONs, with the greatest reduction observed in the latter group (Figure 3E; Figure S2E, Supporting Information).

In summary, these results demonstrate that WSGC@FA@PEG/PEI‐SPIONs exert a potent anti‐tumor effect by significantly inhibiting GA cell proliferation, migration, and invasion. The enhanced therapeutic efficacy is likely due to improved targeting, intracellular delivery, and retention of the WSGC peptide. These findings support the potential application of WSGC@FA@PEG/PEI‐SPIONs as a promising nanotherapeutic agent in cancer treatment.

### 2.5 Therapeutic Efficacy Study of WSGC@FA@PEG/PEI‐SPIONs in a GA Xenograft Model in Immunocompromised Mice

To evaluate the in vivo therapeutic efficacy of WSGC@FA@PEG/PEI‐SPIONs, subcutaneous xenograft models of GA were established in BALB/c nude mice using KATO III and MNK‐45 cells. Once tumors reached ≈50 mm^3^, mice were randomized into four groups (*n* = 5 per group). The control group received tail vein injections of 0 nmol g⁻^1^ saline, while the experimental groups received WSGC peptide at doses of 20, 40, or 80 nmol g⁻^1^ every 2 days for 21 days. Tumor size was measured with calipers every 3 days, and tumor volume was calculated and plotted over time. Upon treatment completion, mice were euthanized (Figure S3A, Supporting Information), and tumor tissues were collected, photographed, and weighed (Figure S3B, Supporting Information). Compared with the control group, all WSGC‐treated groups exhibited significant tumor growth inhibition, with the 80 nmol g⁻^1^ group demonstrating the most robust anti‐tumor effect (Figure S3C,D, Supporting Information).

**Figure 4:**
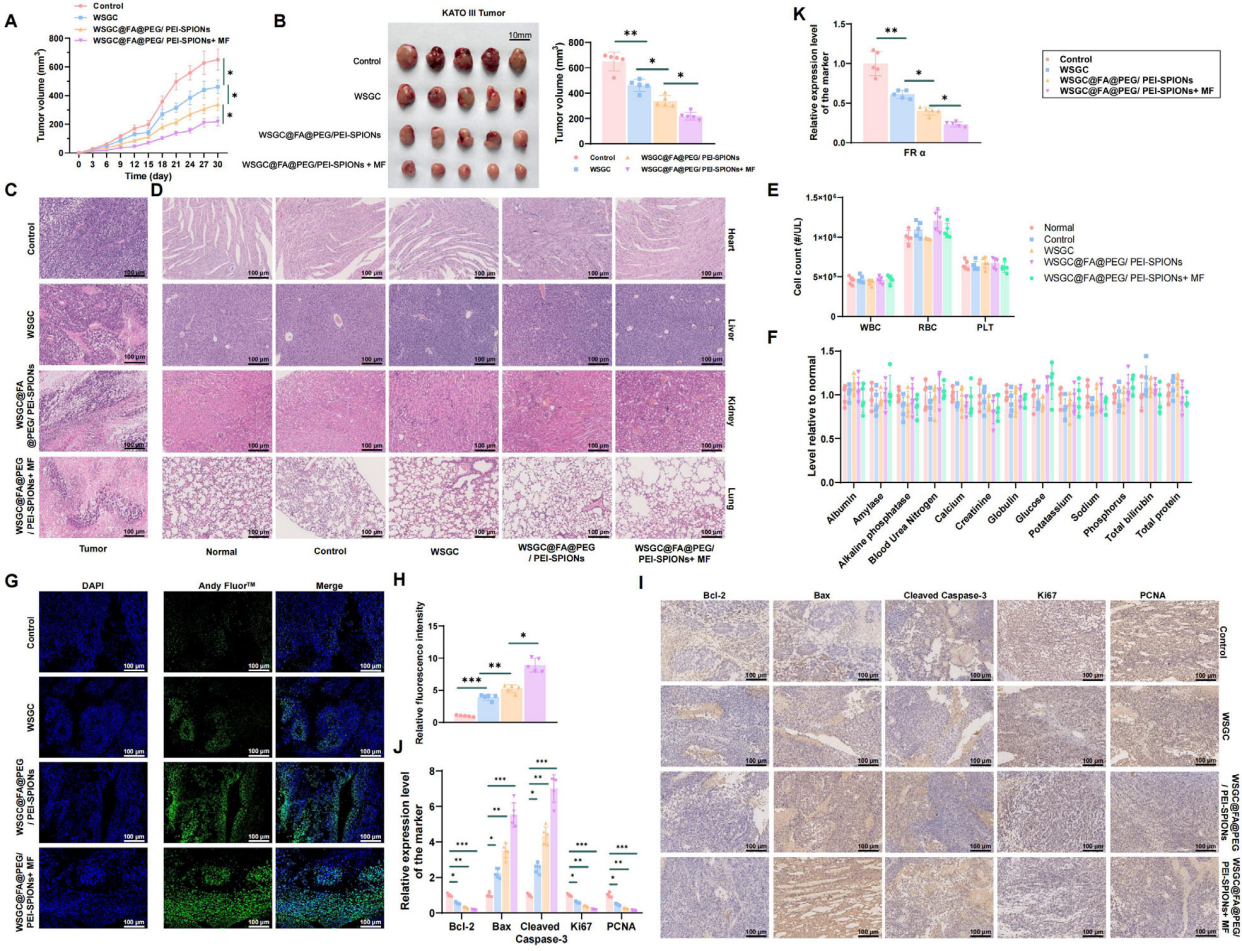
In vivo anti‐tumor efficacy of WSGC@FA@PEG/PEI‐SPIONs against KATO III tumors. Note: A) Changes in tumor volume over time with four different treatment modalities; B) Representative images of tumors collected on day 30 following the four different treatment modalities; C) Representative micrographs of H&E‐stained tumors showing reduced tumor cell count in the WSGC@FA@PEG/PEI‐SPIONs+ MF treatment group compared to the other three treatment modalities; D) Representative micrographs of H&E‐stained sections of four vital organs in normal mice (Normal) and in GA model mice treated with the four different modalities; E) Quantification of WBC, RBC, and PLT counts in blood samples from the same groups; F) Serum biochemistry parameters for each group, including albumin, amylase, alkaline phosphatase, blood urea nitrogen, calcium, creatinine, globulin, glucose, potassium, sodium, phosphorus, total bilirubin, and total protein; G) TUNEL assay to detect cell apoptosis in tumor tissues of KATO III tumor‐bearing mice; H) Statistical chart of apoptosis in each group; I) Immunohistochemical analysis of the expression levels of Bax, Cleaved Caspase‐3, Bcl‐2, Ki67, and PCNA proteins in tumor tissues of KATO III tumor‐bearing mice (scale bar = 100 µm); J) Statistical analysis of the immunohistochemical findings; K) RT‐qPCR results for FRα mRNA expression in tumor tissues of nude mice. * Indicates statistically significant differences between groups, with *p < 0.05, **p < 0.01; Animal number *n* = 5. Data were presented as mean ± SD. One‐way ANOVA was used to compare data among different time groups, while repeated measures ANOVA was applied for tumor volume comparisons at different time points.

To further evaluate the therapeutic potential of the nanoformulation, KATO III xenograft‐bearing mice were treated with WSGC@FA@PEG/PEI‐SPIONs at an equiv. WSGC dose of 80 nmol g⁻^1^. Compared with free WSGC, WSGC@FA@PEG/PEI‐SPIONs induced stronger tumor growth suppression, and the inclusion of a MF further enhanced this inhibitory effect (**Figure** **4**A,B; Figure S4A,B, Supporting Information).

Histological assessment via H&E staining revealed extensive tumor necrosis and significantly reduced viable tumor cell density in mice treated with WSGC@FA@PEG/PEI‐SPIONs, particularly under MF conditions (Figure 4C; Figure S4C, Supporting Information). To examine systemic toxicity, major organs (heart, liver, lung, and kidney) were analyzed histologically. No obvious pathological changes were observed across all treatment groups, indicating good biocompatibility and a lack of organ‐specific toxicity (Figure 4D; Figure S4D, Supporting Information). Hematological analysis further supported these findings, as WBC, RBC, and PLT counts remained within normal ranges and were comparable to control mice (Figure 4E). Serum biochemical indices, including albumin, amylase, alkaline phosphatase, BUN, calcium, creatinine, globulin, glucose, potassium, sodium, phosphorus, total bilirubin, and total protein, also showed no significant abnormalities (Figure S4F, Supporting Information), reinforcing the systemic safety of the treatment.

TUNEL staining demonstrated that apoptotic nuclei—visualized by overlapping blue (DAPI) and green (Andy Fluor) fluorescence—were significantly more abundant in tumor tissues from mice treated with WSGC@FA@PEG/PEI‐SPIONs compared to those treated with free WSGC. The apoptotic index was highest in the MF‐treated group, indicating enhanced apoptotic induction by the magnetic targeting strategy (Figure 4G,H; Figure S4G,H, Supporting Information).

Immunohistochemical analysis revealed increased expression of pro‐apoptotic proteins Bax and Cleaved Caspase‐3 in tumor tissues following WSGC@FA@PEG/PEI‐SPIONs treatment, with more substantial effects observed in the MF‐responsive group. In contrast, anti‐apoptotic Bcl‐2, and proliferation‐associated proteins Ki67 and PCNA, were significantly downregulated, suggesting that the treatment effectively inhibits tumor growth and induces apoptosis (Figure 4I,J; Figure S4I,J, Supporting Information). In addition, RT‐qPCR analysis showed that expression of FRα was significantly suppressed in tumors treated with WSGC or WSGC@FA@PEG/PEI‐SPIONs. The MF‐responsive group exhibited the most pronounced reduction in FRα mRNA expression (Figure 4K; Figure S4K, Supporting Information).

Collectively, these results confirm that the WSGC peptide exerts anti‐tumor effects in vivo, and its delivery via the FA‐targeted, PEG/PEI‐coated SPIONs significantly enhances therapeutic efficacy, especially when combined with magnetic targeting. The observed improvements in tumor suppression, along with the favorable safety profile, underscore the potential of WSGC@FA@PEG/PEI‐SPIONs as a promising and biocompatible nanotherapeutic strategy for GA treatment.

### 2.6 Biodistribution and MRI Evaluation of WSGC@FA@PEG/PEI‐SPIONs in Mice

**Figure 5:**
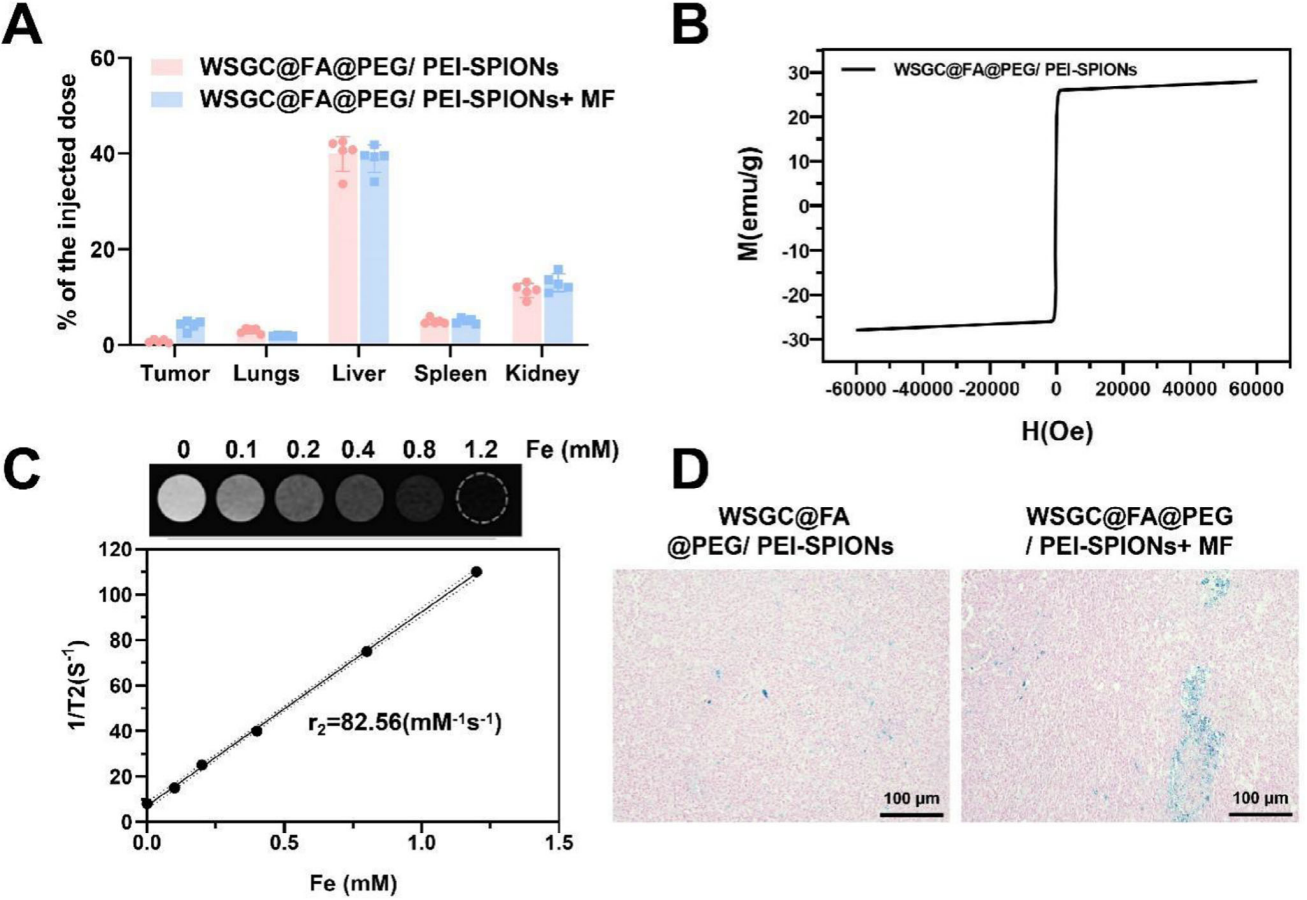
Biodistribution and magnetic properties of WSGC@FA@PEG/ PEI‐SPIONs. Note: A) Biodistribution of WSGC@FA@PEG/ PEI‐SPIONs in mice at 2 h post‐injection; B) Magnetization strength (M) of WSGC@FA@PEG/ PEI‐SPIONs under different MF intensities, characterizing their magnetic behavior and material hysteresis properties; C) T2‐weighted imaging using MRI at various iron concentrations (0.1, 0.2, 0.4, 0.8, and 1.2 mm), with linear fitting of 1/T2 values to evaluate the effect of iron concentration on T2 signal intensity; D) Histological examination of tumor tissues using Prussian Blue Staining. Animal number *n* = 5. Data were presented as Mean ± SD. Statistical significance between two groups was determined using an unpaired two‐tailed *t*‐test.

To investigate the magnetic targeting capability and biodistribution of WSGC@FA@PEG/PEI‐SPIONs in vivo, mice bearing subcutaneous tumors were administered the nanoparticle formulation in the presence or absence of an external MF. ICP‐OES was used to quantify iron accumulation in tumors and major organs, including the liver, spleen, kidneys, and lungs (**Figure** **5**A). In mice exposed to MF, iron accumulation in tumor tissues reached 4.108% of the injected dose, representing a 7.2‐fold increase compared to 0.574% in tumors of mice treated without MF exposure. The majority of SPIONs were cleared by the kidney and liver, with some clearance by the spleen. Meanwhile, no statistically significant differences in iron accumulation were observed in the liver, lungs, spleen, or kidneys between MF and non‐MF groups (Figure 5A).

The magnetic characteristics of WSGC@FA@PEG/PEI‐SPIONs were further evaluated using SQUID magnetometry. The magnetic hysteresis loop revealed a typical superparamagnetic behavior with negligible remanence and coercivity (Figure 5B). To determine the potential of WSGC@FA@PEG/PEI‐SPIONs as MRI contrast agents, T₂ relaxation measurements were performed using a 3.0 T MRI scanner. A clear inverse correlation between signal intensity and iron concentration was observed, indicating that WSGC@FA@PEG/PEI‐SPIONs function as efficient negative (T_2_) contrast agents. The transverse relaxivity (r_2_) was calculated to be 82.56 mm ^−1^ s^−1^ via linear regression analysis (Figure 5C).

Histological validation was performed by staining tumor tissue sections with Prussian Blue, which selectively labels iron‐containing particles. As shown in Figure 5D, the MF‐treated group exhibited dense blue‐stained areas within tumor sections, confirming substantial intratumoral accumulation of SPIONs. In contrast, tumors from mice treated without MF showed sparse blue staining, suggesting reduced nanoparticle retention in the absence of magnetic guidance.

The above results reveal that the MF significantly enhances the accumulation of WSGC@FA@PEG/PEI‐SPIONs at the tumor site, reduces their distribution in the liver and spleen, thereby improving the contrast effect of T_2_‐weighted MRI and aiding in the enhancement of tumor imaging diagnostics and therapeutic effects. These findings provide important scientific evidence for tumor‐targeted therapy and imaging based on MFs and superparamagnetic nanomaterials.

### 2.7 WSGC@FA@PEG/PEI‐SPIONs Inhibit GA Progression via the Notch Signaling Pathway

To thoroughly elucidate the potential molecular mechanisms underlying the anti‐tumor effects of WSGC@FA@PEG/PEI‐SPIONs, transcriptome sequencing was performed on GA tissues from untreated (Control) and treated (Treat) mice.

**Figure 6:**
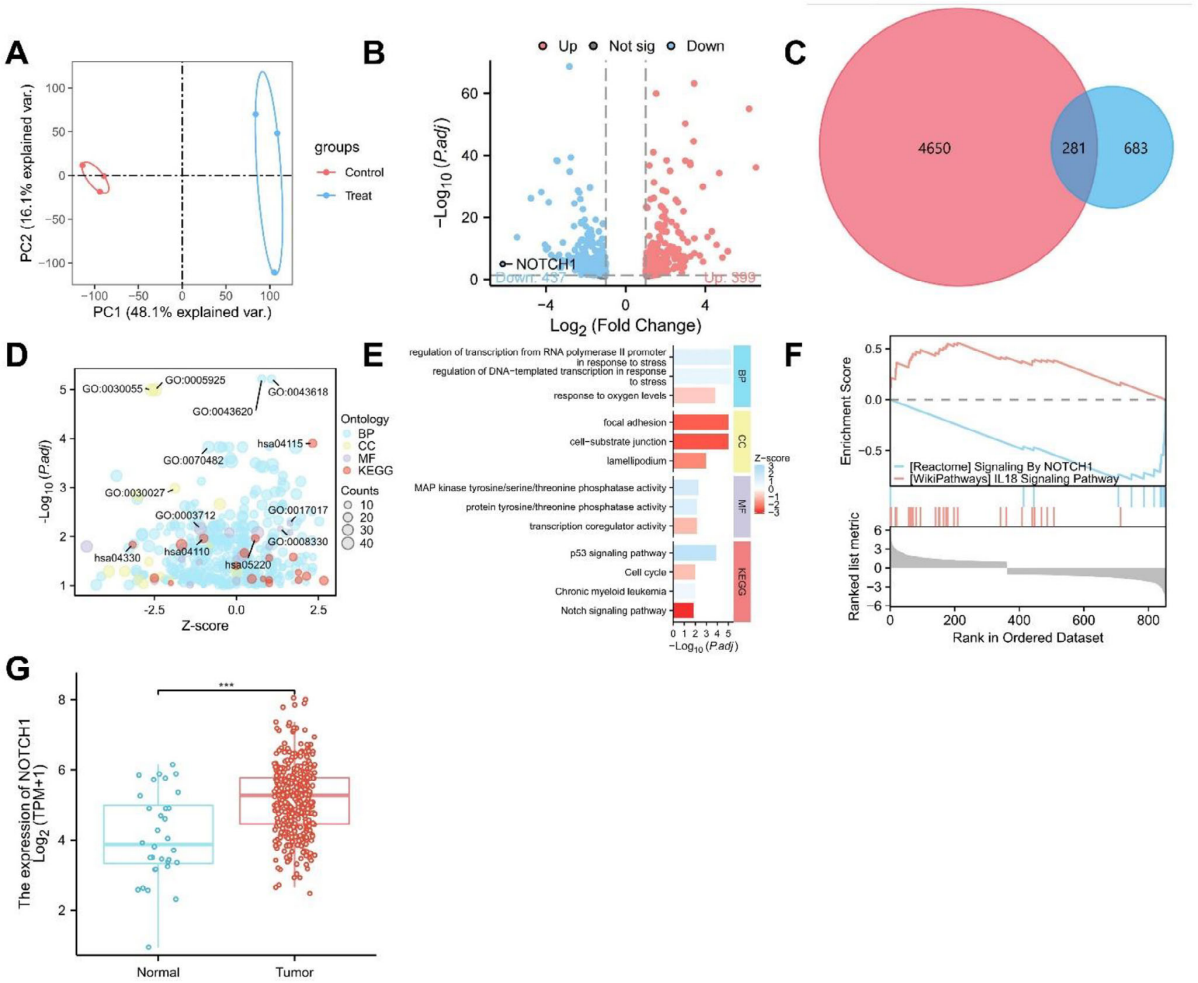
Transcriptomic analysis of WSGC@FA@PEG/PEI‐SPIONs in the treatment of GA. Note: A) PCA showing distinct separation of the Treat and Control groups on PC1 and PC2, explaining 64.2% of the total variance (48.1% and 16.1%); B) Analysis of DEGs illustrating gene expression changes in the Treat group relative to the Control group, with red dots representing upregulated genes and blue dots representing downregulated genes (Control = 3, Treat = 3); C) Venn diagram comparing DEGs and GA‐related genes from the Genecards database; D,E) Bubble charts of GO and KEGG pathway enrichment analysis, displaying significant enrichment of BP, CC, molecular function, and pathways (KEGG) for DEGs; F) GSEA revealing significantly enriched upregulated signaling pathways in the Treat group, particularly the Notch signaling pathway; G) Expression of NOTCH1 in normal tissues and GA tissues in TCGA‐STAD, represented using TPM (log2 transformed) values (Normal = 32, Tumor = 375); * Indicates statistically significant differences between groups, with ***p < 0.001; Control = 3, Treat = 3.

PCA revealed distinct transcriptomic profiles between the two groups, with PC1 and PC2 explaining 48.1% and 16.1% of the total variance, respectively. This clear separation demonstrates that treatment with WSGC@FA@PEG/PEI‐SPIONs significantly alters gene expression in GA tissues (**Figure** **6**A).

Differential expression analysis identified numerous DEGs between the Treat and Control groups. The volcano plot highlighted a subset of significantly upregulated (red) and downregulated (blue) genes in the Treat group. Notably, NOTCH1 expression was significantly downregulated, suggesting inhibition of the Notch signaling pathway following treatment (Figure 6B).

To further explore the involvement of the Notch pathway, a Venn diagram was constructed to compare the identified DEGs with Notch pathway‐associated genes from the GeneCards database. A total of 281 overlapping genes were identified, reinforcing the hypothesis that WSGC@FA@PEG/PEI‐SPIONs exert their therapeutic effect, at least in part, through modulation of the Notch pathway (Figure 6C).

GO and KEGG enrichment analyses revealed that DEGs were significantly enriched in pathways related to transcriptional regulation, cell adhesion, MAP kinase signaling, and the Notch signaling pathway, further supporting the importance of the Notch signaling pathway in the WSGC@FA@PEG/PEI‐SPIONs treatment process (Figure 6D,E).

GSEA (Figure 6F) provided additional validation, revealing that the Notch signaling pathway was significantly downregulated in the Treat group compared to the Control, thereby confirming the suppressive effect of WSGC@FA@PEG/PEI‐SPIONs on this pathway (Figure 6F).

Expression data extracted from TCGA revealed significantly elevated NOTCH1 mRNA levels in GA tissues relative to normal gastric tissues (*p* < 0.001), highlighting the oncogenic role of NOTCH1 in GA and reinforcing its potential as a therapeutic target (Figure 6G).

In conclusion, based on transcriptomic analysis, we propose that WSGC@FA@PEG/PEI‐SPIONs likely regulate the development of GA by inhibiting the Notch signaling pathway.

### 2.8 WSGC Peptide Reverses Chemotherapy Resistance in GA by Inhibiting the Notch Signaling Pathway‐Mediated Mitophagy

The Notch signaling pathway plays a critical regulatory role in various physiological and pathological processes, including cell differentiation, proliferation, and apoptosis.^[^ ^58^ ^]^ In GA, aberrant activation of the Notch pathway is strongly associated with tumor progression and poor clinical outcomes. Previous studies have demonstrated that hyperactivation of Notch signaling enhances tumor cell proliferation, invasion, and chemoresistance,^[^ ^59^ ^]^ while targeted inhibition of this pathway can sensitize tumor cells to chemotherapeutic agents.^[^ ^60^ ^]^ Specifically, silencing of NOTCH1 has been shown to suppress GA cell growth and invasion, highlighting the therapeutic potential of Notch inhibition in overcoming drug resistance.^[^ ^59^ ^]^ In the present study, we explored the effect of the WSGC peptide on chemotherapy resistance in GA and its regulation of the Notch signaling pathway.

To assess the clinical relevance of NOTCH1 expression in GA, we conducted Kaplan‐Meier survival analysis using patient datasets. The results demonstrated that high NOTCH1 expression was significantly correlated with poorer overall survival. The survival rate of patients with low NOTCH1 expression (black curve) was markedly higher than that of patients with high expression (red curve). Statistical analysis revealed a hazard ratio (HR) of 1.38 (95% CI: 1.16‐1.64, *p* = 0.00024, log‐rank test), indicating a strong prognostic association (Figure S5A, Supporting Information).

To further validate the relationship between Notch signaling and chemoresistance, we established CDDP‐resistant GA cell lines, KATO III R‐CDDP and MNK‐45 R‐CDDP, and compared their drug response profiles with those of their respective WT parental lines. Cell viability assays were performed to generate dose‐response curves (Figure S5B,C, Supporting Information), and EC_50_ values were determined via nonlinear regression (**Table** **1**). The RI (Table 1), defined as the ratio of EC_50_ in resistant cells to that in parental cells, was calculated. Compared to the parental lines, KATO III R‐CDDP and MNK‐45 R‐CDDP displayed significantly increased tolerance to CDDP, with RIs of 5.3 and 6.8, respectively.

**Table 1:** EC50 and resistance index values of parental cells and CDDP‐resistant cells.

Parental Cell Lines	EC50 [µm]	Resistant Cell Lines	EC50 [µm]	RI
AGS WT	4.45 ± 0.87	AGS R‐CDDP	23.26 ± 0.45^*^	5.3
KATO III WT	4.95 ± 1.95	KATO III R‐CDDP	33.61 ± 1.02^*^	6.8

*Note*: EC: Effective concentration (the drug concentration in µm that inhibits 50% of cell growth). Resistance Index WT: Wild‐type or parental cell line; CDDP: Cisplatin.

To quantify the extent of cell death induced by CDDP, we conducted flow cytometric analysis using Annexin V/PI dual staining. As shown in Figure S5D (Supporting Information), the percentage of apoptotic and necrotic cells (Annexin V^+^/PI^−^ and Annexin V^+^/PI^+^, respectively) was significantly lower in CDDP‐resistant cells compared to their wild‐type counterparts (*p* < 0.001). Representative flow cytometry plots confirmed this trend, showing a marked reduction in cell death in KATO III R‐CDDP cells relative to KATO III WT cells (Figure S5E,F, Supporting Information). Control groups (untreated with CDDP) for KATO III WT and KATO III R‐CDDP cells are displayed in Figure S5G,H (Supporting Information).

Similarly, the percentage of cell death in MNK‐45 R‐CDDP cells (overall death rate: 40.8%) was significantly lower than that observed in parental MNK‐45 cells (59.2%) (*p *< 0.01; Figure S5I, Supporting Information). Representative flow cytometry dot plots confirmed this difference, revealing increased cell death and decreased viability in wild‐type MNK‐45 cells compared to MNK‐45 R‐CDDP cells (Figure S5J vs Figure S5K, Supporting Information). Control plots for untreated MNK‐45 WT and MNK‐45 R‐CDDP cells are provided in Figure S5L,M (Supporting Information). Previous studies have identified ABCC2, ATP7A, and CTR1 as key markers associated with CDDP resistance.^[^ ^61^ ^]^ Consistently, we found significantly higher expression levels of ABCC2 and ATP7A in KATO III R‐CDDP and MNK‐45 R‐CDDP cells compared to their parental lines. In contrast, CTR1 expression was markedly decreased in the resistant cell lines (Figure S5N,O, Supporting Information), confirming the successful establishment of cisplatin‐resistant GA models.

Transcriptome analysis revealed that Notch1 was significantly downregulated in the polypeptide‐treated group (log_2_FC = − 6.17, *p* < 0.001), suggesting a role for WSGC peptide in modulating chemoresistance via inhibition of the Notch signaling pathway.

**Figure 7:**
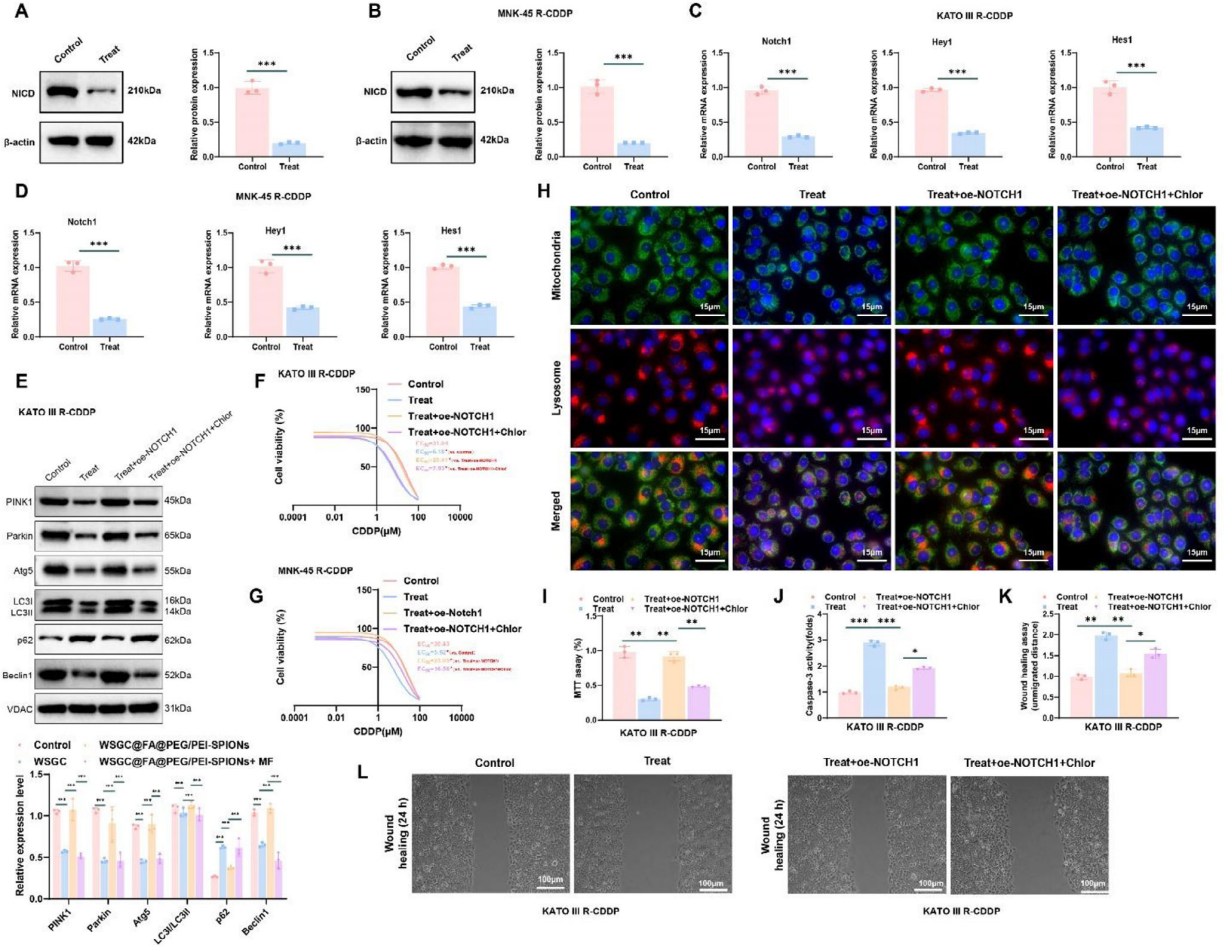
WSGC peptide inhibits mitophagy through the Notch signaling pathway. Note: A) Protein expression of NICD in KATO III R‐CDDP cells was analyzed by immunoblotting (*n* = 3); B) mRNA expression of Notch1 and its target genes Hey1 and Hes1 in KATO III R‐CDDP cells was measured (*n* = 3); C) Expression of NICD in KATO III R‐CDDP cells across different groups was analyzed; D) mRNA expression of Notch1 and its target genes Hey1 and Hes1 in various groups of GA cells in KATO III R‐CDDP cells was detected; E) Protein expression of mitophagy‐related proteins (PINK1, Parkin, Atg5, LC3B, p62, Beclin1) in KATO III R‐CDDP cells was assessed; F,G) Dose‐response curves of KATO III R‐CDDP and MNK‐45 R‐CDDP cell lines treated with CDDP; H) Co‐localization of mitochondria (green) and lysosomes (red) in KATO III R‐CDDP cells was observed in different treatment groups by immunofluorescence staining (scale bar = 15 µm); I) Cell viability of KATO III R‐CDDP cells was determined using the MTT assay; J) Changes in Caspase‐3 activity in KATO III R‐CDDP cells after different treatments were measured; K,L) Scratch assay was performed to evaluate cell migration in KATO III R‐CDDP cells 24 h post‐treatment; *p < 0.05, **p < 0.01, ***p < 0.001. All cell experiments were performed in triplicate (*n* = 3), and results are expressed as Mean ± SD. For comparisons between two groups, an independent samples *t*‐test was used. For comparisons among three or more groups, one‐way ANOVA was performed.

To validate this hypothesis, we treated drug‐resistant GA cells with WSGC@FA@PEG/PEI‐SPIONs. Western blot analysis showed a substantial reduction in the intracellular domain of Notch1 (NICD) in the treatment group compared to the control, indicating effective pathway inhibition (**Figure** **7**A,B). Furthermore, RT‐qPCR analysis confirmed significant downregulation of Notch1, Hey1, and Hes1 mRNA levels in both KATO III and MNK‐45 cells (Figure 7C,D).

Notably, the Notch pathway has been implicated in the regulation of mitophagy through various mechanisms.^[^ ^62^, ^63^ ^]^ To examine whether WSGC peptide impacts mitochondrial function via this pathway, we investigated mitophagy‐related markers in KATO III R‐CDDP cells. We observed that WSGC peptide treatment led to decreased expression of PINK1, Parkin, and LC3‐II, along with increased expression of p62, Beclin1, and Atg5. These changes were reversed by Notch1 overexpression. Furthermore, co‐treatment with the autophagy inhibitor Chloroquine^[^ ^64^ ^]^ yielded a similar trend, suggesting that WSGC peptide suppresses mitophagy through Notch pathway inhibition (Figure 7E; Figure S6A, Supporting Information).

Dose‐response assays revealed that WSGC peptide significantly sensitized KATO III R‐CDDP and MNK‐45 R‐CDDP cells to CDDP treatment compared to controls. However, this sensitization effect was abrogated by Notch1 overexpression and restored upon addition of Chloroquine (Figure 7F,G).

To further verify the inhibitory effect on mitophagy, we performed mitochondrial and lysosomal co‐staining. WSGC peptide reduced the degree of colocalization between mitochondria and lysosomes, an effect reversed by Notch1 overexpression but mimicked by Chloroquine (Figure 7H; Figure S6B, Supporting Information). However, the oe‐NOTCH1 group reversed the effect of polypeptide, indicating that polypeptide inhibits the onset of mitophagy.

Functional assays were conducted to evaluate the consequences of mitophagy inhibition. MTT assays demonstrated increased viability in Notch1‐overexpressing cells, which was reversed by Chloroquine. Correspondingly, caspase‐3 activity, an indicator of apoptosis, was suppressed by Notch1 overexpression but restored upon Chloroquine treatment (Figure 7I,J; Figure S6C,D, Supporting Information). The scratch wound assay revealed that WSGC peptide significantly impaired cell migration, a suppressive effect reversed by Notch1 overexpression and subsequently re‐inhibited by Chloroquine, suggesting that mitophagy contributes to the migratory capacity of drug‐resistant GA cells (Figure 7K,L; Figure S6E,F, Supporting Information).

In conclusion, WSGC peptide reverses cisplatin resistance in GA cells by downregulating Notch signaling—specifically reducing NICD and downstream transcription factors—and inhibiting mitophagy. The modulatory role of WSGC in chemoresistance and mitophagy has been clearly demonstrated through rescue experiments involving Notch1 overexpression and autophagy inhibition. These findings establish a solid foundation for further development of WSGC‐based therapeutic strategies targeting Notch‐mediated mitophagy in GA.

### 2.9 In Vivo Validation of WSGC Peptide‐Mediated Mitophagy Inhibition via the Notch Signaling Pathway

**Figure 8:**
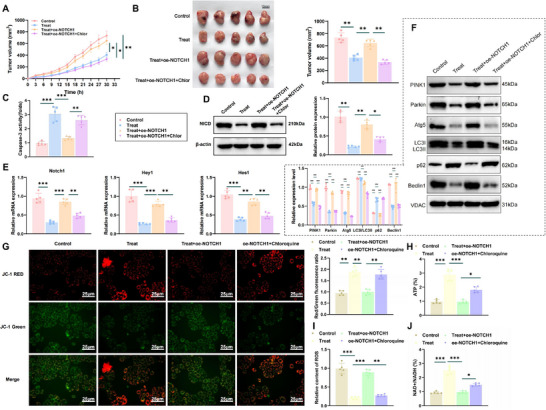
WSGC peptide mediates mitophagy through the Notch signaling pathway. Note: A) Tumor growth curves in mice from different groups; B) Subcutaneous xenograft tumors in nude mice (left) and corresponding weight statistics (right) in different groups; C) Changes in Cleaved Caspase‐3 activity; D) Protein expression analysis of NICD by immunoblotting (*n* = 5); E) Detection of mRNA expression of Notch1 and its target genes Hey1 and Hes1 in GA cells in various groups; F) Western blot analysis and statistical results of mitophagy‐related proteins (PINK1, Parkin, Atg5, LC3II, p62, Beclin1); G) Measurement of MMP; H) Determination of ATP levels; I) Assessment of ROS levels; J) Measurement of NAD+/NADH ratio; *p < 0.05, **p < 0.01, ***p < 0.001. Animal experiments involved *n* = 5. Data were presented as mean ± SD. One‐way ANOVA was used to compare data among different time groups, while repeated measures ANOVA was applied for tumor volume comparisons at different time points.

To further validate the in vivo effect of WSGC peptide on mitophagy inhibition mediated through the Notch signaling pathway, we established a GA xenograft mouse model. Tumor volume measurements demonstrated that mice treated with the WSGC peptide exhibited significantly reduced tumor sizes compared to controls, indicating potent tumor growth inhibition by the peptide. However, this inhibitory effect was reversed upon Notch1 overexpression (Treat+oe‐NOTCH1). Notably, co‐administration of the autophagy inhibitor Chloroquine restored the anti‐tumor efficacy of the WSGC peptide despite Notch1 overexpression (**Figure** **8**A,B).

Moreover, the Caspase‐3 activity assays revealed that the WSGC peptide significantly increased Caspase‐3 activity relative to the control group, suggesting enhanced apoptosis. This effect was attenuated by Notch1 overexpression but recovered upon Chloroquine treatment (Figure 8C).

Assessment of the Notch signaling pathway showed that WSGC peptide treatment led to significant downregulation of NICD protein and its downstream targets Notch1, Hey1, and Hes1. Overexpression of Notch1 reversed this downregulation, while Chloroquine addition restored the inhibitory effect of WSGC on these gene products (Figure 8D,E).

Regarding mitophagy, WSGC peptide treatment markedly decreased the expression levels of key mitophagy‐related proteins, including PINK1, Parkin, Atg5, LC3‐II, and Beclin1, while increasing p62, a marker of mitophagy inhibition. These effects were partially reversed by Notch1 overexpression and reestablished with Chloroquine co‐treatment (Figure 8F).

Mitophagy, a selective autophagic process that removes damaged mitochondria, plays a pivotal role in maintaining mitochondrial quality and cellular homeostasis.^[^ ^65^ ^]^ Functional assays revealed that WSGC peptide‐treated tumors displayed enhanced mitochondrial function (Figure 8G,H), reduced intracellular reactive oxygen species (Figure 8I), and an elevated NAD^+^/NADH ratio (Figure 8J). These improvements were mitigated by Notch1 overexpression and subsequently restored with Chloroquine administration, further supporting the involvement of mitophagy regulation in the observed therapeutic effects.

In conclusion, these in vivo findings confirm that WSGC peptide exerts its antitumor effects by inhibiting Notch signaling, thereby suppressing mitophagy and restoring mitochondrial function and cellular homeostasis. These findings provide a critical foundation for further development of polypeptide‐based anticancer therapies.

## 3 Discussion

This study systematically investigated the anti‐tumor efficacy of WSGC@FA@PEG/PEI‐SPIONs nanoparticles in GA through a comprehensive series of in vitro and in vivo experiments. By simultaneously targeting the Notch signaling pathway and mitophagy, we propose a novel and multi‐pronged therapeutic strategy to enhance anti‐cancer effects and reverse chemotherapy resistance in GA.

A primary finding of this study is that WSGC@FA@PEG/PEI‐SPIONs significantly suppressed the proliferation, migration, and invasion of GA cells. This anti‐tumor effect was mediated through the inhibition of the Notch signaling pathway, leading to downregulation of key mitophagy‐associated proteins, including LC3, PINK1, and Parkin. These results highlight the dual regulatory role of WSGC@FA@PEG/PEI‐SPIONs in modulating both Notch signaling and mitophagy, providing a comprehensive therapeutic strategy for curbing tumor progression and overcoming chemoresistance. Emerging evidence supports the potential of nanodrug formulations with stimuli‐responsive release mechanisms to enhance drug accumulation at tumor sites, improve bioavailability, and reduce systemic toxicity.^[^ ^66^ ^]^ Furthermore, targeted nanomedicines have demonstrated efficacy in reshaping the immunosuppressive tumor microenvironment (TME), thereby amplifying cancer immunotherapy outcomes.^[^ ^67^ ^]^ In contrast to previous approaches, which typically focused on single targets or isolated signaling pathways, the strategy presented in this study leverages nanotechnology and peptide functionalization to achieve multi‐targeted modulation, enhancing both anti‐tumor efficacy and resistance reversal. Traditional chemotherapy or single‐target treatments often fail to surmount the challenges posed by the complex and adaptive TME, rendering them less effective in advanced‐stage cancers.^[^ ^68^, ^69^ ^]^

In the context of tumor‐targeted therapy, the application of the WSGC peptide in this study demonstrated distinct advantages. Notably, WSGC exhibited selective binding to GA cells, while the FA@PEG/PEI modification further improved the stability and biocompatibility of the nanoparticle formulation. The integration of targeting specificity, efficient drug loading, and controlled release within the WSGC@FA@PEG/PEI‐SPIONs system represents a significant advancement over conventional targeted therapeutic approaches. While tumor‐homing peptides such as RGD and TAT have shown promising targeting efficacy in various cancers, their limitations in multifunctional delivery and drug resistance modulation underscore the innovation of the WSGC@FA@PEG/PEI‐SPIONs design.^[^ ^70^, ^71^ ^]^

Another key contribution of this study lies in elucidating the role of the Notch signaling pathway in mediating chemotherapy resistance. Aberrant activation of Notch in GA has been extensively reported and is known to facilitate tumor cell proliferation, differentiation, and resistance to apoptosis.^[^ ^72^, ^73^ ^]^ Our findings extend this understanding by uncovering a novel mechanistic link between Notch signaling and mitophagy‐driven drug resistance. Specifically, inhibition of Notch signaling led to a marked downregulation of mitophagy‐associated proteins, revealing a previously underexplored pathway through which Notch contributes to chemoresistance. These insights broaden the current understanding of Notch signaling in tumor biology and highlight new potential therapeutic targets for reversing drug resistance in GA.

Mitophagy, a selective form of autophagy, plays a crucial role in maintaining cellular homeostasis and facilitating stress adaptation.^[^ ^74^, ^75^ ^]^ Previous studies have demonstrated that certain cancer cells exploit mitophagy as a survival mechanism to evade chemotherapy‐induced apoptosis.^[^ ^76^, ^77^ ^]^ In our study, treatment with WSGC@FA@PEG/PEI‐SPIONs led to a marked downregulation of key mitophagy‐related proteins, including LC3, PINK1, and Parkin, effectively suppressing mitophagic activity in GA cells and reversing their chemotherapy resistance. These findings are consistent with existing literature on the role of mitophagy in drug resistance, while also emphasizing the superior precision and efficacy of nanoparticle‐based regulation as a therapeutic strategy.

Our comprehensive in vitro and in vivo experiments validated the anti‐tumor efficacy and biosafety of WSGC@FA@PEG/PEI‐SPIONs. In a GA xenograft nude mouse model, the nanoparticles significantly inhibited tumor growth and promoted apoptosis. Compared to other nanodrug systems, WSGC@FA@PEG/PEI‐SPIONs demonstrated enhanced tumor targeting, efficient drug delivery, and sustained release, highlighting their potential as an advanced nanoplatform for cancer therapy.

Mechanistically, WSGC@FA@PEG/PEI‐SPIONs inhibited the Notch signaling pathway by downregulating NICD, the active form of Notch1, along with its downstream effectors Hey1 and Hes1, thereby further reducing the expression of mitophagy‐associated proteins. These results align with prior studies indicating that Notch signaling promotes tumor proliferation and chemoresistance. Moreover, our transcriptomic profiling offered a comprehensive molecular overview, reinforcing the role of Notch‐mitophagy crosstalk in GA progression and identifying novel molecular targets for therapeutic intervention.

While this study underscores the therapeutic potential of WSGC@FA@PEG/PEI‐SPIONs in overcoming chemotherapy resistance in GA, several limitations must be acknowledged. First, the majority of experiments were performed in in vitro cell lines and murine xenograft models, without the inclusion of large preclinical animal models or clinical trials. This raises concerns regarding the clinical translatability of the findings. Second, the long‐term biosafety, systemic toxicity, and pharmacokinetics of the nanoparticles remain inadequately characterized, which is essential for clinical application. Last, the relatively small sample size in some experimental groups may limit the statistical power and generalizability of the results, warranting validation in larger cohorts.

Future studies should aim to address these limitations by evaluating the anti‐tumor efficacy and toxicity profiles of WSGC@FA@PEG/PEI‐SPIONs in larger preclinical models, performing chronic toxicity and long‐term biodistribution analyses, and refining the nanoparticle synthesis process to reduce production costs and improve scalability. Additionally, exploring the therapeutic applicability of this nanoplatform across diverse tumor types could substantiate its broad‐spectrum anticancer potential. Strategic collaboration with clinical researchers and pharmaceutical industries will be essential to accelerate the translation of these findings into viable clinical interventions.

## 4 Experimental Section

### Materials

Folic acid (FA) (F7876), Iron(III) acetylacetonate (Fe(acac)3, 98%) (F300), 1‐(3‐Dimethylaminopropyl)‐3‐ethyl carbodiimide hydrochloride (EDC, 97%) (0 3450), N‐Hydroxysulfosuccinimide sodium salt (sulfo‐NHS, 98%) (56 485), Chloroquine (C6628), 4‐Dimethylaminopyridine (DMAP) (39 405), Succinic anhydride (239 690), Polyethylenimine (PEI, molecular weight = 1800) (408 700), and Polyethylene glycol (PEG, molecular weight = 1000) (8.07488) were all provided by Sigma‐Aldrich (USA). All reagents were used without further purification.

### Synthesis and Source of WSGC Peptide

The research group previously identified a polypeptide with a molecular weight of 6 449 Da (sequence: MQPRVLALLASADASLLSFMYMKHATKTAKDVQVAQQARGWVTDGFSSL), derived from apoC‐III, as a specific non‐inflammatory serum biomarker for GA. This 60‐amino acid polypeptide complements traditional GA tumor markers and may aid in diagnosis and prognosis. Further investigation revealed that the bioactive region lies within a 40‐amino acid sequence outside the signal peptide, which we named the WSGC peptide (protected by patent). Its sequence is SEAEDASLLSFMQGYMKHATKTAKDALSSVQESQVAQQAR, with a molecular weight of 4 344 Da and favorable water solubility. The WSGC peptide, its fluorescein‐labeled version (WSGC‐FITC), and a control peptide (MK‐21) were synthesized by Beijing Zexiyuan Biotech Co., Ltd. using the solid‐phase peptide synthesis (SPPS) method.

The synthesis steps are outlined below: (1) Wang resin (13 618, Sigma‐Aldrich, USA) was used as the solid support. The resin was swollen in dichloromethane (DCM, 650 463, Sigma‐Aldrich, USA), then washed multiple times with N,N‐dimethylformamide (DMF, 1 601 500, Sigma‐Aldrich, USA); (2) The 9‐fluorenylmethyloxycarbonyl (Fmoc) protecting group was removed using 20% piperidine (80 645, Sigma‐Aldrich, USA), followed by DMF washes to eliminate residual reagent; (3) Amino Acid Coupling: Amino acids protected with Fmoc were sequentially coupled using O‐benzotriazole‐N,N,N′,N′‐tetramethyluronium tetrafluoroborate (TBTU, 8.51008, Sigma‐Aldrich, USA) as the activating agent. Peptide bond formation was confirmed by ninhydrin colorimetric assay (53 940, Sigma‐Aldrich, USA). Excess reagents were removed by repeated DMF washes; (4) Peptide Chain Elongation: Using the sequences of WSGC, WSGC‐FITC, and MK‐21 as templates, amino acids were added one by one, with deprotection and coupling steps repeated accordingly; (5) Final deprotection: The terminal Fmoc group was removed using 20% piperidine; (6) Peptide cleavage: The completed polypeptides were cleaved from the resin using trifluoroacetic acid (TFA, T6508, Sigma‐Aldrich, USA), yielding the crude peptides; (7) Mass Spectrometry Verification: The crude products were analyzed using electrospray ionization mass spectrometry (ESI‐MS) to verify sequence accuracy; (8) Purification: Peptides were purified via high‐performance liquid chromatography (HPLC) to achieve a purity exceeding 95%.

### Preparation of WSGC@FA@PEG/PEI‐SPIONs

Synthesis of FA@PEG/PEI‐SPIONs: PEG/PEI‐SPIONs were synthesized using a modified polyol thermal decomposition method. Briefly, 0.3 g of PEI (molecular weight = 1 800) and 0.7 g of Fe(acac)₃ were added to 15.0 g of PEG‐1000. The mixture was then heated to 260 °C under a nitrogen atmosphere with constant stirring until complete reaction occurred. To functionalize the nanoparticle surface with FA, 0.199 mmol of FA was dissolved in 25 mL of DMSO. To activate the carboxyl groups of FA, 0.398 mmol of EDC and 0.398 mmol of sulfo‐NHS were added to the solution, which was stirred for 3 h at room temperature. Subsequently, the prepared PEG/PEI‐SPIONs were added dropwise to the FA‐active ester solution under vigorous stirring and allowed to react for 24 h. The functionalized nanoparticles were then separated using LS magnetic separation columns (130‐042‐401, Miltenyi). To remove unbound FA and residual solvents, the resulting solution was purified using a dialysis membrane (molecular weight cutoff: 8 000–14 000 Da; MD10(8000‐14000)D, Sartorius, USA) until no ultraviolet absorption was detected in the dialysate. The final product, FA@PEG/PEI‐SPIONs, was stored for further use.

Synthesis of WSGC@FA@PEG/PEI‐SPIONs: To prepare the final nanoparticle formulation, a defined volume of FA@PEG/PEI‐SPIONs solution was combined with the WSGC peptide in an equimolar ratio to FA. The peptide was pre‐activated with EDC and NHS to ensure efficient covalent conjugation. The mixture was stirred vigorously at room temperature for 24 h. Unreacted peptides and residual reagents were removed using LS magnetic separation columns. The purified nanoparticles were then dialyzed in deionized water (MWCO: 8 000–14 000 Da) for 24–48 h to eliminate low‐molecular‐weight impurities. The dialyzed WSGC@FA@PEG/PEI‐SPIONs were lyophilized into powder form and stored at − 20 °C for future use.

### Characterization of WSGC@FA@PEG/PEI‐SPIONs

The morphology of the iron oxide cores was examined using transmission electron microscopy (TEM; JEM‐2100F, JEOL) at an accelerating voltage of 200 kV. Zeta potential and hydrodynamic diameter were measured for PEG/PEI‐SPIONs, FA@PEG/PEI‐SPIONs, and WSGC@FA@PEG/PEI‐SPIONs using dynamic light scattering (DLS; Nano ZS90, Malvern). Samples were diluted tenfold in PBS and equilibrated for 60 s before measurement. Three measurements were taken at a scattering angle of 90°, and the polydispersity index (PDI) was recorded. Magnetic properties of the nanoparticles were assessed using a superconducting quantum interference device (SQUID; Quantum Design MPMS XL‐7). Iron content was quantified via inductively coupled plasma optical emission spectroscopy (ICP‐OES; Optima 8000, PerkinElmer). The composition and surface chemistry of the nanoparticles were further characterized using X‐ray photoelectron spectroscopy (XPS; Thermo ESCALAB 250).

### Release Kinetics of WSGC@FA@PEG/PEI‐SPIONs Nanoparticles

To evaluate release kinetics, 1 mL of WSGC@FA@PEG/PEI‐SPIONs micelle solution was sealed in a dialysis bag (MWCO: 5 000 Da) and immersed in 31 mL of PBS (pH 7.4) at 37 °C under gentle stirring. At predetermined time points, aliquots were collected, and the concentration of released doxorubicin (DOX) was measured using a UV–vis spectrophotometer. The cumulative release rate was calculated as the ratio of the DOX released at each time point to the initial DOX content in the micelles.

### Drug Loading Capacity (DLC) and Drug Loading Efficiency (DLE)

To evaluate the drug loading performance of the nanoparticles, 10 mg of WSGC peptide was dissolved in 5 mL of phosphate‐buffered saline (PBS, pH 7.4) and mixed with 5 mL of FA@PEG/PEI‐SPIONs solution (iron concentration: 2 mg mL⁻^1^). The mixture was incubated at 37 °C in the dark for 12 h. Subsequently, the drug‐loaded nanoparticles were collected using a magnetic separator. The unbound peptide in the supernatant was quantified by measuring absorbance at 480 nm using a UV–vis spectrophotometer (Shimadzu UV‐3600), based on a previously established standard curve for WSGC peptide concentrations.

The DLC and DLE are calculated using the following formulas: DLC (wt.%) = (W1/(W1 + W2)) × 100%; DLE (wt.%) = (W1/W3) × 100%, where W1 represents the mass of the loaded drug, W2 is the mass of FA‐SPIONs, and W3 is the mass of the initial drug.

### Measurement of Polypeptide Release of WSGC@FA@PEG/PEI‐SPIONs

In vitro drug release was assessed using a dialysis‐based method to evaluate the peptide release profile under various pH conditions. A 2 mL solution of WSGC@FA@PEG/PEI‐SPIONs containing 5 mg of WSGC peptide was sealed in a Pur‐A‐Lyzer Maxi dialysis device (MWCO = 50 kDa, PURX50005, Sigma‐Aldrich, USA). Dialysis was carried out against 50 mL of PBS at pH 5.0, 6.0, or 7.4, in a shaking incubator maintained at 37 °C. At predetermined time intervals, 1 mL of the external release medium was withdrawn and replaced with an equal volume of fresh PBS to maintain sink conditions. The concentration of released peptide was quantified using UV spectrophotometry, and the cumulative release percentage was calculated.

### Stability Assessment of WSGC@FA@PEG/PEI‐SPIONs

The colloidal and chemical stability of the WSGC@FA@PEG/PEI‐SPIONs formulation was evaluated under different conditions: in 0.01 mol L⁻^1^ PBS; in PBS containing 10% FBS (F8687, Sigma‐Aldrich, USA); in complete cell culture medium (RPMI‐1640 supplemented with 10% FBS). Samples were incubated at 37 °C for 72 h or stored at 4 °C for 28 days. Particle size changes over time were monitored using a nanoparticle tracking or spraying instrument. Additionally, WSGC peptide content in the supernatant was measured via UV spectrophotometry.

### Cell Culture

Normal human gastric epithelial cells (GES‐1) were obtained from Biyun Tian Company and cultured in a complete human gastric epithelial cell medium (CM‐H048, Pronuose). Human GA cell lines KATO III (HTB‐103) were acquired from the American Type Culture Collection (ATCC), and MKN‐45 (CL‐0292) cells were sourced from Wuhan Pricella Biotechnology Co., Ltd. Both KATO III and MKN‐45 cells were cultured in RPMI‐1640 medium (R4130, Sigma‐Aldrich, USA) supplemented with 10% FBS (TMS‐016, Sigma‐Aldrich, USA). All cell lines were maintained at 37 °C in a humidified incubator with 5% CO_2_.

### In Vitro Assessment of Cell Cytotoxicity

The cytotoxic effects of the WSGC peptide and nanoparticle formulations were evaluated in human GA cell lines KATO III and MKN‐45 using a 3‐(4,5‐dimethylthiazol‐2‐yl)‐2,5‐diphenyltetrazolium bromide (MTT)‐based in vitro toxicology assay kit (TOX1, Sigma‐Aldrich, USA). Cells in the exponential growth phase were seeded into 96‐well plates at a density of 4 × 10^3^ cells per well. After 24 h of incubation, cells were treated with 200 µL of fresh culture medium containing WSGC peptide (final concentration: 600 µm) for 24, 48, and 72 h. Cells treated with culture medium containing 0.1% DMSO (34 943, Sigma‐Aldrich, USA) served as the control.

To assess the cytotoxicity of both blank and drug‐loaded nanoparticles, KATO III and MKN‐45 cells were seeded and allowed to adhere overnight. The medium was then replaced with 200 µL of fresh medium containing PEG/PEI‐SPIONs, WSGC peptide, FA@PEG/PEI‐SPIONs, or WSGC@FA@PEG/PEI‐SPIONs (Fe concentration: 50 µg mL⁻^1^; WSGC concentration: 600 µm). After 24 h of treatment, 10 µL of MTT solution (5 mg mL⁻^1^) was added to each well, and the cells were incubated for an additional 4 h in the dark. Following incubation, 100 µL of DMSO was added to dissolve the resulting formazan crystals. Cell viability was quantified using a microplate reader (BioTek ELx800, Winooski, VT, USA) by measuring absorbance at 570 nm. The percentage of viable cells was calculated using the following formula: cell viability (5) = (Experimental group OD – Blank group OD) / (Control group OD – Blank group OD).^[^ ^40^, ^41^ ^]^

### In Vitro Cellular Uptake Experiment

To evaluate cellular uptake, KATO III and MKN‐45 cells were seeded in 6‐well plates at a density of 2 × 10^4^ cells per well and incubated for 24 h in complete DMEM. After 1 h of pre‐incubation, cells were treated with the following formulations in complete medium: (a) WSGC (600 µm), (b) WSGC@FA@PEG/PEI‐SPIONs (600 µm equiv. WSGC), (c) WSGC@FA@PEG/PEI‐SPIONs (600 µm equiv. WSGC) with free FA (0.1 g mL⁻^1^), and (d) WSGC@FA@PEG/PEI‐SPIONs+MF (600 µm equiv. WSGC+MF (MF: 0.15T)). After incubation, the cells were fixed with 4% paraformaldehyde, stained with Hoechst 33 258 (1 µg mL⁻^1^; 94 403, Sigma‐Aldrich, USA), and imaged using confocal laser scanning microscopy (CLSM; Leica TCS SP5, Germany).

Further confirmation of nanoparticle internalization was performed using transmission electron microscopy (TEM). KATO III and MKN‐45 cells were collected and fixed with 2.5% glutaraldehyde (8.20603, Sigma‐Aldrich, USA) in 0.1 m sodium bicarbonate buffer (S6014, Sigma‐Aldrich, USA) for 1 h. After rinsing with the same buffer, cells were post‐fixed with 1% osmium tetroxide (1.24505, Sigma‐Aldrich, USA) for 1 h. The samples were dehydrated through a graded ethanol series (50%, 70%, 95%, and 100%), and then infiltrated overnight at room temperature with a 1:1 mixture of acetone (179 124, Sigma‐Aldrich, USA) and Epon 812 resin (45 345, Sigma‐Aldrich, USA). After embedding in fresh Epon 812 resin, the samples were polymerized at 60 °C. Ultrathin sections (50–70 nm) were prepared using a Leica EM UC6 ultramicrotome (Leica, Großbeeren, Belgium) and transferred onto carbon‐coated copper grids. Sections were counterstained with 0.5% uranyl acetate (U302464, Aladdin) and lead citrate (L303843, Aladdin) prior to imaging.^[^ ^42^, ^43^ ^]^

### Transcriptome Sequencing and Data Analysis

Total RNA was extracted from tumor tissues of GA model mice (*n* = 3), treated or untreated with WSGC@FA@PEG/PEI‐SPIONs, using TRIzol reagent (Thermo, 16 096 020, USA). RNA samples were required to meet specific quality standards: RNA Integrity Number (RIN) ≥ 7.0 and 28S:18S ratio ≥ 1.5. RNA concentration and purity were assessed using the Qubit RNA Analysis Kit (Shanghai Baoji Biotechnology Co., Ltd., HKR2106‐01, CH) with a Qubit 2.0 Fluorometer (Life Technologies, Q33216, USA), and a Nanophotometer (IMPLEN, USA). Absorbance at 260 and 280 nm was measured to confirm that the A260/A280 ratio fell within the range of 1.8–2.0, indicating acceptable purity and minimal contamination.

Further analysis of RNA quality and integrity was performed using the RNA Nano 6000 Assay Kit (Agilent, 5067‐1511, USA) on a Bioanalyzer 2100 system (Agilent Technologies). Following the manufacturer's protocol, RNA samples were mixed with assay reagents and loaded onto a chip for electrophoretic separation, providing a precise assessment of RNA integrity and degradation. Only samples with high integrity and sufficient quantity (≥ 3 µg total RNA per sample) were used for library construction.

Complementary DNA (cDNA) libraries were prepared using the NEBNext Ultra RNA Library Prep Kit for Illumina (NEB, E7435L, CH), following the manufacturer's guidelines. Library quality was assessed using the Bioanalyzer 2100 system. Indexed libraries were then clustered on a cBot Cluster Generation System using the TruSeq PE Cluster Kit v3‐cBot‐HS (PE‐401‐3001, Illumina, USA), followed by sequencing on the Illumina HiSeq 550 platform to generate 125/150 bp paired‐end reads.

Raw sequencing reads were subjected to quality control using FastQC (v0.11.8). Preprocessing was performed with Cutadapt (v1.18) to trim Illumina adapter sequences and remove poly(A) tails. Reads containing more than 5% ambiguous bases (N content) were discarded using a custom Perl script. The FASTX Toolkit (v0.0.13) was applied to retain reads with a base quality score of ≥ 20 in at least 70% of positions. BBMap software was used to repair read pairs. The resulting high‐quality reads were aligned to the reference genome using HISAT2 (v0.7.12).

Differential expression analysis and enrichment analysis were conducted using the limma package in R. Differentially expressed genes (DEGs) were identified from both the sequencing data and TCGA dataset, using the criteria: |log₂ fold change| > 1 and *p*‐value < 0.05. Heatmaps and volcano plots of the DEGs were generated using R. Functional annotation, and pathway enrichment analyses were conducted using the ClusterProfiler package in R. Gene Ontology (GO) enrichment analysis included biological process (BP), molecular function (MF), and cellular component (CC) categories. KEGG pathway enrichment analysis was also performed. A threshold of *p* < 0.05 was used to determine statistical significance in enrichment results.

### Construction of Cisplatin‐Resistant GA Cell Lines

To establish cisplatin‐resistant KATO III and MKN‐45 cell lines, the protocol described by Coley was followed,^[^ ^44^ ^]^ utilizing cisplatin (CDDP; 232 120, Sigma, USA) for stepwise induction. Initially, drug sensitivity of the parental cells was determined, and the starting treatment concentration was set at 20% of the EC_50_ value. Cells were seeded according to their doubling time, and treatment commenced when the cells reached ≈20% confluency. Cisplatin was administered incrementally by doubling the concentration at each passage. This stepwise dose escalation was repeated for 30 passages to gradually develop drug tolerance. After the cells exhibited stable resistance, they were cultured in drug‐free medium for 1 month, cryopreserved in liquid nitrogen, and subsequently revived in CDDP‐containing medium to confirm their acquired resistance. The entire process of establishing drug‐resistant cell lines required ≈12 months.^[^ ^45^ ^]^

### Drug Sensitivity Assessment

Drug sensitivity was evaluated using the MTT assay. Parental cells (4 × 10^3^ cells/well) and cisplatin‐resistant cells (5.5 × 10^3^ cells/well) were seeded in 96‐well plates with 100 µL of culture medium per well and incubated for 24 h to allow attachment and reach ≈50% confluency. Cells were then treated with increasing concentrations of cisplatin (0.01 to 1 000 µm) for 72 h. Untreated cells served as the negative control.

Following treatment, culture medium was removed, and cells were washed with 100 µL of DPBS/Modified (14 190 144, Thermo Fisher). Then, 0.5 mg mL⁻^1^ MTT solution (M2128, Sigma) was added, and the plates were incubated in the dark for 2 h. Viable mitochondria in metabolically active cells reduced MTT to purple formazan crystals, which were solubilized using 100 µL of ethanol. Absorbance was measured at 570 nm using an Infinite NanoQuant spectrophotometer (TECAN, Switzerland). The half‐maximal effective concentration (EC_50_) was determined from dose‐response curves generated via nonlinear regression analysis. The Resistance Index (RI) was calculated by dividing the EC50 value of the drug‐resistant cell line by that of the parental cell line. Cells were considered cisplatin‐resistant when RI ≥ 2.

### Cell Death Assay

Cell death, including apoptosis, was evaluated using the Dead Cell Apoptosis Kit (A10788, ThermoFisher), containing Alexa Fluor 488 Annexin V (05‐184‐AF488) and propidium iodide (PI), according to the manufacturer's protocol. In brief, Parental cells (5 × 10^4^ cells/well) and drug‐resistant cells (7 × 10^4^ cells/well) were seeded in 6‐well plates containing 2 mL of culture medium per well and incubated for 24 h. Cells were then treated with cisplatin at the EC₅₀ concentration (as determined for the resistant cells) for 72 h. Following treatment, cells were harvested using trypsin (T4799, Sigma), centrifuged, and washed with 1 × PBS. The cell pellets were resuspended in 100 µL of 1 × annexin binding buffer.

For staining, 5 µL of Alexa Fluor 488 Annexin V and 1 µL of 100 µg mL⁻^1^ PI were added, and samples were incubated at 37 °C for 15 min in the dark. After staining, 400 µL of 1 × annexin buffer was added, and cells were analyzed immediately using a flow cytometer (FACSCanto II, BD, USA). As a positive control, 5% DMSO (34 943, Sigma) was used to induce apoptosis. Cells were classified as follows: Early apoptotic: Annexin V positive, PI negative; Late apoptotic: Annexin V positive, PI positive; Necrotic/dead: Annexin V negative, PI positive. Fluorescence signals were detected at excitation/emission maxima of 499/521 nm for Alexa Fluor 488 and 535/617 nm for PI.

### Lentivirus Construction

In this study, a lentiviral vector for overexpression was purchased: pCDH‐CMV‐MCS‐EF1α‐copGFP (overexpression vector, CD511B‐1, System Biosciences, USA), which was used to construct a lentivirus‐based overexpression vector for the Notch1 gene. The lentivirus particles carrying the vector were packaged into HEK‐293T cells (iCell‐h237, Cyagen Biosciences, Shanghai, China) using a lentivirus packaging kit (A35684CN, Invitrogen, USA). After 48 h, the cell culture supernatant was collected and filtered through a 0.45 µm membrane, and the lentivirus was harvested with a titer of 1 × 10^8^ TU/mL.

To establish GA cell lines overexpressing Notch1, human GA cells KATO III and MNK‐45 were cultured to ≈50% confluence. The cells were then infected with lentivirus at a concentration of 10^8^ IU/mL, following an MOI = 1. After 48 h post‐infection, cells were subjected to selection using 10 µg mL⁻^1^ puromycin (540 222, Sigma‐Aldrich, USA) and maintained for at least 1 week to allow for the selection of stably transfected cell lines.

### RT‐qPCR

Total RNA was extracted from cells using TRIzol reagent (Thermo Fisher Scientific, USA), and RNA concentration and purity were determined using a NanoDrop 2000 spectrophotometer (Thermo Fisher Scientific, USA). cDNA was synthesized from the extracted mRNA using the PrimeScript RT Reagent Kit (Takara, Japan; Code: RR047A), following the manufacturer's protocol. Gene‐specific primers were designed and synthesized by TaKaRa. Quantitative real‐time PCR (RT‐qPCR) was performed using the 7500 Fast Real‐Time PCR System (Thermo Fisher Scientific, USA). The thermal cycling conditions were as follows: initial denaturation at 95 °C for 10 min, followed by 40 cycles of denaturation at 95 °C for 10 s, annealing at 60 °C for 20 s, and extension at 72 °C for 34 s.

The primer sequences for the molecular markers involved in CDDP resistance (genes ABCC2, ATP7A, and CTR1) were as follows: (1) ABCC2‐F: GCCAACTTGTGGCTGTGATAGG, ABCC2‐R: ATCCAGGACTGCTGTGGGACAT; (2) ATP7A‐F: AAACTGCAAGGTGTTCAGCG, ATP7A‐R: AGCCCATAGCTTCAATCTGCT; (3) CTR1‐F: ACCATCACCCAACCACTTCA, CTR1‐R: CCGGAAAACAGTAGTTCCACA; (4) NOTCH1‐F: 5′‐GACATCACGGATCATATGGA‐3′, NOTCH1‐R: 5′‐CTCGCATTGACCATTCAAAC‐3′; (5) HEY1‐F: 5′‐CCGAGTACAGCTCCTCGGACA‐3′, HEY1‐R: 5′‐CGATCTCCGTCCCCCAAGGTC‐3′; (6) HES1‐F: 5′‐CTGATATAATGGAGAAAAATT‐3′, HES1‐R: 5′‐CCGCCACGGCCTCCACATGGA‐3′. The following primers were used for FRα detection: Human FRα‐F: 5′‐CCAGCAGGTGGATCAGAGCTG‐3′; Human FRα‐R: 5′‐CGACCATGGAGCAGGAACC‐3′; Mouse FRα‐F: 5′‐GAAGACGAATTCCTGCTGT‐3′; Mouse FRα‐R: 5′‐TGAGCTTGTAGGAGTGACT‐3′. The internal control primer used was β‐actin, with sequences such as β‐actin‐F: ATCATTGCTCCTCCTGAGC and β‐actin‐R: ACTCCTGCTTGCTGATCCAC. Relative gene expression levels were calculated using the comparative 2^−ΔΔCT^ method. Each experiment was performed in triplicate.

### Western Blot

Total protein was extracted from cells or tissues using RIPA lysis buffer supplemented with PMSF (P0013C, Beyotime, Shanghai, China). Samples were incubated on ice for 30 min and centrifuged at 8 000 × g for 10 min at 4 °C. The supernatant containing total protein was collected, and protein concentration was quantified using the BCA Protein Assay Kit (23 227, Thermo Fisher Scientific, USA).

For protein analysis, 50 µg of total protein was mixed with 2 × SDS loading buffer, boiled at 100 °C for 5 min, and separated by SDS‐PAGE. The proteins were then transferred to a PVDF membrane, which was blocked in 5% non‐fat dry milk for 1 h at room temperature. The membrane was incubated overnight at 4 °C with the following primary antibodies: Snail (1:1500, ab216347), E‐cadherin (1:1500, ab40772), N‐cadherin (1:1500, ab76011), Atg5 (1:1000, ab108327), LC3B (1:1000, ab192890), p62 (1:1000, ab109012), Beclin1 (1:1500, ab302669), PINK1 (1:1000, ab216144), Parkin (1:1000, ab77924), β‐actin (1:1000, ab8226, loading control for total protein), and VDAC (ab14734, 1:1000, internal control for mitochondrial proteins).

After three washes with TBST (10 min each), the membrane was incubated with an HRP‐conjugated goat anti‐rabbit IgG secondary antibody (1:2000, ab97051, Abcam, UK) for 1 h at room temperature. Protein bands were visualized using an ECL chemiluminescence detection kit (abs920, Absin Bio‐Tech Co., Ltd., Shanghai, China). Signals were captured using the Bio‐Rad imaging system, and band intensities were analyzed using Quantity One v4.6.2 software. Protein expression was quantified as the grayscale intensity ratio of the target protein to β‐actin. Each experiment was independently repeated three times, and the mean values were calculated.

### MTT

Cell proliferation and viability were assessed using the MTT Cell Proliferation and Cytotoxicity Assay Kit (C0009S, Beyotime, China). Cells were cultured in 96‐well plates until reaching the desired density. According to the manufacturer's instructions, MTT reagent was added to each well and incubated at 37 °C for 2–4 h. After incubation, the formazan crystals formed by viable cells were solubilized, and absorbance was measured at 570 nm using a microplate reader. Cell viability and proliferation rates were calculated based on the absorbance values.

### Wound Healing Assay

Cells in optimal growth condition were detached and resuspended to prepare a single‐cell suspension. Cells were seeded into six‐well plates at a density of 8 × 10^5^ cells/mL in 2 mL of medium per well. After reaching 90%–100% confluence, a sterile 200 µL pipette tip was used to create two parallel scratch wounds across the monolayer. The wells were gently rinsed twice with PBS to remove cell debris, and serum‐free RPMI 1640 medium was added. Cells were incubated in a CO_2_‐regulated incubator at 37 °C for 24 h. Images of the scratch area were captured at 0 and 24 h, and the wound healing rate was calculated to assess cell migration.

### Clonogenic Assay

To determine the long‐term proliferative potential of cells, a clonogenic assay was performed. Cells were seeded into 6 cm culture dishes at a density of 2000 cells per dish and cultured in complete medium. The medium was refreshed periodically, and cells were allowed to grow undisturbed for 14 days to form colonies. At the end of the incubation period, colonies were fixed and stained with 0.5% (w/v) Crystal Violet (C8470, Beijing Solarbio Technology Co., Ltd., Beijing, China) for visualization. Colonies consisting of more than 50 cells, as observed under a microscope, were counted as individual clones.

### Transwell Migration Assay

For the invasion assay, Matrigel (catalog number: 356 234, Shanghai Haoyang Biotechnology Co., Ltd., Shanghai, China) was thawed at 4 °C overnight. The gel was diluted by mixing 200 µL of liquefied Matrigel with 200 µL of serum‐free medium under cold conditions. Subsequently, 50 µL of this diluted matrix was added to the upper chambers of Transwell inserts and incubated at 37 °C for 2–3 h to solidify. Cells were harvested, counted, and resuspended in serum‐free medium. A 200 µL volume of the cell suspension was seeded into the upper chamber of each insert, while 800 µL of culture medium supplemented with 20% FBS was added to the lower chamber as a chemoattractant. Plates were incubated at 37 °C for 24 h. After incubation, cells on the upper surface of the membrane were carefully removed with a cotton swab. The remaining cells on the lower surface were fixed in formaldehyde for 10 min, rinsed three times with distilled water, and stained with 0.1% Crystal Violet for 30 min at room temperature. The inserts were gently washed with PBS, and migrated or invaded cells were observed and photographed under an inverted microscope (DMi8, Leica). Cell counting was performed in at least four randomly selected microscopic fields per insert. Each experiment was repeated three times. For the migration assay, the procedure was the same as above, except that the Transwell chambers were not coated with Matrigel. Cells were incubated for 24 h under the same conditions.

### Flow Cytometry Analysis

Flow cytometry was employed to analyze cell cycle distribution. After harvesting and processing as previously described, cells were fixed in 70% cold ethanol and stored overnight at 4 °C. The next day, cells were centrifuged at 800 × g to remove ethanol and resuspended in a binding buffer containing RNase A (10 µg mL⁻^1^) for 30 min at 37 °C to remove residual RNA. Following RNase treatment, cells were stained with 50 µL of PI solution (50 mg L⁻^1^, 40710ES03, YEASEN, Shanghai) for 30 min in the dark. Flow cytometry analysis was then conducted using a BD LSRFortessa flow cytometer (BD BioPAADence, USA) to determine the distribution of cells across different phases of the cell cycle.

### In Vivo Pharmacological and Toxicological Evaluation

All animal procedures were conducted in accordance with the guidelines of the Institutional Animal Care and Use Committee (IACUC). Female BALB/c nude mice (strain code: 401, Vital River), weighing 18–20 g, were subcutaneously inoculated with 5 × 10^6^ human GA cells in the left axillary region to establish tumor‐bearing models. Tumor volume was monitored, and when it reached ≈50 mm^3^ (after ≈15 days), mice were randomly assigned to experimental groups.

WSGC dose screening: To determine the optimal therapeutic dose of the WSGC peptide, mice were divided into four groups (*n* = 5 per group): Group 1 (Control): received saline injection as a control; Group 2 (20 nmol g⁻^1^ WSGC): received WSGC tail vein injection at a dosage of 20 nmol g⁻^1^; Group 3 (40 nmol g⁻^1^ WSGC): received WSGC tail vein injection at a dosage of 40 nmol g⁻^1^; and Group 4 (80  nmol g⁻^1^ WSGC): received WSGC tail vein injection at a dosage of 80 nmol g⁻^1^. The dosage was administered every 2 days for a total of 21 days.

After the preliminary dosage screening of WSGC, the animal grouping was further updated into the following four groups (*n* = 5): Group 1 (Control): received saline injection as the control; Group 2 (WSGC): received WSGC tail vein injection (administered based on the dose of 80 nmol g⁻^1^ polypeptide units); Group 3 (WSGC@FA@PEG/PEI‐SPIONs): received WSGC@FA@PEG/PEI‐SPIONs tail vein injection; Group 4 (WSGC@FA@PEG/PEI‐SPIONs+ MF): received WSGC@FA@PEG/PEI‐SPIONs tail vein injection followed immediately by the placement of a 0.15 T Nb‐Fe‐B disc‐shaped magnet (diameter 10 mm × thickness 2 mm) on the tumor for 2 h. The dosage of WSGC was 80 nmol g⁻^1^ (equiv. to the WSGC dosage in WSGC@FA@PEG/PEI‐SPIONs), administered via tail vein injection twice a day for 21 days. Tumor dimensions—length (a) and width (b)—were measured every 3 days using calipers. Tumor volume was calculated using the formula: v = a × b^2^/2. At the end of the study (day 36), mice were euthanized, and tumor tissues along with heart, liver, lungs, and kidneys were collected. Hematoxylin and eosin (H&E) staining was performed on these tissues to evaluate biocompatibility and systemic toxicity under a light microscope.^[^ ^46^ ^]^

To investigate the mechanism of WSGC peptide in modulating mitophagy through the Notch signaling pathway, additional in vivo experiments were conducted. BALB/c nude mice were subcutaneously inoculated with either cisplatin‐resistant KATO III cells (KATO III R‐CDDP) or KATO III R‐CDDP cells stably overexpressing Notch1 (2 × 10^6^ cells per mouse). Mice were divided into five groups (*n* = 5 each): Normal group: healthy mice injected with an equal volume of normal saline; Control group: injection of drug‐resistant GA cells treated with an equal volume of saline; Treat group: injection of drug‐resistant GA cells with WSGC@FA@PEG/PEI‐SPIONs (80 mg kg⁻^1^ equiv. to WSGC peptide), administered thrice weekly for 4 weeks; Treat+oe‐NOTCH1 group: injection of drug‐resistant GA cells overexpressing Notch1, treated with WSGC peptide; Treat+oe‐NOTCH1+Chloroquine group: injection of drug‐resistant GA cells overexpressing Notch1, treated with WSGC@FA@PEG/PEI‐SPIONs (80 mg kg⁻^1^ equiv. to WSGC peptide) and Chloroquine (80 mg kg⁻^1^, iv) (C6628, Sigma) in combination. Tumor volume was recorded every 3 days. On day 30, final tumor volume and weight were measured, and tumor tissues were harvested for further molecular and histological analyses.^[^ ^47^, ^48^ ^]^ Blood samples were collected on day 30 via submandibular bleeding. For complete blood count (CBC), samples were collected into Microvette 100 K2‐EDTA tubes (Sarstedt). For serum biochemical analysis, blood was collected in anticoagulant‐free tubes, allowed to clot for at least 30 min, and centrifuged at 3000 × g for 10 min to obtain the serum.

### Biocompatibility of WSGC@FA@PEG/PEI‐SPIONs in Mice

To evaluate biodistribution and biocompatibility, mice from the WSGC@FA@PEG/PEI‐SPIONs group and the WSGC@FA@PEG/PEI‐SPIONs + MF group (*n* = 5 per group) were treated via tail vein injection. After 2 h, tumor, liver, lung, and spleen tissues were collected, dried at 105 °C to constant weight, and subjected to acid digestion. Specifically, 1 mL of concentrated nitric acid and 0.2 mL of concentrated hydrochloric acid were added to each sample, followed by heating at 80 °C for 2 h. Then, 0.1 mL of Triton X‐100 (T8787, Sigma‐Aldrich, USA) was added, and the mixture was incubated for an additional hour. The digested samples were diluted to a final volume of 50 mL. Iron concentration in each tissue was then measured using Inductively Coupled Plasma Optical Emission Spectrometry (ICP‐OES).

### Evaluation of Magnetic Targeting Efficiency

To assess the magnetic targeting capability of WSGC@FA@PEG/PEI‐SPIONs, mice were anesthetized with 1.5% isoflurane (PHR2874, Sigma‐Aldrich, USA) in oxygen. A disc‐shaped neodymium magnet (0.15 T, 10 mm diameter × 2 mm thickness) was fixed to the tumor site. Mice were administered WSGC@FA@PEG/PEI‐SPIONs via tail vein injection (20 mg Fe/kg body weight) and retained under the MF for 2 h. A control group received the same nanoparticle dosage without magnetic targeting. MRI was performed 12 h post‐injection using a Turbo‐Spin Echo T2‐weighted sequence with the following parameters: TR/TE = 2 300/110 ms, NEX = 2, matrix size = 256 × 256, field of view = 56 mm × 70 mm, slice thickness = 2.4 mm, and slice gap = 2.9 mm. After imaging, tumor tissues were harvested, sectioned, and stained with Prussian Blue to visualize iron accumulation, followed by light microscopy examination.

### Immunohistochemical Staining to Detect Protein Expression in Tissues/Cells

Tissue and cell samples were fixed in 4% paraformaldehyde overnight and embedded in paraffin. Sections (4 µm thick) were deparaffinized in xylene and rehydrated through graded ethanol (100%, 95%, 75%; 3 min each). Antigen retrieval was performed by boiling the sections in 0.01 m citrate buffer (C9999, Sigma, USA) for 15–20 min. Endogenous peroxidase activity was quenched with 3% H_2_O_2_ (88 597, Sigma, USA) for 30 min at room temperature. After rinsing, non‐specific binding was blocked with goat serum for 20 min. Excess serum was removed, and the sections were incubated with primary antibodies at room temperature for 1 h. The following antibodies were used (all from Abcam, 1:200 dilution): Ki67 (ab16667), Bcl‐2 (ab182858), Bax (ab32503), Cleaved Caspase‐3 (ab32042), and PCNA (ab29). Following primary antibody incubation, slides were washed with PBS and incubated with HRP‐conjugated IgG secondary antibody (ab6785, 1:1000, Abcam) at 37 °C for 20 min. After washing, sections were treated with SP (avidin‐biotin‐peroxidase complex) for 30 min, then visualized using DAB substrate (P0202, Beyotime Biotechnology Co., Ltd.) for 5–10 min. The reaction was terminated by rinsing in distilled water. Counterstaining was performed with hematoxylin (C0107, Beyotime), followed by differentiation in hydrochloric acid ethanol, rinsing, dehydration through graded ethanol, clearing in xylene, and sealing with neutral resin. Protein expression was observed and quantified using light microscopy.

### Detection of JC‐1

Mitochondrial membrane potential (MMP) was assessed using the Mitochondrial Membrane Potential Assay Kit (Abcam, ab113850), following the manufacturer's protocol. In this assay, healthy mitochondria exhibit red fluorescence due to JC‐1 dye aggregation (J‐aggregates), while mitochondria with depolarized membrane potential emit green fluorescence due to JC‐1 monomers.

### H&E Staining

Liver tissue sections were subjected to H&E staining using a commercially available staining kit (PT001, Shanghai Bogu Biotechnology Co., Ltd., China) according to the manufacturer's instructions. Briefly, paraffin‐embedded tissue sections were deparaffinized in xylene and rehydrated through a graded ethanol series (100%, 95%, 80%, and 70%). Sections were then stained with hematoxylin for 10 min at room temperature, rinsed under running water for 30–60 s, and differentiated in 1% hydrochloric acid ethanol for 30 s. After an additional rinse and 5‐min soak in running water, the sections were counterstained with eosin for 1 min. Dehydration was performed through a graded ethanol series, followed by clearing in xylene. Finally, the sections were mounted with neutral resin and examined under a light microscope (BX50, Olympus Corp., Tokyo, Japan) to assess histopathological changes.

### Prussian Blue Staining

Tumor tissues were fixed in 4% formaldehyde (1.00496, Sigma‐Aldrich, USA), then dehydrated sequentially in ethanol (70%, 80%, 95%, and 100%) for 30 min at each concentration, followed by clearing in xylene (247 642, Sigma‐Aldrich, USA) twice for 30 min each. Tissues were then embedded in paraffin and sectioned into 4–5 µm slices using a microtome. After mounting and drying, tissue sections were rehydrated through descending ethanol concentrations (100% to 70%, 5 min each). Sections were then stained in a Prussian Blue solution, prepared by mixing equal volumes of 2% potassium hydroxide (60377, Sigma‐Aldrich) and 2% potassium ferricyanide(III) (702587, Sigma‐Aldrich), for 20–30 min. After rinsing in distilled water, nuclei were counterstained with Nuclear Fast Red (60 700, Sigma‐Aldrich) for 5 min. Following nuclear staining, sections were dehydrated in graded ethanol, cleared in xylene (twice for 5 min), mounted with coverslips, and observed under an optical microscope to evaluate iron accumulation.

### Measurement of Reactive Oxygen Species (ROS)

Intracellular ROS levels were measured using the redox‐sensitive fluorescent probe CM‐H₂DCFDA (C6827, Invitrogen, USA). Cells were incubated with the probe at 37 °C for 10 min, as per the manufacturer's instructions. ROS levels were then quantified by measuring fluorescence intensity using a microplate reader or fluorescence microscope.

### Measurement of ATP and NAD^+^/NADH Content

Cellular ATP content was quantified using an ATP Assay Kit (S0026, Beyotime, China), following the manufacturer's instructions. Simultaneously, the NAD⁺/NADH ratio was determined using a colorimetric NAD^+^/NADH Assay Kit (WST‐8 method; S0175, Beyotime, China).

### Caspase‐3 Activity Assay

Caspase‐3 enzymatic activity was measured using a Caspase‐3 Activity Assay Kit (C1115, Beyotime, China) according to the manufacturer's protocol. Briefly, 3–10 mg of tissue was homogenized in 100 µL of lysis buffer using a glass homogenizer on ice. The homogenate was transferred to a 1.5 mL microcentrifuge tube and further lysed on ice for 5 min. The samples were then centrifuged at 16 000–20 000 × g for 10–15 min at 4 °C. The supernatant was collected into pre‐chilled tubes and immediately used for the detection of caspase‐3 activity by measuring absorbance at 405 nm using a microplate reader.

### Statistical Analysis

All data are presented as mean ± standard deviation (mean ± SD) from at least three independent experiments. For comparisons between two groups, an independent two‐tailed Student's *t*‐test was used. For comparisons among three or more groups, one‐way analysis of variance (ANOVA) was applied. If the ANOVA indicated significant differences, Tukey's Honestly Significant Difference (HSD) post hoc test was conducted for pairwise comparisons. For data that did not meet the assumptions of normality or homogeneity of variance, the Mann‐Whitney U test or Kruskal‐Wallis H test was used as appropriate. A *p*‐value of < 0.05 was considered statistically significant. Cell‐based assays were conducted in triplicate (*n* = 3), and animal studies included five mice per group (*n* = 5). All statistical analyses were performed using GraphPad Prism version 9.0 (GraphPad Software, USA).

### Ethics Approval and Consent to Participate

All animal experiments were approved by the Animal Ethics Committee of the First Affiliated Hospital of Zhengzhou University (Approval No. 2019‐KY‐200).

## Conflict of Interest

The authors declare no conflict of interest.

## Author Contributions

D.S., Q.L., Z.Y., and Q.W. are regarded as co‐first authors. D.S., Q.L., Z.Y., and Q.W. conceived and designed the study. M.S., H.Z., L.S., Y.F., Q.Z., H.Y., and D.Z. performed the experiments. M.S., H.Z., L.S., Y.F., and Q.Z. analyzed the data. Q.L., Z.Y., and Q.W. wrote the manuscript. D.S. supervised the study. All authors reviewed and approved the final version of the manuscript.

## Supporting information



Supporting Information

Supporting Information

## Data Availability

The data that support the findings of this study are available from the corresponding author upon reasonable request.

## References

[1] L. Liu , X. Li , L. Song , Y. Yang , B. Li , Pathol., Res. Pract. 2024, 257, 155325. 38678850 10.1016/j.prp.2024.155325

[2] X. Li , H. Zhang , Y. Cui , H. Zhang , Y. Wang , M. Ding , X. Zhu , R. Zhang , Q. Hu , L. Tao , et al., Diagn Pathol 2023, 18, 35. 36871023 10.1186/s13000-023-01311-1 PMC9985287

[3] T. Hess , C. Maj , J. Gehlen , O. Borisov , S. L. Haas , I. Gockel , M. Vieth , G. Piessen , H. Alakus , Y. Vashist , C. Pereira , M. Knapp , V. Schüller , A. Quaas , H. I. Grabsch , J. Trautmann , E. Malecka‐Wojciesko , A. Mokrowiecka , J. Speller , A. Mayr , J. Schröder , A. M. Hillmer , D. Heider , F. Lordick , Á. Pérez‐Aísa , R. Campo , J. Espinel , F. Geijo , C. Thomson , L. Bujanda , et al., eBioMedicine 2023, 92, 104616. 37209533 10.1016/j.ebiom.2023.104616 PMC10212786

[4] A. van der Veen , H. J. F. Brenkman , M. F. J. Seesing , L. Haverkamp , M. D. P. Luyer , G. A. P. Nieuwenhuijzen , J. H. M. B. Stoot , J. J. W. Tegels , B. P. L. Wijnhoven , S. M. Lagarde , W. O. de Steur , H. H. Hartgrink , E. A. Kouwenhoven , E. B. Wassenaar , W. A. Draaisma , S. S. Gisbertz , D. L. van der Peet , A. M. May , J. P. Ruurda , R. van Hillegersberg , A. M. Eligh , J. E. H. Ponten , F. F. B. M. Heesakkers , K. W. E. Hulsewe , T. T. T. Tweed , J. J. B. van Lanschot , M. J. van Det , P. van Duijvendijk , E. S. van der Zaag , I. A. M. J. Broeders , et al., JCO 2021, 39, 978. 10.1200/JCO.20.01540 34581617

[5] B. Wu , L. Fu , X. Guo , H. Hu , Y. Li , Y. Shi , Y. Zhang , S. Han , C. Lv , Y. Tian , Front. Immunol. 2023, 14, 984816. 36761750 10.3389/fimmu.2023.984816 PMC9905807

[6] R. J. Kelly , J. A. Ajani , J. Kuzdzal , T. Zander , E. Van Cutsem , G. Piessen , G. Mendez , J. Feliciano , S. Motoyama , A. Lièvre , H. Uronis , E. Elimova , C. Grootscholten , K. Geboes , S. Zafar , S. Snow , A. H. Ko , K. Feeney , M. Schenker , P. Kocon , J. Zhang , L. Zhu , M. Lei , P. Singh , K. Kondo , J. M. Cleary , M. Moehler , N. Engl. J. Med. 2021, 384, 1191. 33789008 10.1056/NEJMoa2032125

[7] R. Nustas , A. A. Messallam , T. Gillespie , S. Keilin , S. Chawla , V. Patel , Q. Cai , F. F. Willingham , Surg. Endosc. 2022, 36, 3876. 34463872 10.1007/s00464-021-08704-7

[8] V. Dabout , C. de la Fouchardière , T. Voron , T. André , F. Huguet , R. Cohen , Bull. Cancer 2023, 110, 521. 35965103 10.1016/j.bulcan.2022.05.014

[9] F. P. Mesquita , P. F. N. Souza , E. L. da Silva , L. B. Lima , L. L. B. de Oliveira , C. A. Moreira‐Nunes , W. J. Zuercher , R. M. R. Burbano , M. E. A. de Moraes , R. C. Montenegro , Pharmaceutics 2022, 14, 1841. 36145589 10.3390/pharmaceutics14091841 PMC9501214

[10] Y. Watanabe , M. Shiobara , K. Wakatsuki , K. Suda , K. Miyazawa , T. Miyoshi , T. Aida , N. Yoneura , S. Yoshioka , K. Yamazaki , Gan to Kagaku Ryoho 2020, 47, 2192. 33468904

[11] Y. Sun , W. Shen , S. Hu , Q. Lyu , Q. Wang , T. Wei , W. Zhu , J. Zhang , J Exp Clin Cancer Res 2023, 42, 65. 36932427 10.1186/s13046-023-02638-9 PMC10022264

[12] K. Zeng , W. Li , Y. Wang , Z. Zhang , L. Zhang , W. Zhang , Y. Xing , C. Zhou , Adv. Sci. 2023, 10, 2301088. 10.1002/advs.202301088 PMC10477855 37428466

[13] Q. Kong , S. Xia , X. Pan , K. Ye , Z. Li , H. Li , X. Tang , N. Sahni , S. S. Yi , X. Liu , H. Wu , M. B. Elowitz , J. Lieberman , Z. Zhang , Sci. Immunol. 2023, 8, adg3196. 10.1126/sciimmunol.adg3196 PMC10338320 37115914

[14] Y.a‐X. Zhu , H.‐R. Jia , G.e Gao , G.‐Y.u Pan , Y.‐W. Jiang , P. Li , N. Zhou , C. Li , C. She , N. W. Ulrich , Z. Chen , F.u‐G. Wu , Biomaterials 2020, 232, 119668. 31927179 10.1016/j.biomaterials.2019.119668

[15] J. D. Yu , S. Miyamoto , Cells 2021, 10, 3330. 34943839 10.3390/cells10123330 PMC8699551

[16] Z. Zeng , X. Zhou , Y. Wang , H. Cao , J. Guo , P. Wang , Y. Yang , Y. Wang , Biomolecules 2022, 12, 1420. 36291629 10.3390/biom12101420 PMC9599755

[17] M. Giansanti , T. Theinert , S. K. Boeing , D. Haas , P.‐G. Schlegel , P. Vacca , F. Nazio , I. Caruana , Mol. Cancer 2023, 22, 201. 38071322 10.1186/s12943-023-01893-w PMC10709869

[18] M. Shen , D. Wang , Y. Sennari , Z. Zeng , R. Baba , H. Morimoto , N. Kitamura , T. Nakanishi , J. Tsukada , M. Ueno , et al., Med Oncol 2022, 39, 118. 35674939 10.1007/s12032-022-01707-x

[19] J. Xiang , C. Zhang , T. Di , L. Chen , W. Zhao , L. Wei , S. Zhou , X. Wu , G. Wang , Y. Zhang , Bioengineered 2022, 13, 3486. 35068334 10.1080/21655979.2022.2026552 PMC8974099

[20] X. Li , C. Wang , J. Zhu , Q. Lin , M. Yu , J. Wen , J. Feng , C. Hu , Oxid. Med. Cell. Longev. 2022, 2022, 3745135. 35132348 10.1155/2022/3745135 PMC8817854

[21] M. Liu , Y. Fan , D. Li , B. Han , Y. Meng , F. Chen , T. Liu , Z. Song , Y.u Han , L. Huang , Y. Chang , P. Cao , A. Nakai , K.e Tan , Mol. Oncol. 2021, 15, 2084. 33675143 10.1002/1878-0261.12936 PMC8334255

[22] L. Garcia‐Alonso , L.‐F. Handfield , K. Roberts , K. Nikolakopoulou , R. C. Fernando , L. Gardner , B. Woodhams , A. Arutyunyan , K. Polanski , R. Hoo , C. Sancho‐Serra , T. Li , K. Kwakwa , E. Tuck , V. Lorenzi , H. Massalha , M. Prete , V. Kleshchevnikov , A. Tarkowska , T. Porter , C. I. Mazzeo , S. van Dongen , M. Dabrowska , V. Vaskivskyi , K. T. Mahbubani , J.‐E. Park , M. Jimenez‐Linan , L. Campos , V. Y.u. Kiselev , C. Lindskog , et al., Nat. Genet. 2021, 53, 1698. 34857954 10.1038/s41588-021-00972-2 PMC8648563

[23] K. Liu , X. Meng , Z. Liu , M. Tang , Z. Lv , X. Huang , H. Jin , X. Han , X. Liu , W. Pu , H. Zhu , B. Zhou , Cell 2024, 187, 2428. 38579712 10.1016/j.cell.2024.03.010

[24] O. Gozlan , D. Sprinzak , Development 2023, 150, dev201138. 36794955 10.1242/dev.201138

[25] L. Chen , H. Lu , D. Peng , L. L. Cao , F. Ballout , K. Srirmajayam , Z. Chen , A. Bhat , T. C. Wang , A. Capobianco , J. Que , O. G. McDonald , A. Zaika , S. Zhang , W. El‐Rifai , Gut 2022, 72, 421. 35750470 10.1136/gutjnl-2022-327076 PMC9789198

[26] K. Maslenkina , L. Mikhaleva , M. Naumenko , R. Vandysheva , M. Gushchin , D. Atiakshin , I. Buchwalow , M. Tiemann , Int. J. Mol. Sci. 2023, 24, 9304. 37298253 10.3390/ijms24119304 PMC10253447

[27] T. Seidlitz , T. Schmäche , F. Garc?a , J. H.o Lee , N. Qin , S. Kochall , J. Fohgrub , D. Pauck , A. Rothe , B.‐K. Koo , J. Weitz , M. Remke , J. Muñoz , D. E. Stange , EMBO Mol. Med. 2022, 14, 15705. 10.15252/emmm.202215705 PMC9549728 35993110

[28] G. Yoshida , T. Kawabata , H. Takamatsu , S. Saita , S. Nakamura , K. Nishikawa , M. Fujiwara , Y. Enokidani , T. Yamamuro , K. Tabata , M. Hamasaki , M. Ishii , A. Kumanogoh , T. Yoshimori , Autophagy 2022, 18, 2323. 35025696 10.1080/15548627.2021.2017587 PMC9542956

[29] V. N. Huynh , S. Wang , X. Ouyang , W. Y. Wani , M. S. Johnson , B. K. Chacko , A. G. Jegga , W.‐J. Qian , J. C. Chatham , V. M. Darley‐Usmar , J. Zhang , Front. Aging 2021, 2, 757801. 35822049 10.3389/fragi.2021.757801 PMC9261315

[30] J. Xiang , W. Gong , J. Liu , H. Zhang , M. Li , R. Wang , Y. Lv , P. Sun , EMBO Mol Med. 2023, 14, 1098190. 10.3389/fgene.2023.1098190 PMC10233108 37274780

[31] K. Zhao , Y. Wu , D. Zhao , H. Zhang , J. Lin , Y. Wang , Front. Neurosci. 2023, 17, 1125281. 37274215 10.3389/fnins.2023.1125281 PMC10232817

[32] L. Zhu , H. Mao , L. Yang , WIREs Nanomed. Nanobiotechnol. 2022, 14, 1793. 10.1002/wnan.1793 PMC9373845 35396932

[33] X. Feng , Y. Xue , S. Gonca , K. Ji , M. Zhang , F. R. García‐García , Q. Li , Y. Huang , K. V. Kamenev , X. Chen , J. Mater. Chem. B 2023, 11, 3422. 37000531 10.1039/d2tb02630a PMC10091360

[34] W. Jiao , T. Zhang , M. Peng , J. Yi , Y. He , H. Fan , Biosensors 2022, 12, 38. 35049666 10.3390/bios12010038 PMC8774163

[35] M. Rahman , Nanotheranostics 2023, 7, 424. 37650011 10.7150/ntno.86467 PMC10464520

[36] B. Zhou , J. Liu , L. Wang , M. Wang , C. Zhao , H. Lin , Y. Liang , R. A. Towner , W. R. Chen , Nanoscale 2022, 14, 4588. 35253815 10.1039/d1nr07750c PMC9001247

[37] R. Baghban , M. Afarid , J. Soleymani , M. Rahimi , Biomed. Pharmacother. 2021, 144, 112321. 34656061 10.1016/j.biopha.2021.112321

[38] Z. Guo , R. Xing , M. Zhao , Y. Li , H. Lu , Z. Liu , Adv. Sci. 2021, 8, 2101713. 10.1002/advs.202101713 PMC8693047 34725943

[39] L. Lu , L. Jie , Y. Zhou , J. Zhang , T. Feng , Y. Zhu , T. Chen , X. Zhu , J. Ji , Z. Wang , Curr. Pharm. Des. 2023, 29, 686. 36967466 10.2174/1381612829666230324091555

[40] A. Baghbanzadeh , E. Baghbani , K. Hajiasgharzadeh , S. Noorolyai , V. Khaze , B. Mansoori , M. Shirmohamadi , B. Baradaran , A. Mokhtarzadeh , Adv. Pharm. Bull. 2022, 12, 169. 35517889 10.34172/apb.2022.018 PMC9012914

[41] Y. Luo , X. Yu , P. Zhao , J. Huang , X. Huang , Inflammation 2022, 45, 2449. 35705831 10.1007/s10753-022-01704-2

[42] Y. Huang , K. Mao , B. Zhang , Y. Zhao , Mater. Sci. Eng., C 2017, 70, 763. 10.1016/j.msec.2016.09.052 27770953

[43] G. Yang , W. Ma , B. Zhang , Q. Xie , Mater. Sci. Eng., C 2016, 62, 384. 10.1016/j.msec.2016.01.090 26952437

[44] H. M. Coley , Cancer Cell Culture, 267.

[45] B. Mora‐Lagos , I. Cartas‐Espinel , I. Riquelme , A. C. Parker , S. R. Piccolo , T. Viscarra , M. E. Reyes , L. Zanella , K. Buchegger , C. Ili , P. Brebi , PLoS One 2020, 15, 0228331. 10.1371/journal.pone.0228331 PMC6986722 31990955

[46] J. A. Ajani , Y. Xu , L. Huo , R. Wang , Y. Li , Y. Wang , M. P. Pizzi , A. Scott , K. Harada , L. Ma , X. Yao , J. Jin , W. Zhao , X. Dong , B. D. Badgwell , N. Shanbhag , G. Tatlonghari , J. S. Estrella , S. Roy‐Chowdhuri , M. Kobayashi , J. V. Vykoukal , S. M. Hanash , G. A. Calin , G. Peng , J.u‐S. Lee , R. L. Johnson , Z. Wang , L. Wang , S. Song , Gut 2020, 70, 55. 32345613 10.1136/gutjnl-2019-319748 PMC9832914

[47] S. Song , J. A. Ajani , S. Honjo , D. M. Maru , Q. Chen , A. W. Scott , T. R. Heallen , L. Xiao , W. L. Hofstetter , B. Weston , J. H. Lee , R. Wadhwa , K. Sudo , J. R. Stroehlein , J. F. Martin , M.‐C. Hung , R. L. Johnson , Cancer Res. 2014, 74, 4170. 24906622 10.1158/0008-5472.CAN-13-3569 PMC4136429

[48] F. E. Mohamed , R. M. Al‐Jehani , S. S. Minogue , F. Andreola , A. Winstanley , S. W. M. Olde Damink , A. Habtesion , M. Malagó , N. Davies , T.u V. Luong , A. P. Dhillon , R. P. Mookerjee , D. K. Dhar , R. Jalan , Liver Int. 2014, 35, 1063. 24990399 10.1111/liv.12626

[49] X. Li , Y. Qian , T. Liu , X. Hu , G. Zhang , Y. You , S. Liu , Biomaterials 2011, 32, 6595. 21663960 10.1016/j.biomaterials.2011.05.049

[50] S. Laurent , A. A. Saei , S. Behzadi , A. Panahifar , M. Mahmoudi , Expert Opin. Drug Delivery 2014, 11, 1449. 10.1517/17425247.2014.924501 24870351

[51] D. Song , L. Yue , H. Li , J. Zhang , Z. Yan , Y. Fan , H. Yang , Q. Liu , D.a Zhang , Z. Xia , P. Qin , J. Jia , M. Yue , J. Yu , S. Zheng , F. Yang , J. Wang , Br. J. Cancer 2016, 114, 929. 27002935 10.1038/bjc.2016.52 PMC4984799

[52] M. J. Mitchell , M. M. Billingsley , R. M. Haley , M. E. Wechsler , N. A. Peppas , R. Langer , Nat. Rev. Drug Discovery 2020, 20, 101. 33277608 10.1038/s41573-020-0090-8 PMC7717100

[53] J. O. Eloy , R. Petrilli , J. F. Topan , H. M. R. Antonio , J. P. A. Barcellos , D. L. Chesca , L. N. Serafini , D. G. Tiezzi , R. J. Lee , J. M. Marchetti , Colloids Surf., B 2016, 141, 74. 10.1016/j.colsurfb.2016.01.032 PMC4957969 26836480

[54] M. Huo , H. Wang , Y. Zhang , H. Cai , P. Zhang , L. Li , J. Zhou , T. Yin , J. Controlled Release 2020, 321, 198. 10.1016/j.jconrel.2020.02.017 32044390

[55] G. Corso , J. Figueiredo , S. P. De Angelis , F. Corso , A. Girardi , J. Pereira , R. Seruca , B. Bonanni , P. Carneiro , G. Pravettoni , E. Guerini Rocco , P. Veronesi , G. Montagna , V. Sacchini , S. Gandini , J. Cell. Mol. Med. 2020, 24, 5930. 32301282 10.1111/jcmm.15140 PMC7294130

[56] I. B. Vergote , C. Marth , R. L. Coleman , Cancer Metastasis Rev. 2015, 34, 41. 25564455 10.1007/s10555-014-9539-8

[57] B. Cao , L. Liu , R. Zhang , H. Dong , J. Shen , Postgrad. Med. J. 2023, 100, 112. 10.1093/postmj/qgad111 37973392

[58] S. J. Bray , Nat. Rev. Mol. Cell Biol. 2006, 7, 678. 16921404 10.1038/nrm2009

[59] T. Huang , Y. Zhou , A. S. L. Cheng , J. Yu , K. F. To , W. Kang , Mol Cancer 2016, 15, 80. 27938406 10.1186/s12943-016-0566-7 PMC5148895

[60] H. Hemati , J. Kaur , R. C. Sobti , N. Trehanpati , Biochem. Biophys. Res. Commun. 2020, 525, 941. 32173531 10.1016/j.bbrc.2020.03.009

[61] J. Helleman , H. Burger , cbt 2006, 5, 943. 10.4161/cbt.5.8.2876 16775422

[62] G. Bellot , R. Garcia‐Medina , P. Gounon , J. Chiche , D. Roux , J. Pouysségur , N. M. Mazure , Mol. Cell. Biol. 2009, 29, 2570. 19273585 10.1128/MCB.00166-09 PMC2682037

[63] M. C. Ludikhuize , M. Meerlo , M. P. Gallego , D. Xanthakis , M. Burgaya Julià , N. T. B. Nguyen , E. C. Brombacher , N. Liv , M. M. Maurice , J.i‐H. Paik , B. M. T. Burgering , M. J. Rodriguez Colman , Cell Metab. 2020, 32, 889. 33147486 10.1016/j.cmet.2020.10.005

[64] P. Maycotte , S. Aryal , C. T. Cummings , J. Thorburn , M. J. Morgan , A. Thorburn , Autophagy 2012, 8, 200. 22252008 10.4161/auto.8.2.18554 PMC3336076

[65] T. Saito , K. Hamano , J. Sadoshima , Cardiovasc. Res. 2020, 117, 2730. 10.1093/cvr/cvaa340 PMC8932294 33331644

[66] J. Liu , X. Li , Y. Li , Q. Gong , K. Luo , Theranostics 2025, 15, 993. 39776799 10.7150/thno.104872 PMC11700864

[67] J. Liu , Y. Bai , Y. Li , X. Li , K. Luo , eBioMedicine 2024, 107, 105301. 39178747 10.1016/j.ebiom.2024.105301 PMC11388279

[68] X. Li , M. Li , M. Huang , Q. Lin , Q. Fang , J. Liu , X. Chen , L. Liu , X. Zhan , H. Shan , et al., Biomedicine & Pharmacotherapy 2022, 150, 113064. 35658234 10.1016/j.biopha.2022.113064

[69] K. Bukowski , M. Kciuk , R. Kontek , Int. J. Mol. Sci. 2020, 21, 3233. 32370233 10.3390/ijms21093233 PMC7247559

[70] R. S. Garcia Ribeiro , S. Belderbos , P. Danhier , J. Gallo , B. Manshian , B. Gallez , M. Bañobre , M. de Cuyper , S. Soenen , W. Gsell , U. Himmelreich , Int. J. Nanomed. 2019, 14, 5911. 10.2147/IJN.S214041 PMC6681073 31534330

[71] Z. Zhu , Y. Zhai , Y. Hao , Q. Wang , F. Han , W. Zheng , J. Hong , L. Cui , W. Jin , S. Ma , et al., J of Extracellular Vesicle 2022, 11, 12255. 10.1002/jev2.12255 PMC9451528 35932288

[72] X. Du , Z. Cheng , Y. Wang , Z. Guo , S. Zhang , J. Hu , Z. Zhou , World J. Gastroenterol. 2014, 20, 9191. 25083094 10.3748/wjg.v20.i27.9191 PMC4112896

[73] Z. Wang , Y. Li , D. Kong , F. H. Sarkar , Curr. Drug Targets 2010, 11, 745. 20041844 10.2174/138945010791170860 PMC3084452

[74] D. A. Kubli , Å. B. Gustafsson , Circ. Res. 2012, 111, 1208. 23065344 10.1161/CIRCRESAHA.112.265819 PMC3538875

[75] M. Onishi , K. Yamano , M. Sato , N. Matsuda , K. Okamoto , EMBO J. 2021, 40, 104705. 10.15252/embj.2020104705 PMC7849173 33438778

[76] Y. Lu , Z. Li , S. Zhang , T. Zhang , Y. Liu , L. Zhang , Theranostics 2023, 13, 736. 36632220 10.7150/thno.79876 PMC9830443

[77] W. Li , Y. Li , S. Siraj , H. Jin , Y. Fan , X. Yang , X. Huang , X. Wang , J. Wang , L. Liu , L. Du , Q. Chen , Hepatology 2019, 69, 604. 30053328 10.1002/hep.30191

