# Supplementary material for: WSGC@FA@PEG/PEI‐SPIONs Mitigate Chemoresistance in Gastric Adenocarcinoma by Modulating the Notch Signaling Pathway and Mitophagy
Source: Adv Sci (Weinh). 2025 Aug 19;12(36):e15840. doi: 10.1002/advs.202415840 (PMC12463016; doi:10.1002/advs.202415840)
Supplement: Supplementary file 1 — Supporting Information [file ADVS-12-e15840-s002.docx]

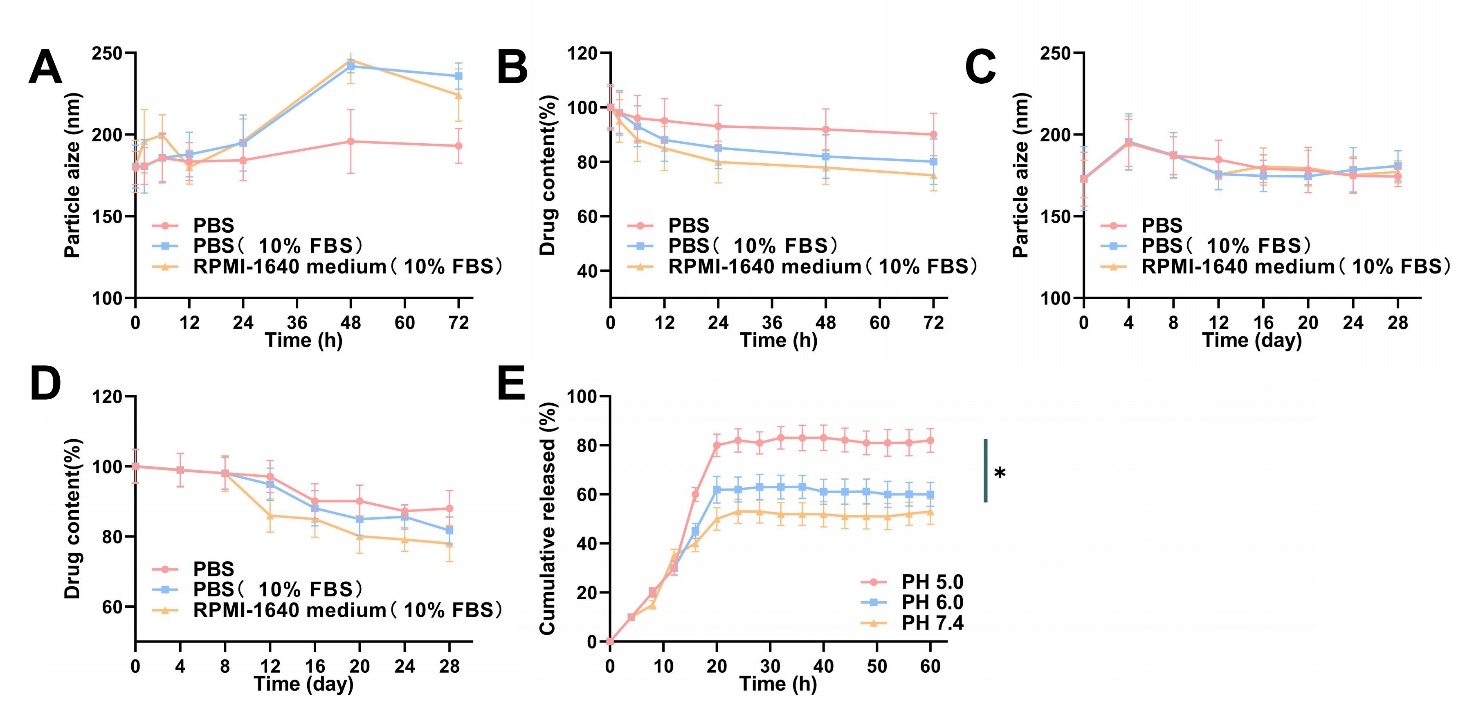


**Figure S1. Study on the stability of WSGC@FA@PEG/PEI-SPIONs.**

Note: (A-B) Relative stability of particle size (A) and WSGC release amount (B) of WSGC@FA@PEG/PEI-SPIONs in 0.01 mol/L PBS solution, PBS solution containing 10% fetal bovine serum, and complete cell culture medium containing 10% fetal bovine serum at 37°C for 0-72 hours; (C-D) Relative stability of particle size (C) and WSGC release amount (D) of WSGC@FA@PEG/PEI-SPIONs in 0.01 mol/L PBS solution, PBS solution containing 10% fetal bovine serum, and complete cell culture medium containing 10% fetal bovine serum at 4°C for 0-28 hours; (E) *In vitro* drug release profile of WSGC@FA@PEG/PEI-SPIONs in PBS solutions at pH 7.3, 6.0, and 5.0; * indicates statistically significant difference between two groups, **p*<0.05; all cell experiments were performed in triplicate (n=3). The data is presented in the form of Mean ± SD; Two factor analysis of variance was used for data at different time points


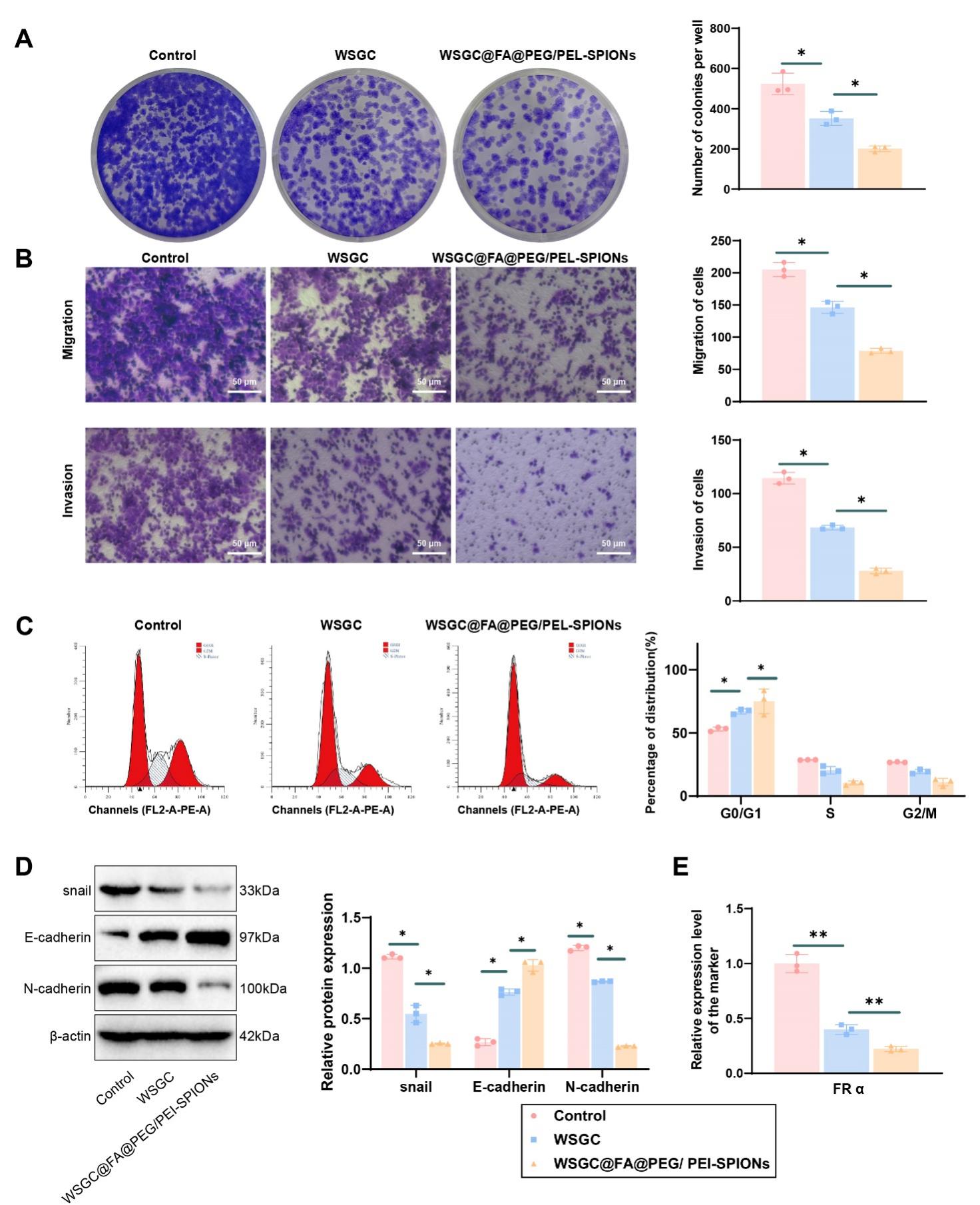


**Figure S2. Effects of WSGC@FA@PEG/PEI-SPIONs on proliferation, migration, and invasion of GA cell line MNK-45.**

Note: (A) Clonogenic assay to measure the colony formation ability of MNK-45 cells in different groups; (B) Transwell assay to assess the migration and invasion capabilities of MNK-45 cells in different groups, Scale bar=50μm; (C) Cell cycle analysis of MNK-45 cells in different groups by flow cytometry; (D) Western blot analysis of the protein expression levels of Snail, E-cadherin, and N-cadherin in MNK-45 cells in different groups; **p*<0.05; all cell experiments were performed in triplicate. Data were presented as mean ± SD. Comparisons among three or more groups were performed using one-way ANOVA.

**
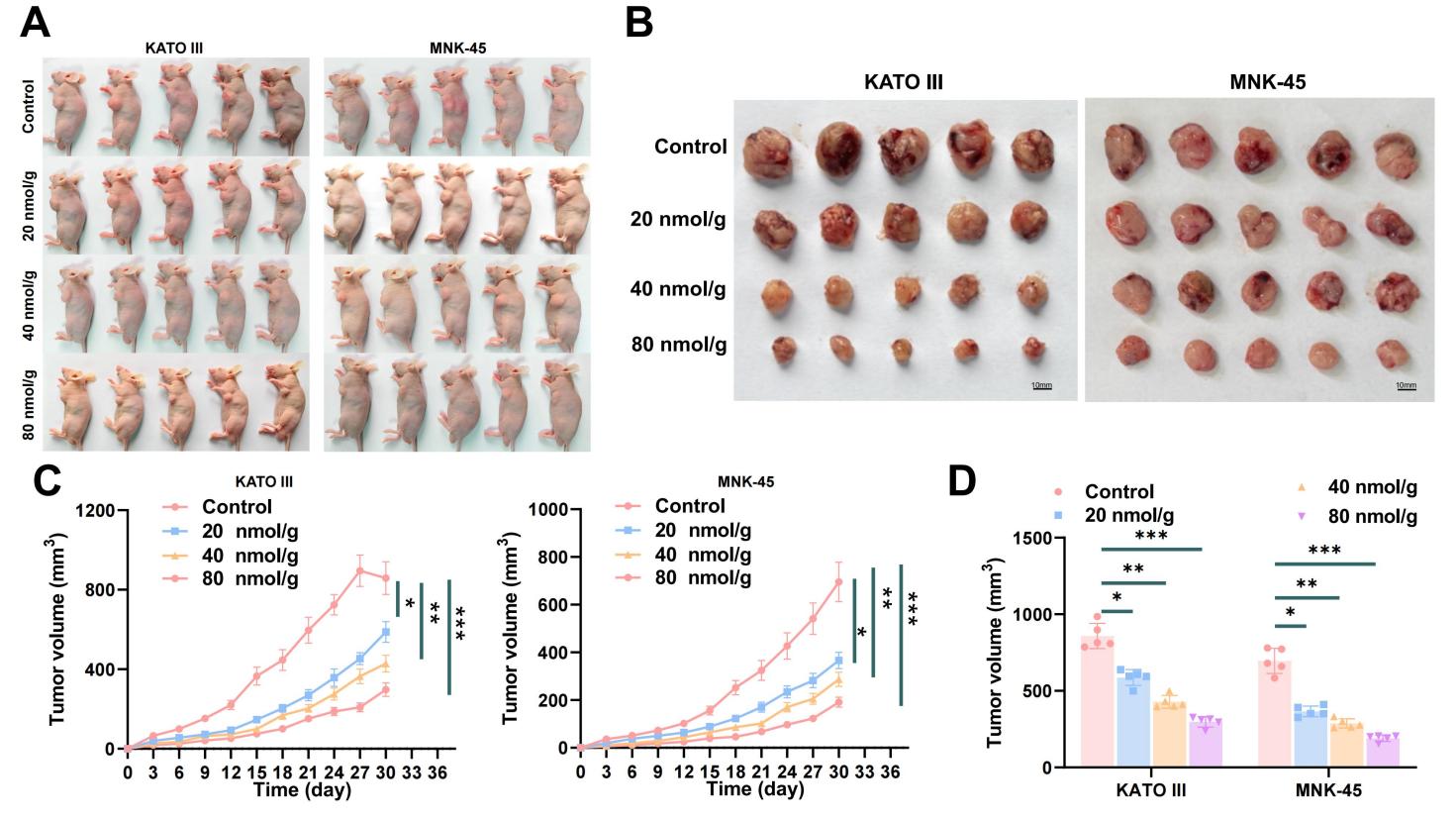
**

**Figure S3. Intervention of GA in immunodeficient mice model with WSGC@FA@PEG/PEI-SPIONs.**

Note: (A) Construction of GA xenograft mouse models using BALB/C nude mice injected with KATO III and MNK-45 cells; (B) Tumor resection, weight measurement, and imaging; (C) Measurement of Tumor longitudinal and transverse diameters every 3 days with calipers, calculation of tumor volume, and plotting tumor growth curves for KATO III (left) and MNK-45 (right) tumors; (D) Statistical analysis of tumor volumes with data represented as mean ± SD (n=5); * denotes significant difference between two groups, **p*<0.05, ***p*<0.01, ****p*<0.001; sample size n=5. Data are presented as mean ± SD. One-way ANOVA was used to compare data among different time groups, while repeated measures ANOVA was applied for tumor volume comparisons at different time points.

**
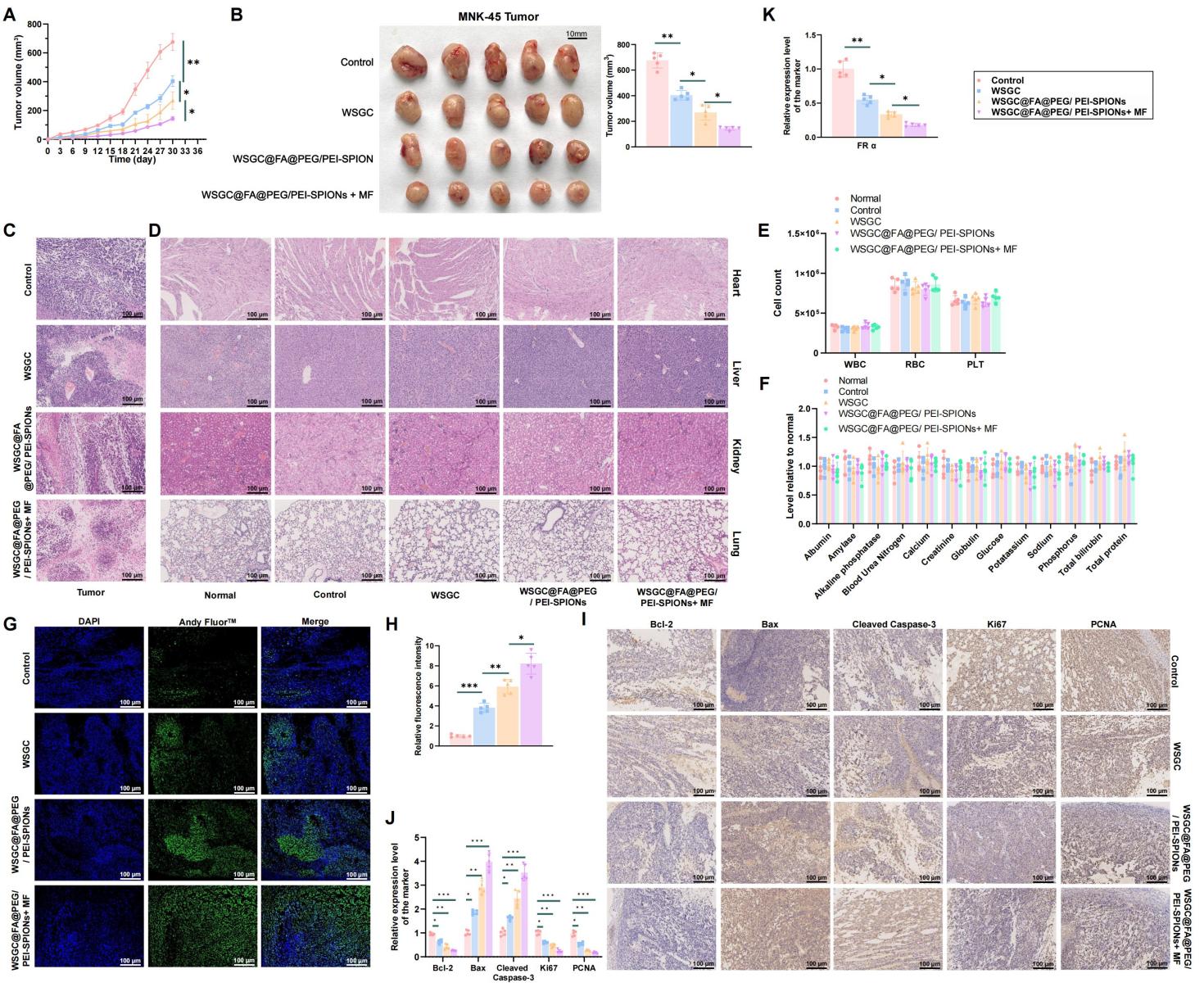
**

**Figure S4. *In vivo* antitumor efficacy of WSGC@FA@PEG/PEI-SPIONs against MNK-45 tumors.**

Note: (A) Relationship between tumor volumes under four different treatment regimens over time; (B) Tumor images collected on day 30 under the four different treatment conditions; (C) Representative micrographs of H&E-stained tumors displaying reduced tumor cell count in WSGC@FA@PEG/PEI-SPIONs+MF treatment group compared to the other three treatment methods; (D) Representative micrographs of H&E-stained major organs in normal mice and GA model mice treated with the four different regimens; (E) Statistical graphs of peripheral blood parameters (WBC, RBC, PLT) from Normal mice and GA model mice after different treatments; (F) Serum biochemical parameters including Albumin, Amylase, Alkaline Phosphatase, Blood Urea Nitrogen, Calcium, Creatinine, Globulin, Glucose, Potassium, Sodium, Phosphorus, Total Bilirubin, and Total Protein in Normal mice and GA model mice following different treatments; (G) TUNEL staining of apoptotic cells in tumor tissues of KATO III xenograft mice model; (H) Statistical analysis of apoptosis in different treatment groups; (I) Immunohistochemical analysis of the protein expression levels of Bax, Cleaved Caspase-3, Bcl-2, Ki67, and PCNA in tumor tissues of KATO III xenograft models (scale bar = 100 μM); (J) Statistical analysis of immunohistochemistry results; (K) RT-qPCR verified the expression of folate receptor FRα (mRNA) in tumor cells from nude mice. * indicates significant difference between two groups, **p*<0.05, ***p*<0.01; sample size n=5. Data are presented as mean ± SD. One-way ANOVA was used to compare data among different time groups, and repeated measures ANOVA was applied for tumor volume comparisons at different time points.


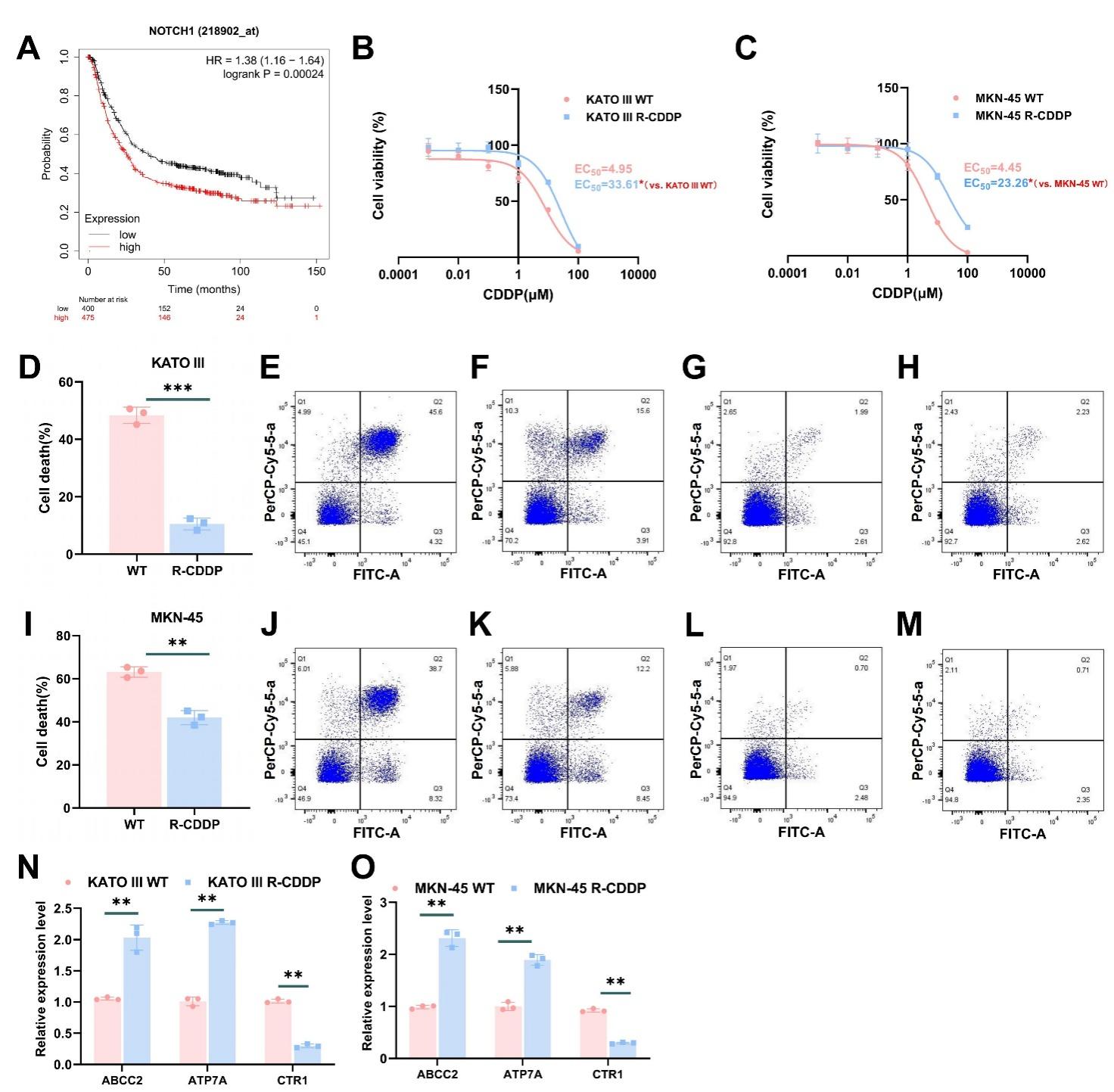


**Figure S5. Construction of drug-resistant GA cells.**

Note: (A) Expression of NOTCH1 (218902_at) and patient survival analysis (n=875); (B-C) Drug sensitivity of drug-resistant and parental cell lines assessed by MTT assay, dose-response curves of cisplatin-resistant KATO III cells (KATO III R-CDDP) and parental KATO III cells (KATO III WT) (B), and dose-response curves of cisplatin-resistant MNK-45 cells (MNK-45 R-CDDP) and parental MNK-45 cells (MNK-45 WT) (C); (D) Percentage of cell death in KATO III WT compared to CDDP-resistant KATO III cells (KATO III R-CDDP), including annexin V+/PI- cells in Q4 and annexin V+/PI+ cells in Q2; (E) Representative dot plot of KATO III WT treated with 26.05 μM CDDP; (F) Representative dot plot of KATO III R-CDDP treated with 26.05 μM CDDP; (G) Representative dot plot of untreated control KATO III WT; (H) Representative dot plot of untreated control KATO III R-CDDP; (I) Percentage of cell death in MNK-45 WT compared to CDDP-resistant MNK-45 cells (MNK-45 R-CDDP), including annexin V+/PI- cells in Q4 and annexin V+/PI+ cells in Q2; (J) Representative dot plot of MNK-45 WT treated with 26.05 μM CDDP; (K) Representative dot plot of MNK-45 R-CDDP treated with 26.05 μM CDDP; (L) Representative dot plot of untreated control MNK-45 WT; (M) Representative dot plot of untreated control MNK-45 R-CDDP;(N) Relative expression levels of ABCC2, ATP7A, and CTR1 genes in KATO III WT and KATO III R-CDDP; (O) Relative expression levels of ABCC2, ATP7A, and CTR1 genes in MNK-45 WT and MNK-45 R-CDDP; data presented as mean ± SD from three biological replicates. Statistical significance between two groups was determined using an unpaired two-tailed t-test.


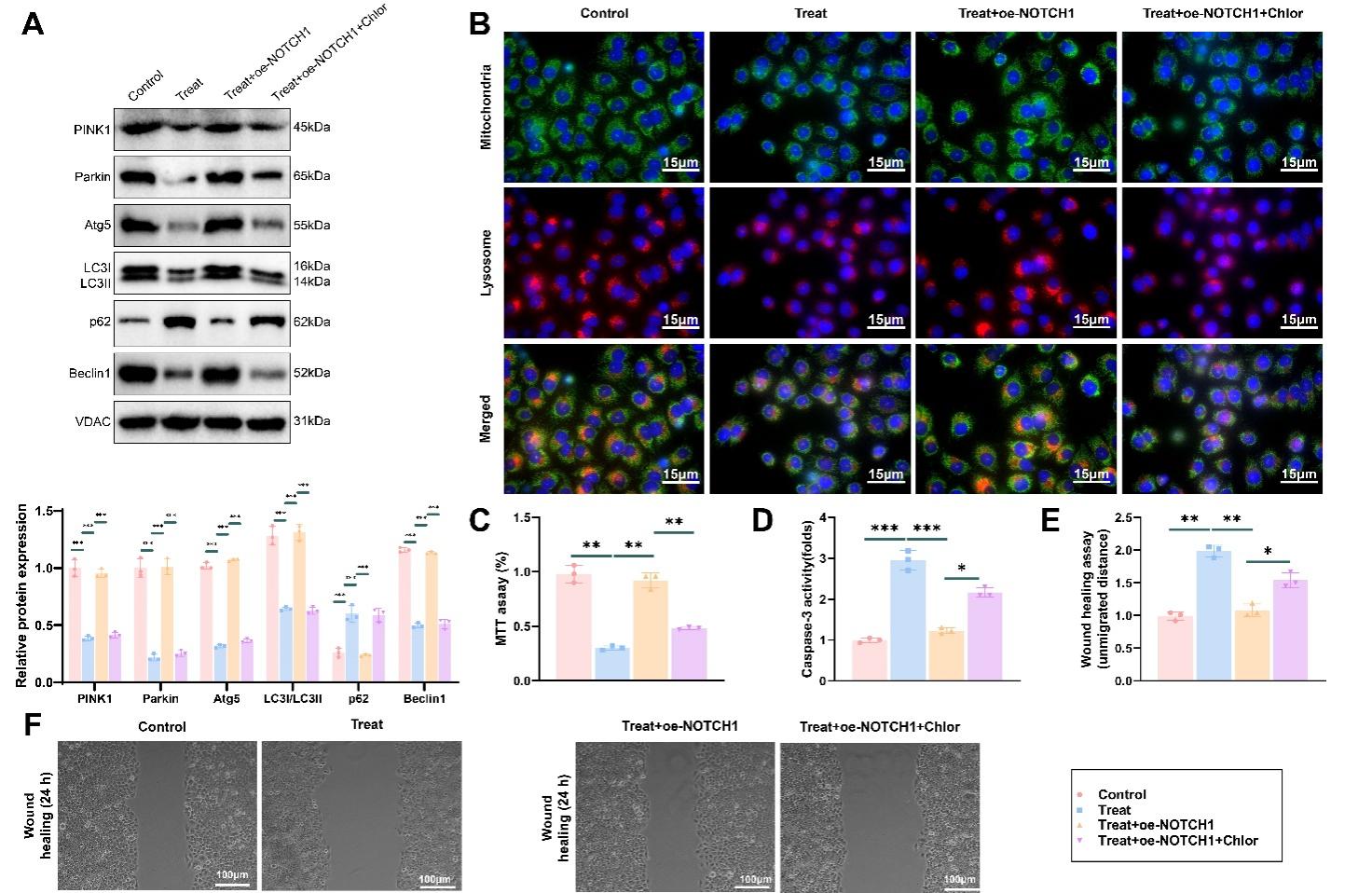


**Figure S6. WSGC peptide mediates mitophagy via the notch signaling pathway.**

Note: (A) Detection of the protein expression of mitophagy-related proteins (PINK1, Parkin, Atg5, LC3B, p62, Beclin1) in MNK-45 R-CDDP cells; (B) Evaluation of the colocalization of mitochondria and lysosomes in MNK-45 R-CDDP cells in various treatment groups, with immunofluorescent staining of mitochondria (green) and lysosomes (red) (scale bar = 100 μM/20 μM); (C) Assessment of cell viability in MNK-45 R-CDDP cells using the MTT assay; (D) Analysis of changes in Caspase-3 activity in MNK-45 R-CDDP cells; (E-F) Scratch assay to examine cell migration of MNK-45 R-CDDP cells at 24 hours post different treatments; **p*<0.05, ***p*<0.01, ****p*<0.001. All cell experiments were performed in triplicate. The data are presented as Mean ± SD. For comparisons involving three or more groups, one-way analysis of variance (ANOVA) is utilized.
