# Supplementary material for: WSGC@FA@PEG/PEI‐SPIONs Mitigate Chemoresistance in Gastric Adenocarcinoma by Modulating the Notch Signaling Pathway and Mitophagy
Source: Adv Sci (Weinh). 2025 Aug 19;12(36):e15840. doi: 10.1002/advs.202415840 (PMC12463016; doi:10.1002/advs.202415840)

# WB bands of Figure 3D-1


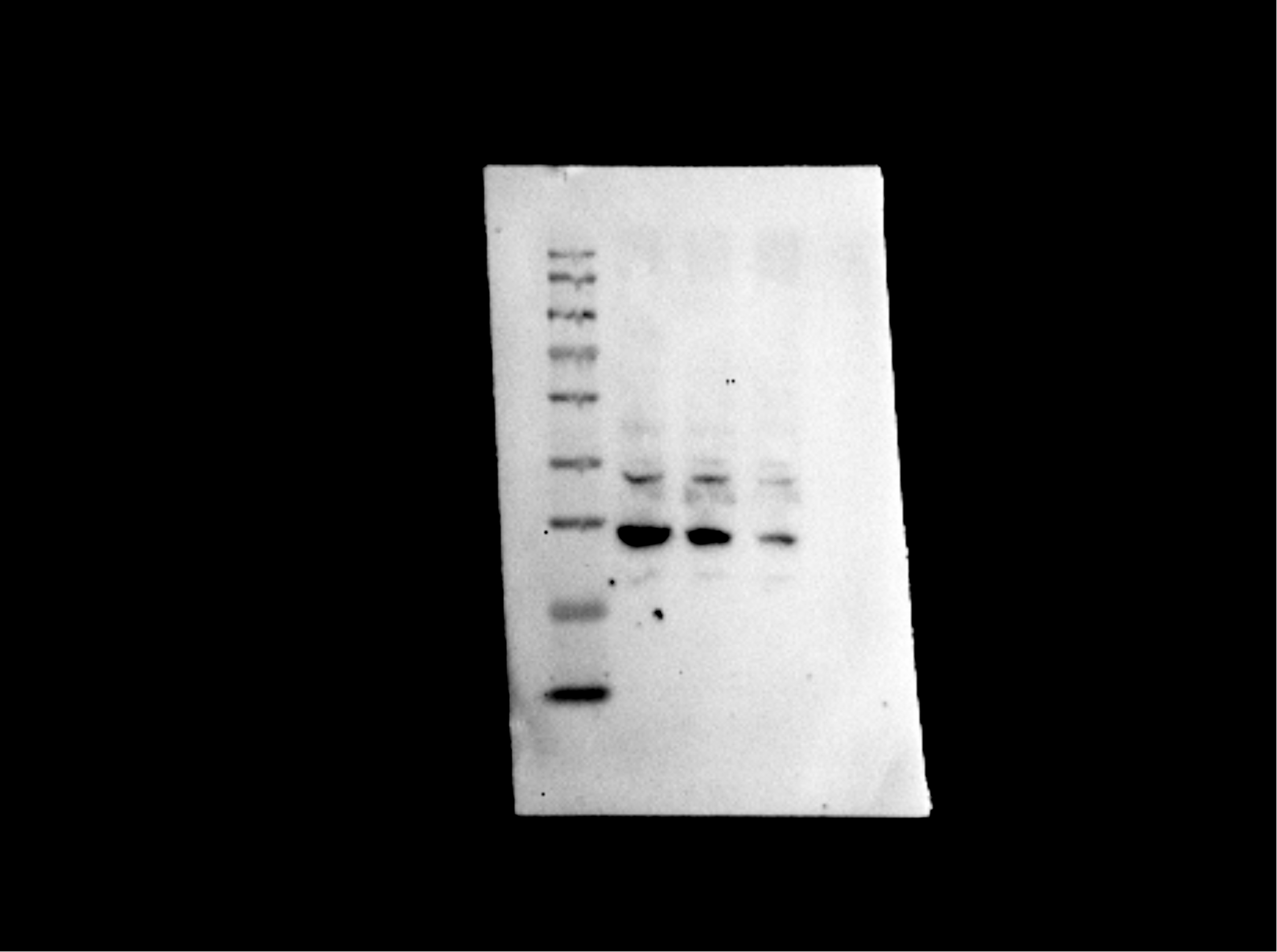


# WB bands of Figure 3D-2


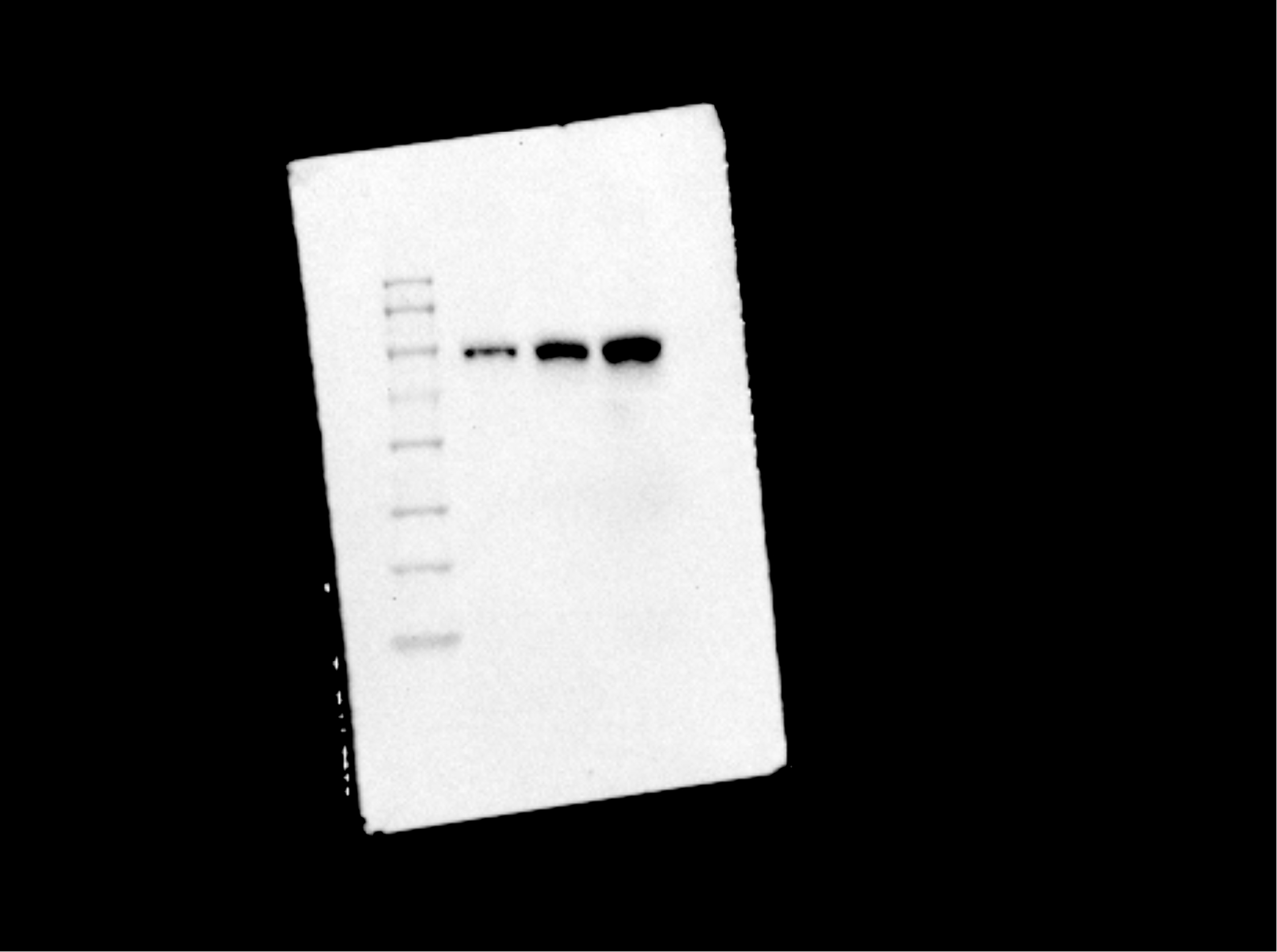


# WB bands of Figure 3D-3


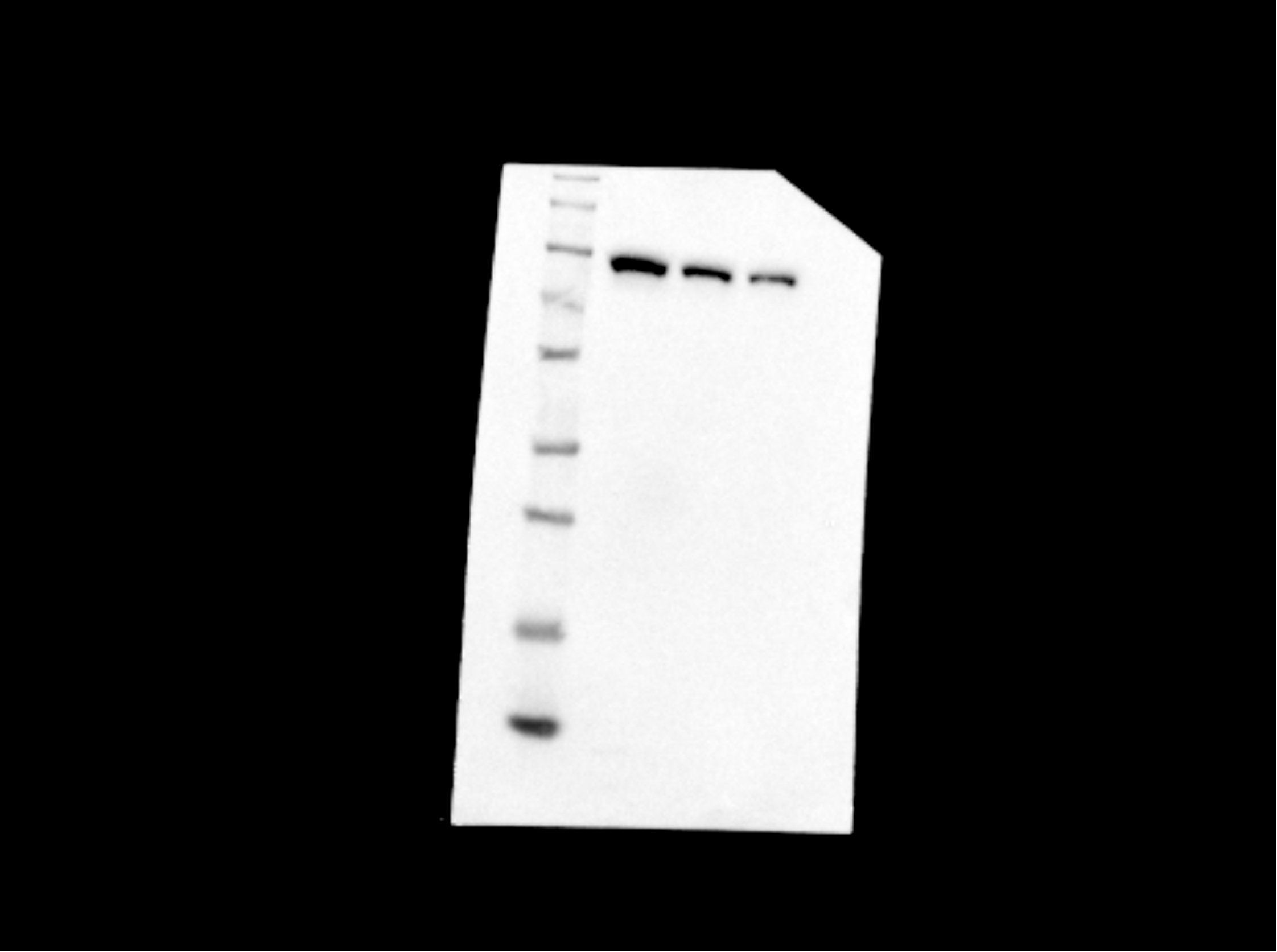


# WB bands of Figure 3D-4


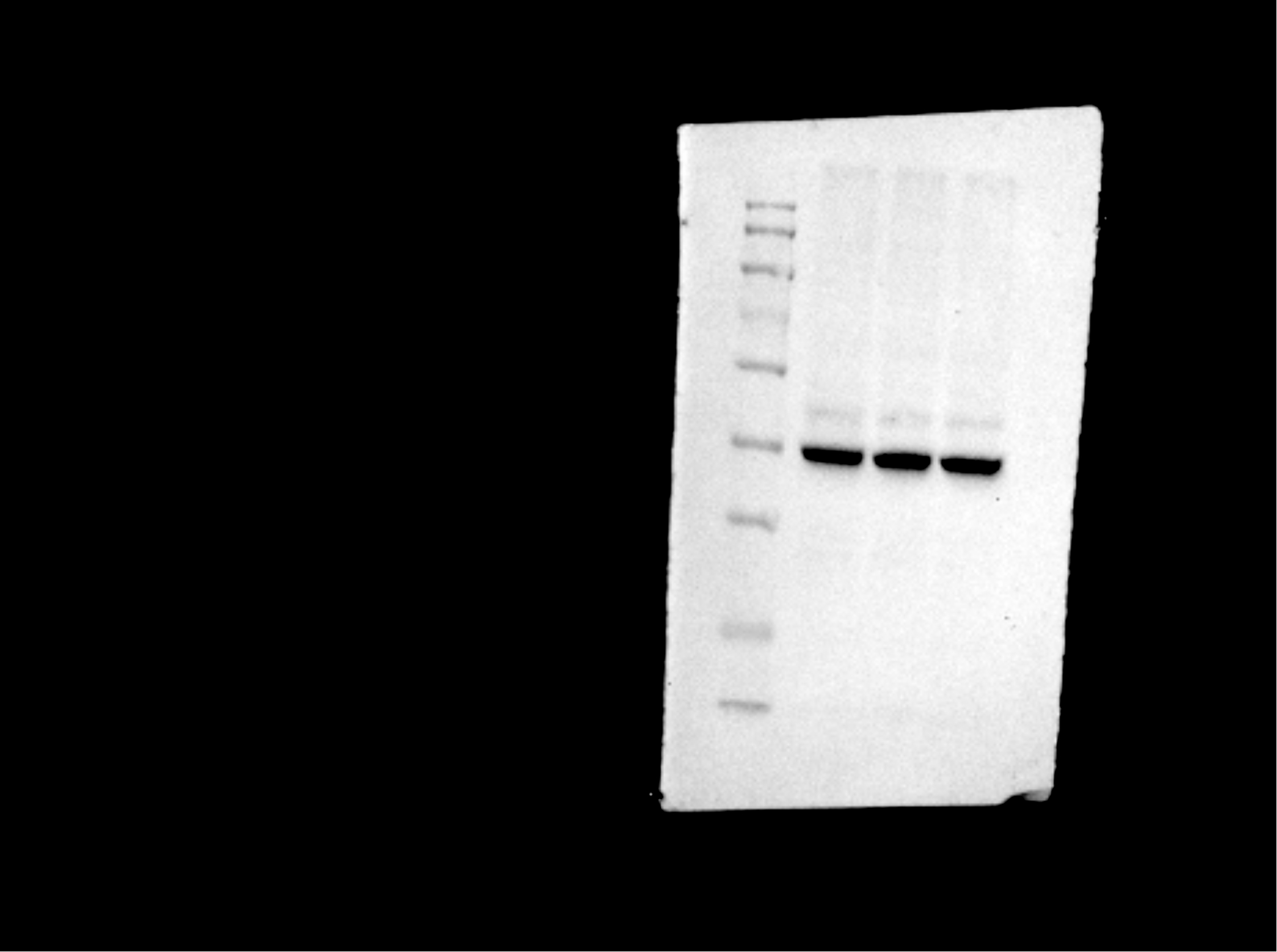


# WB bands of Figure 7A-1


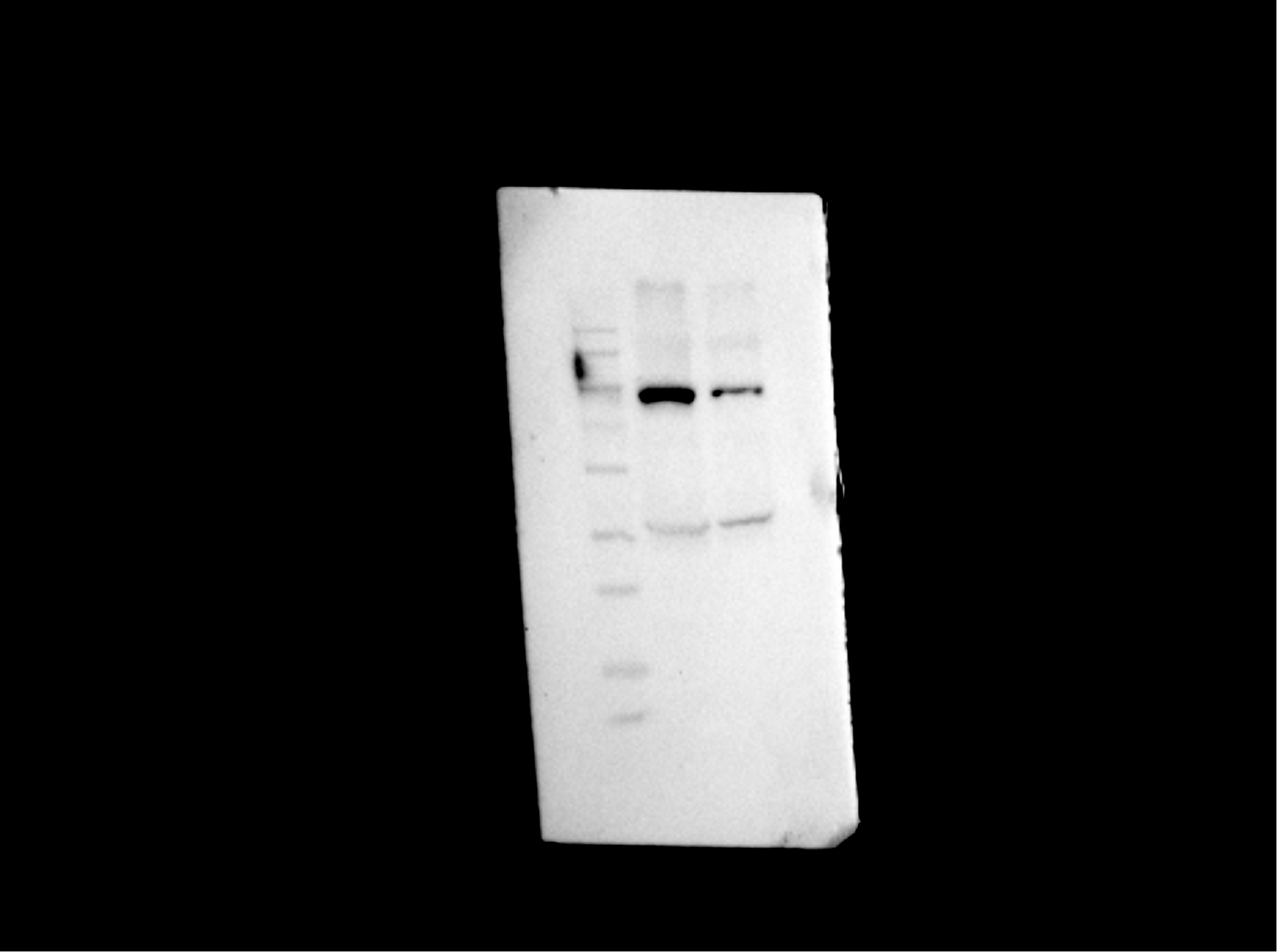


# WB bands of Figure 7A-2


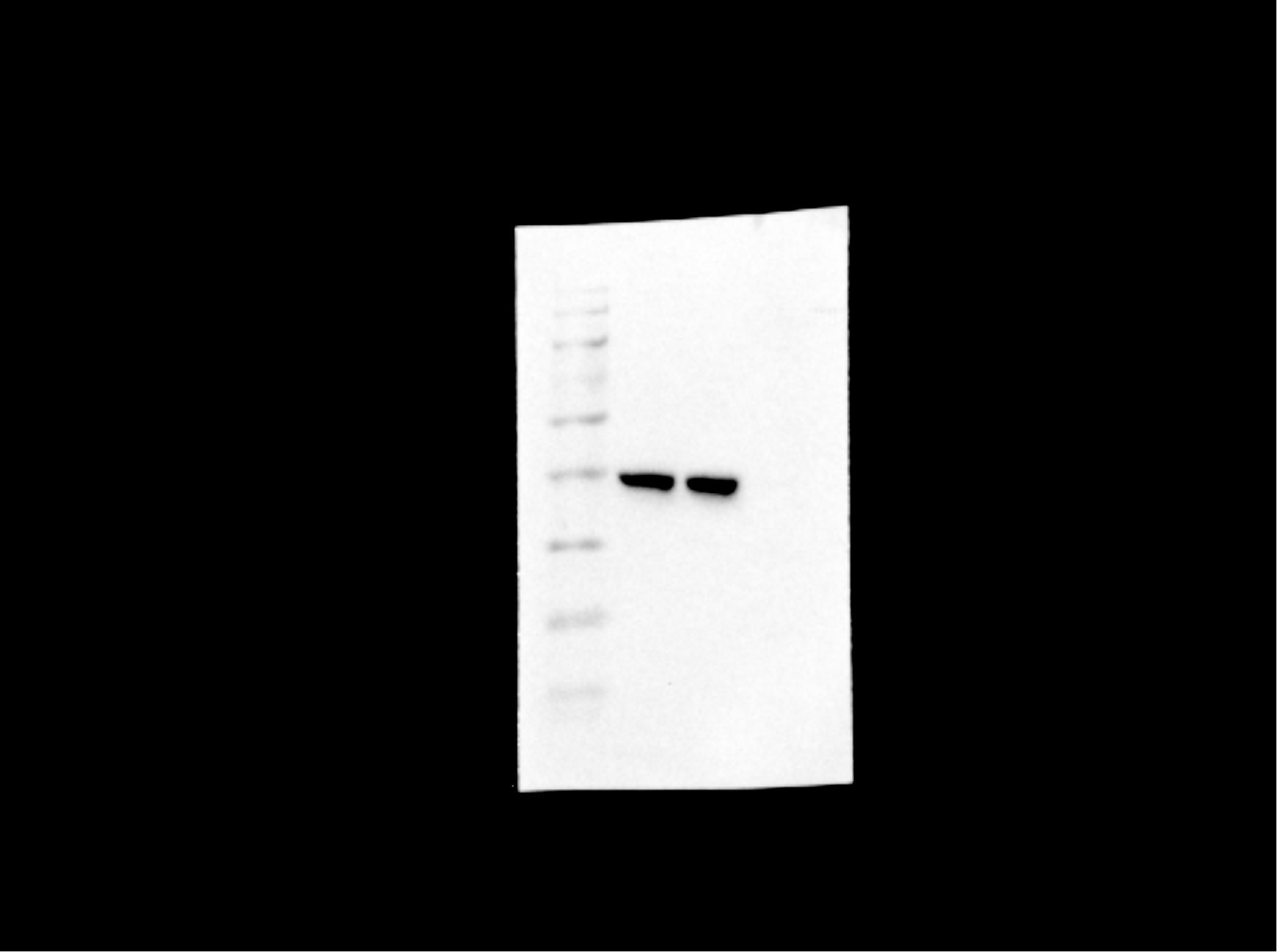


# WB bands of Figure 7B-1


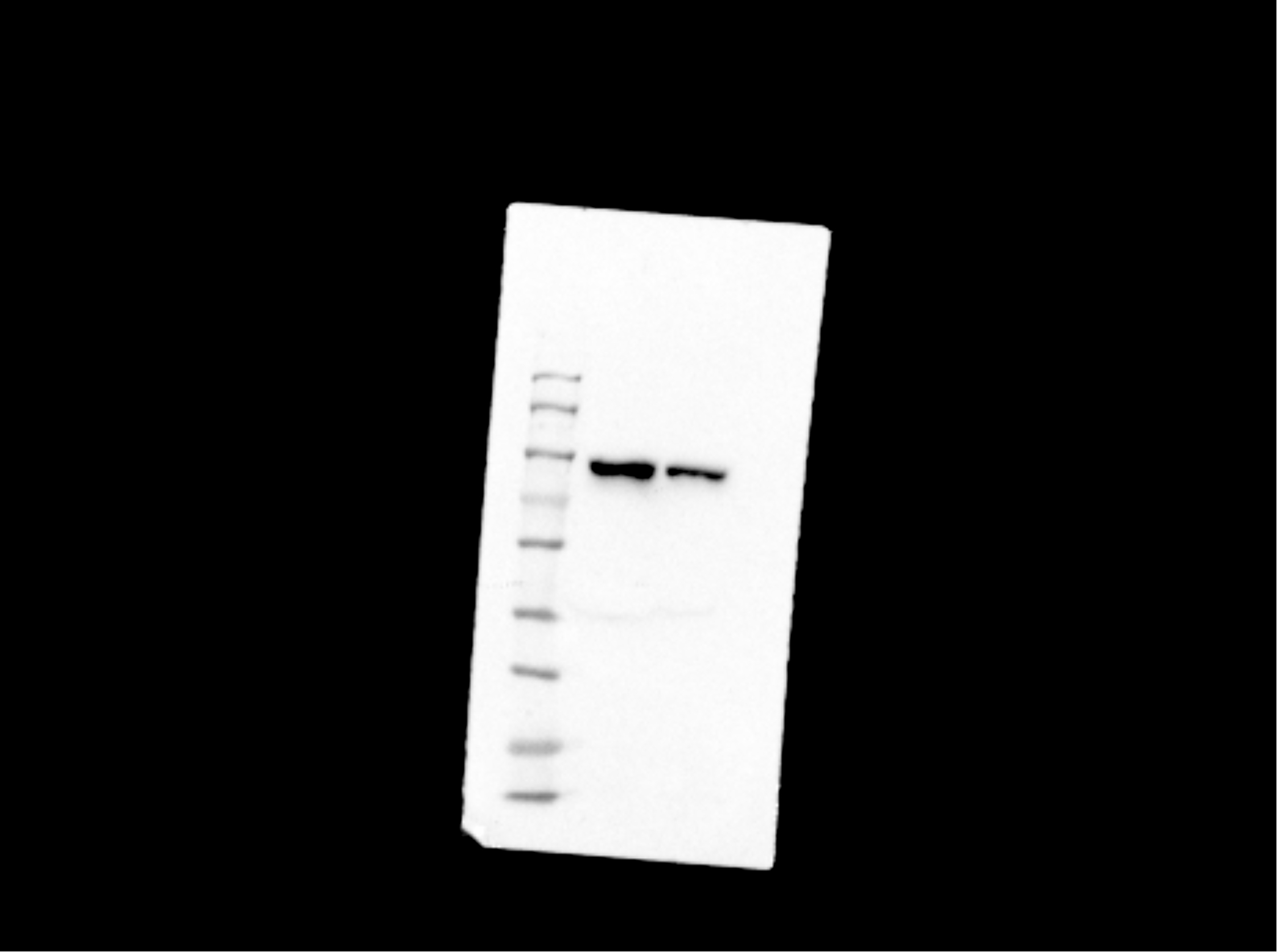


# WB bands of Figure 7B-2


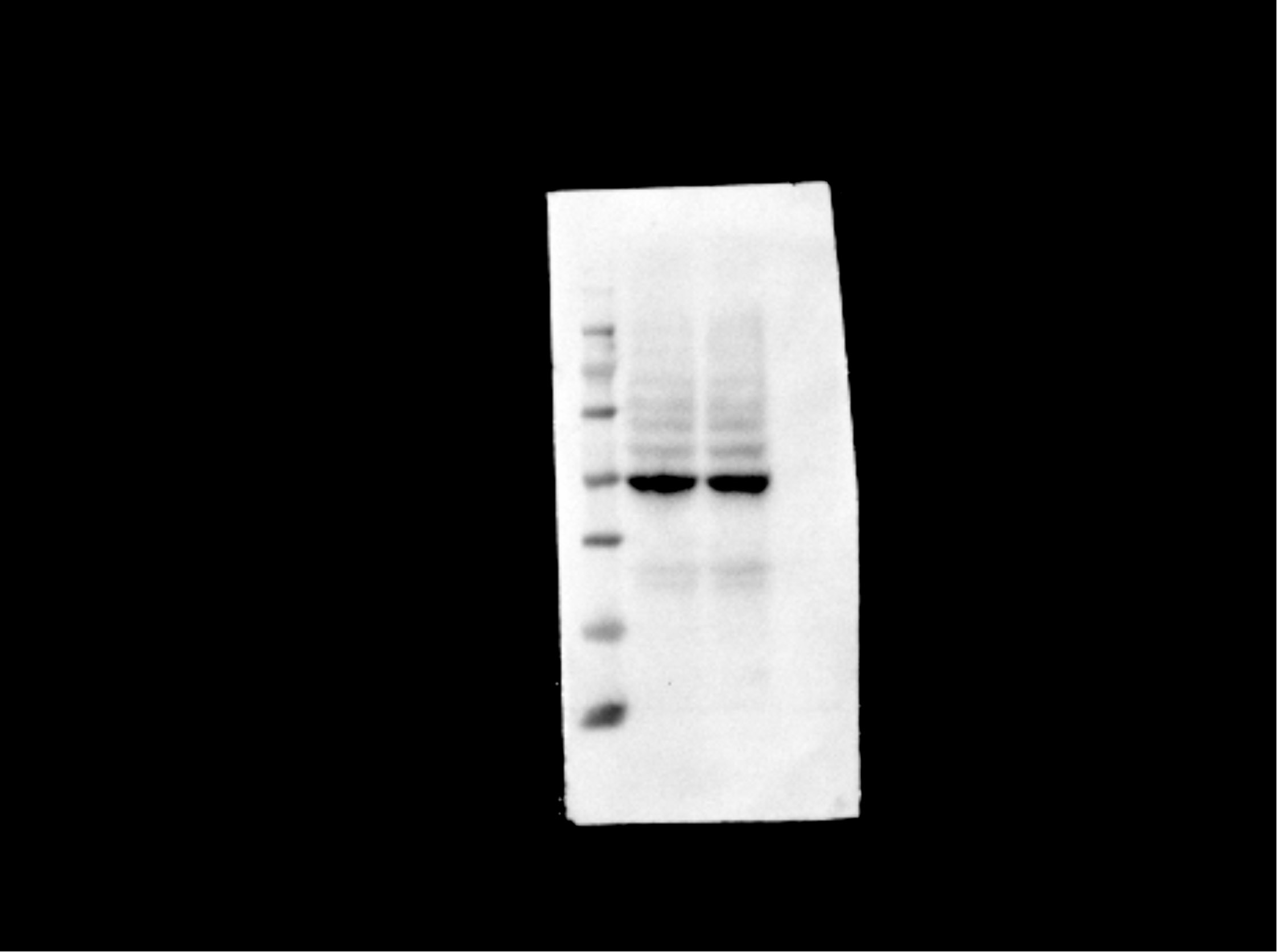


# WB bands of Figure 7E-1


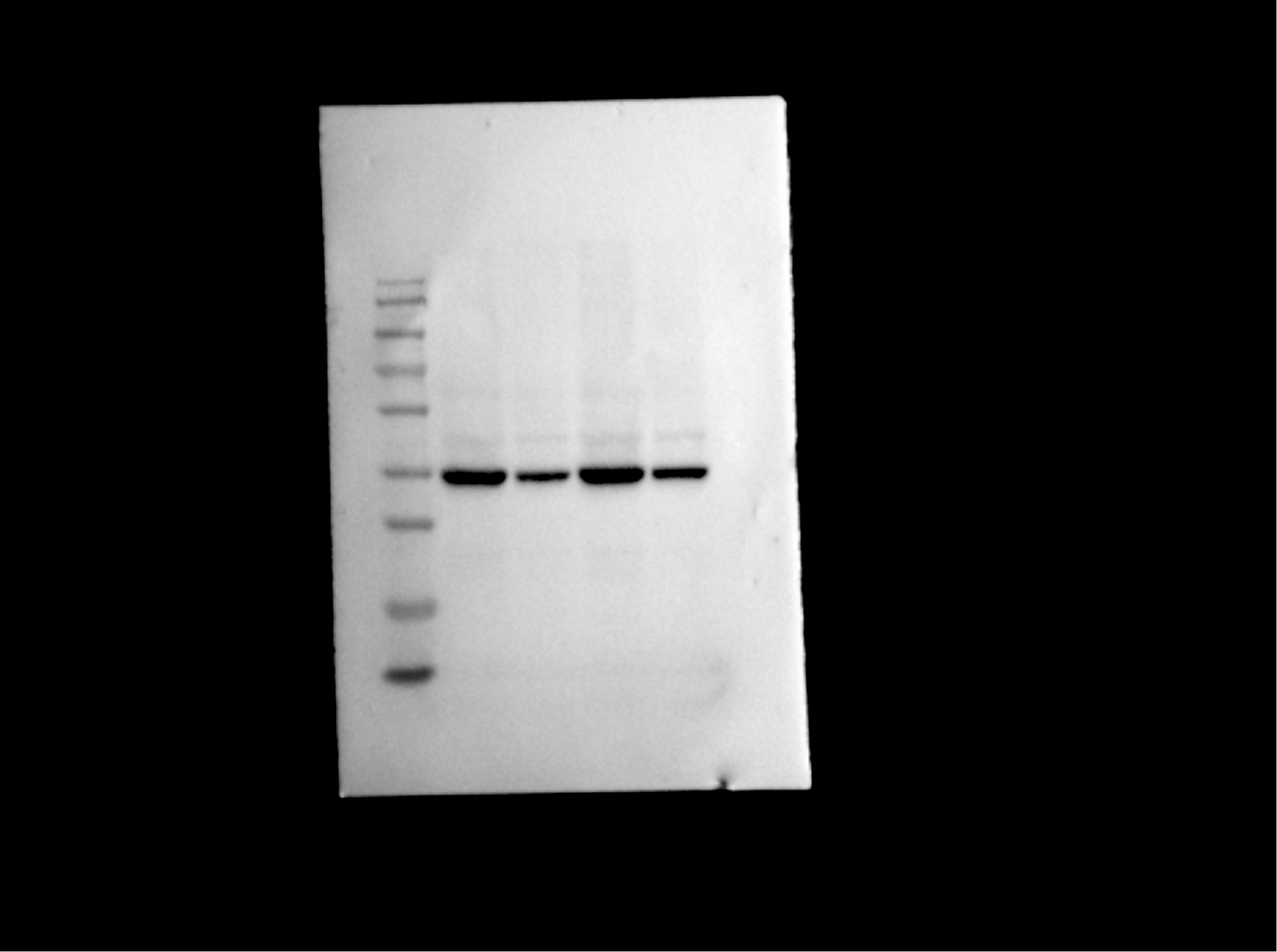


# WB bands of Figure 7E-3


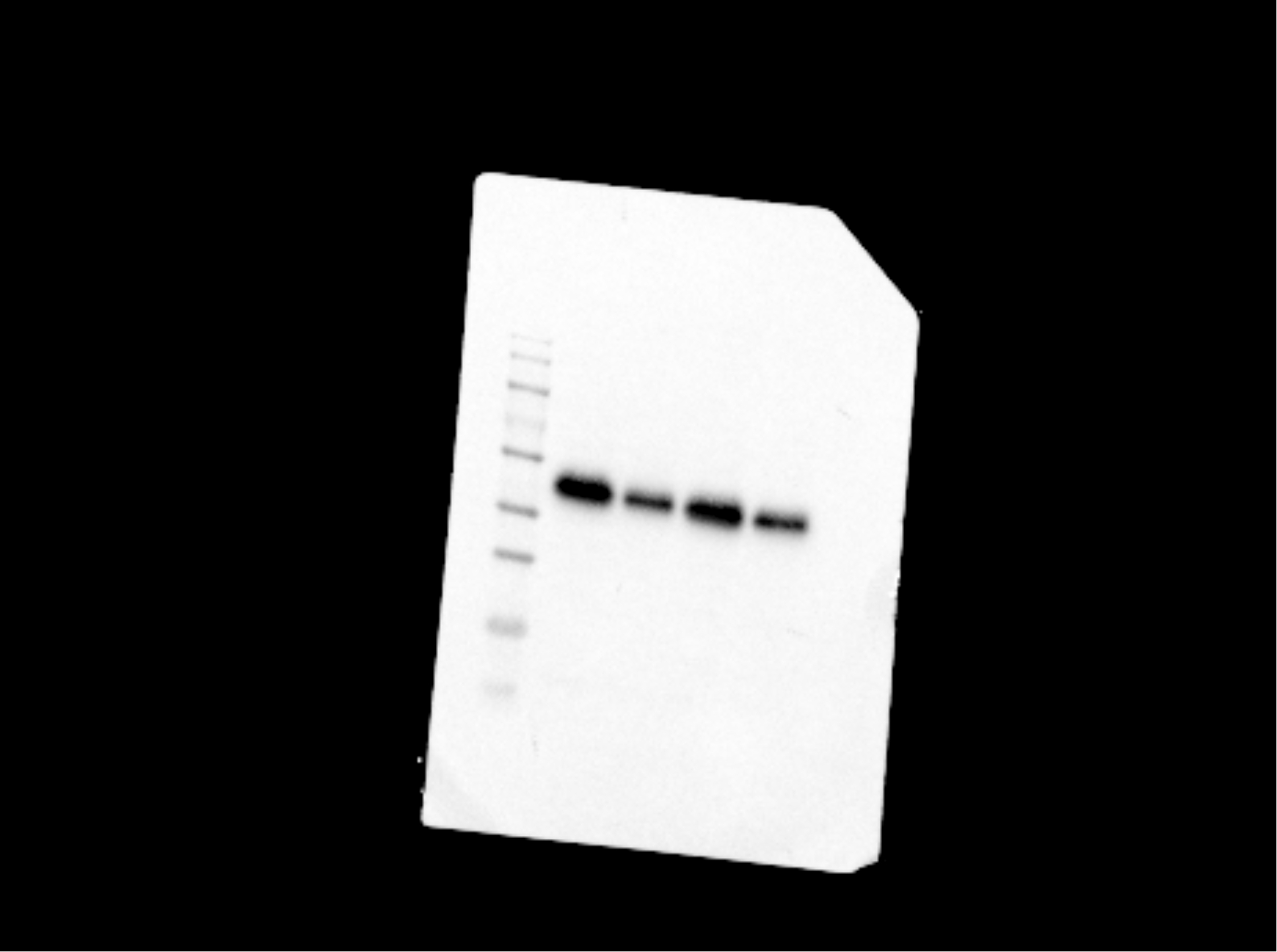


# WB bands of Figure 7E-4


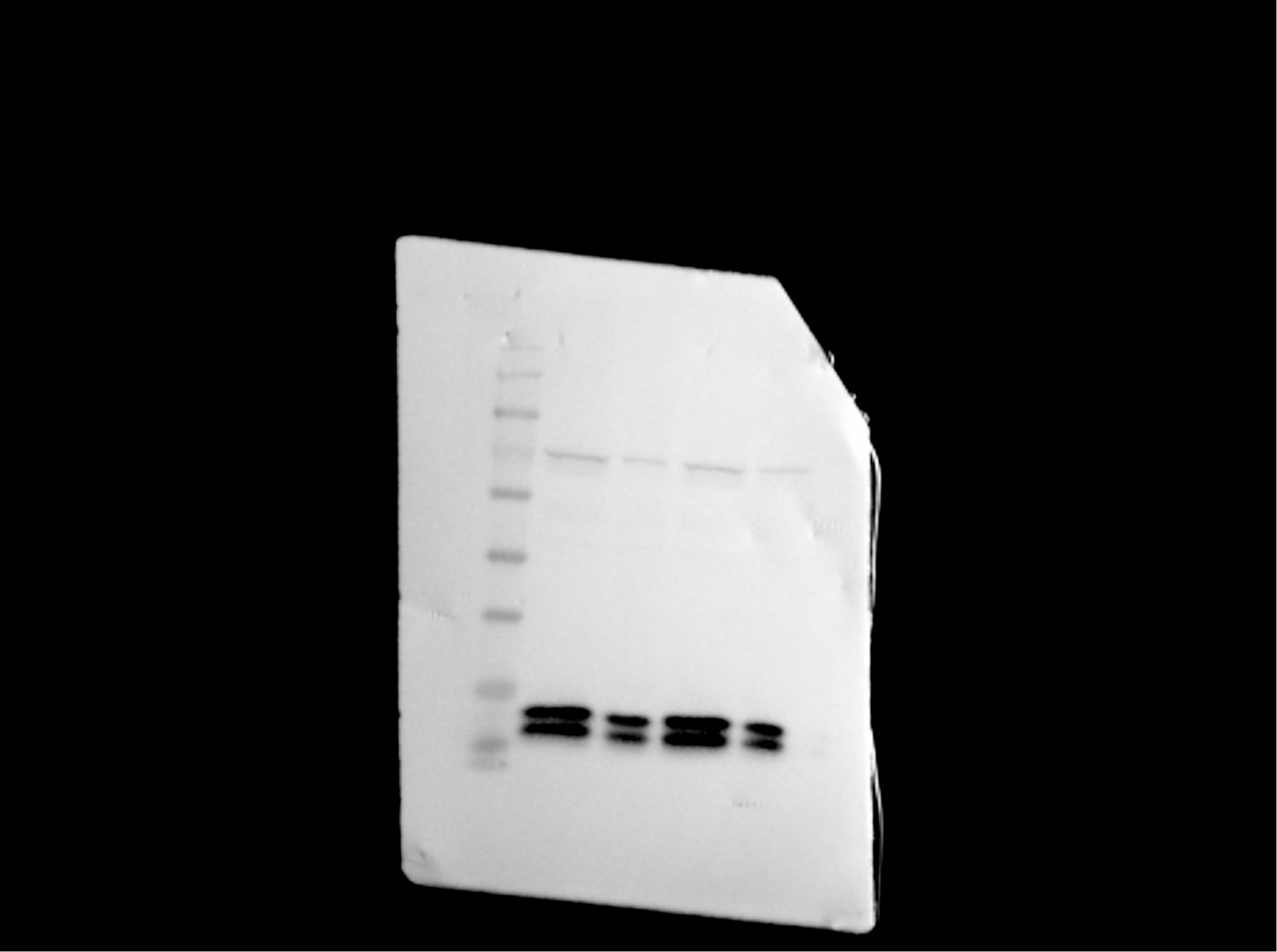


# WB bands of Figure 7E-5


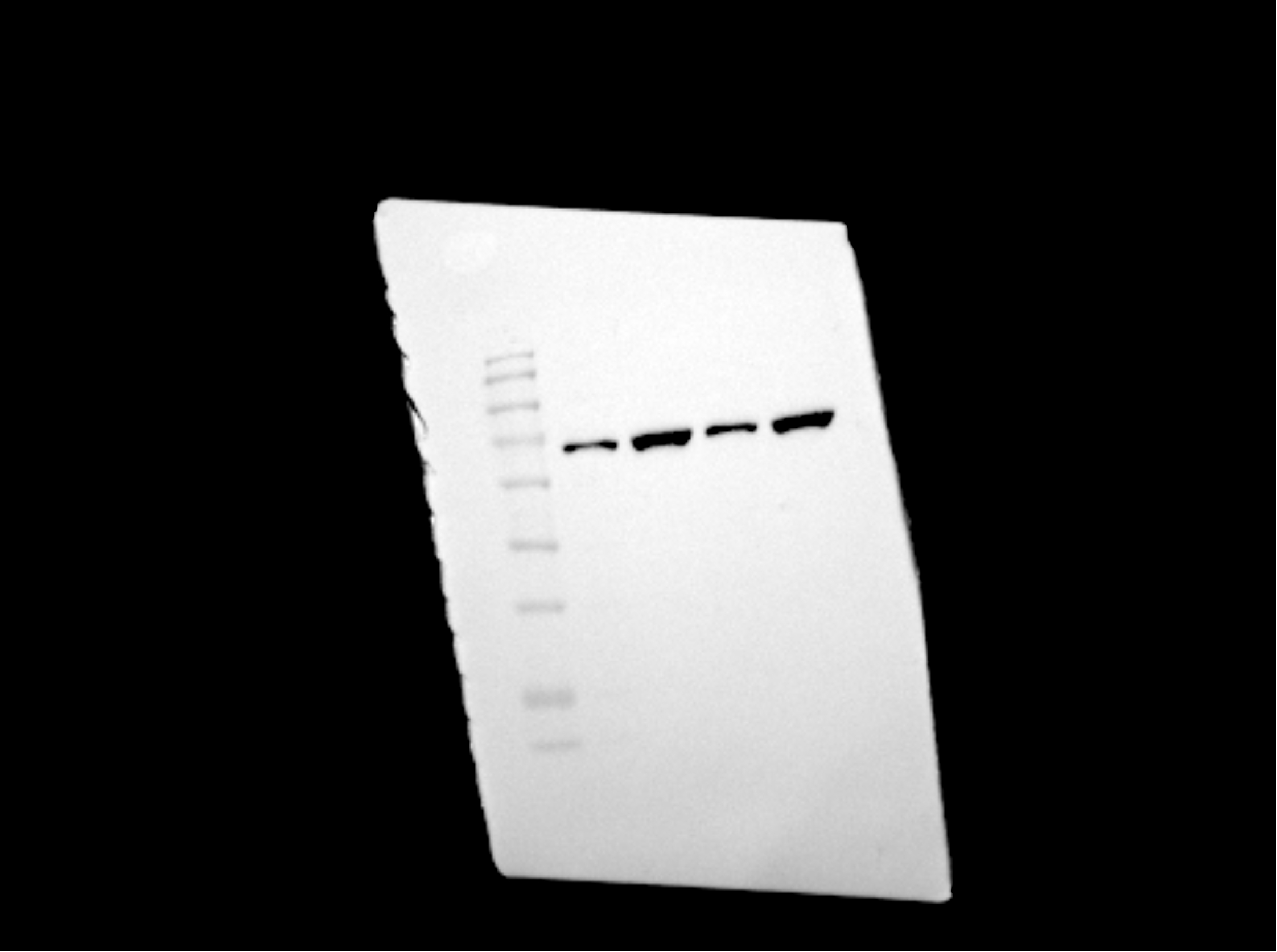


# WB bands of Figure 7E-6


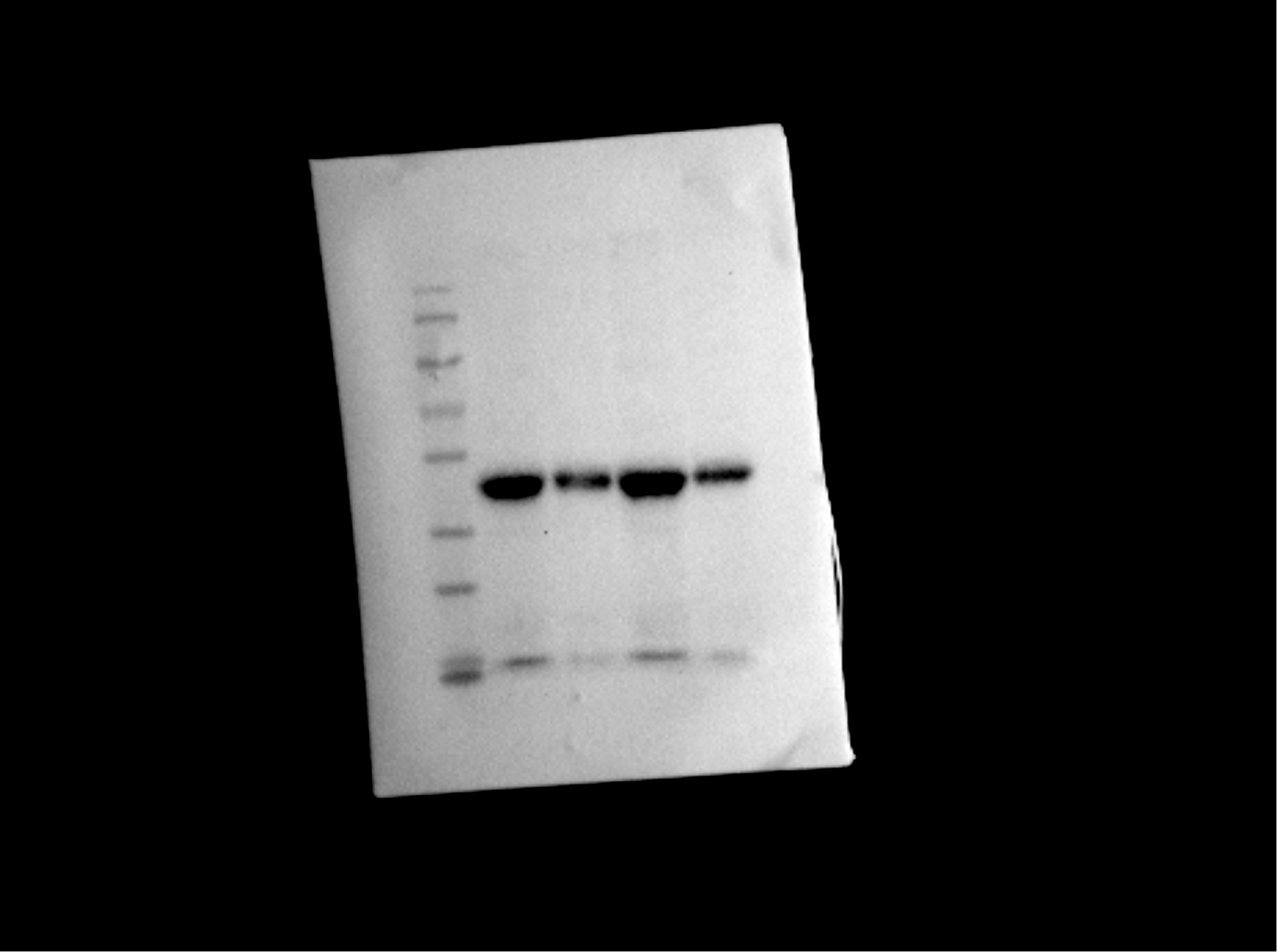


# WB bands of Figure 7E-7


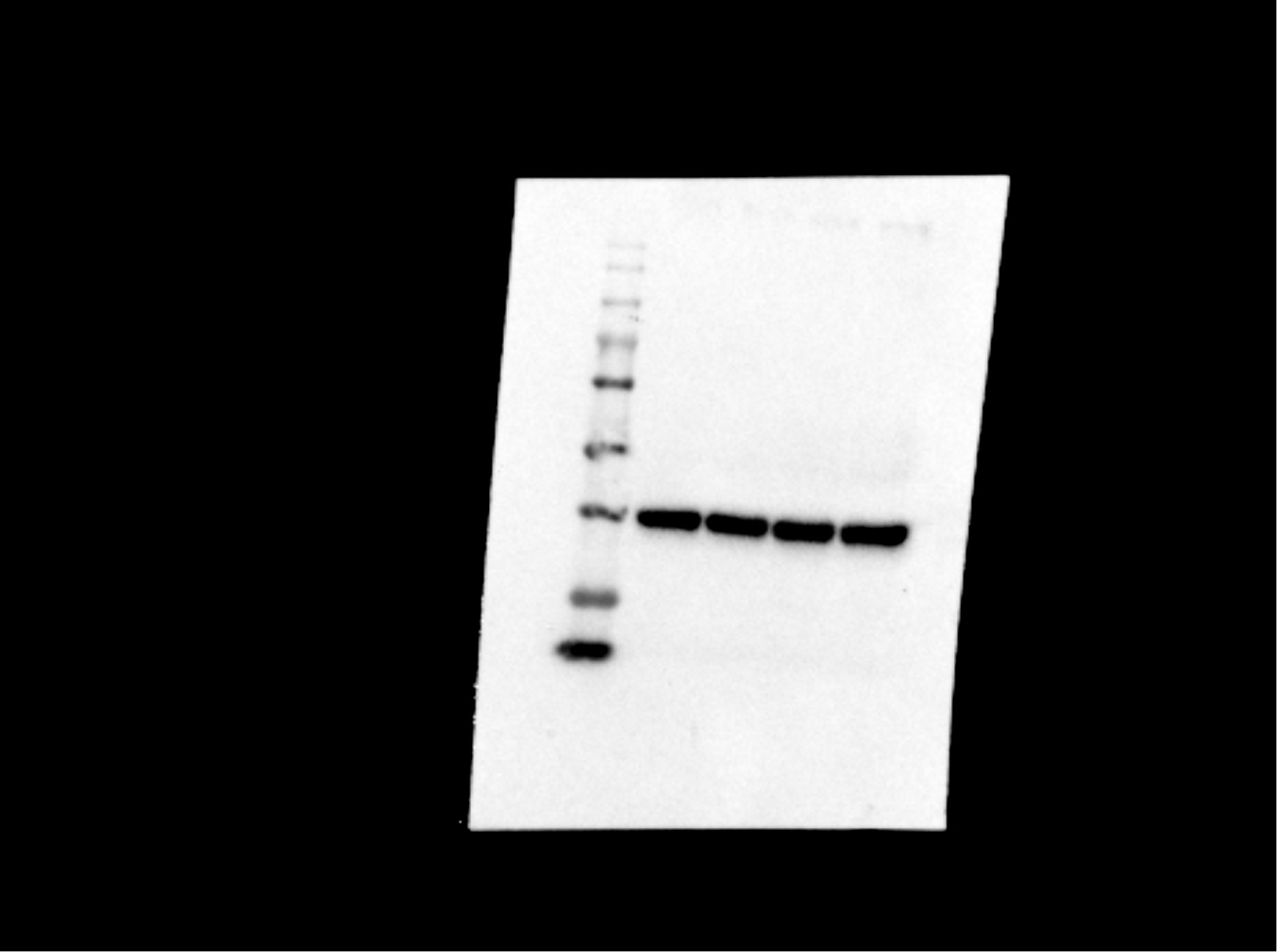


# WB bands of Figure 8D-1


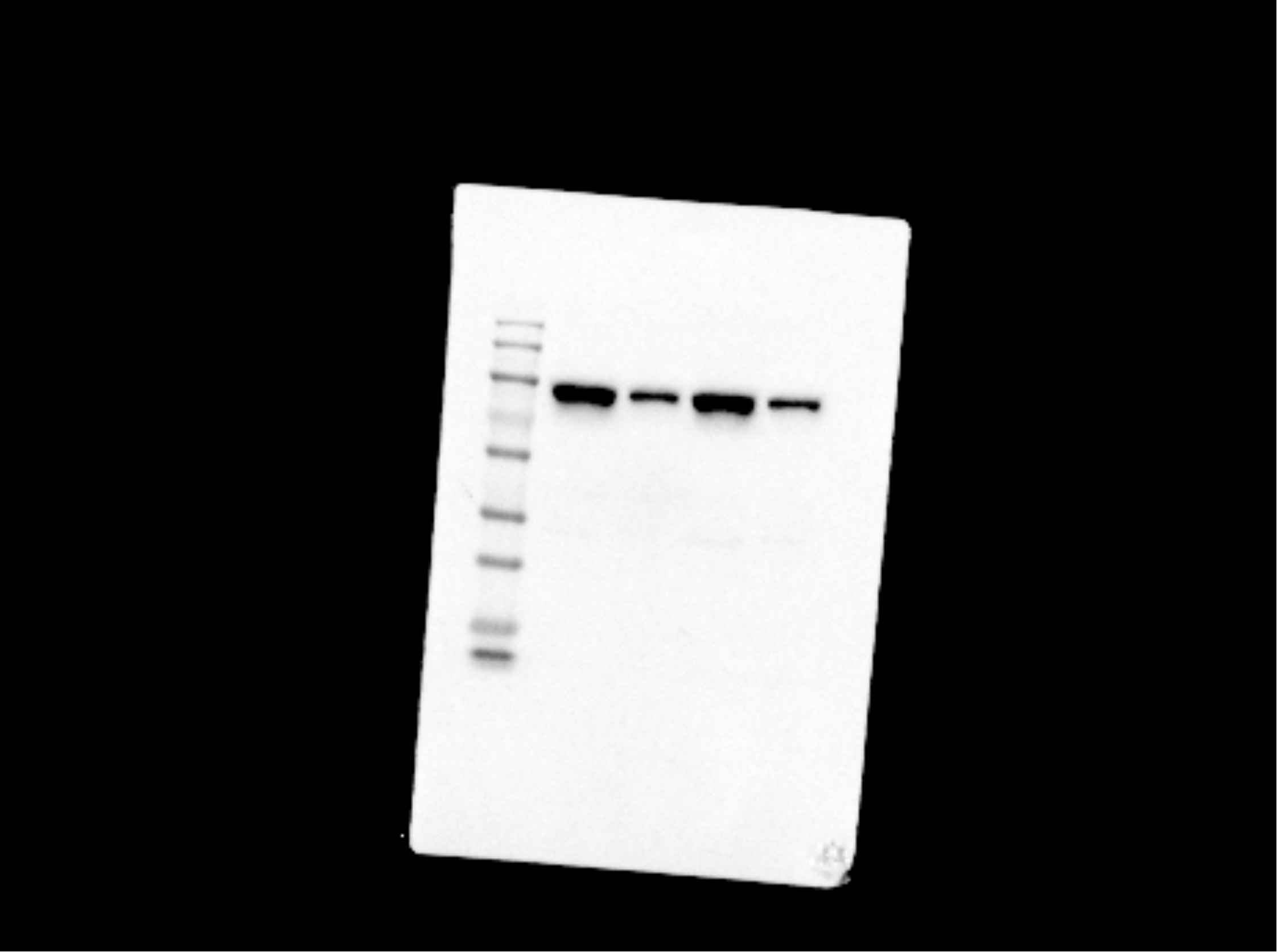


# WB bands of Figure 8D-2


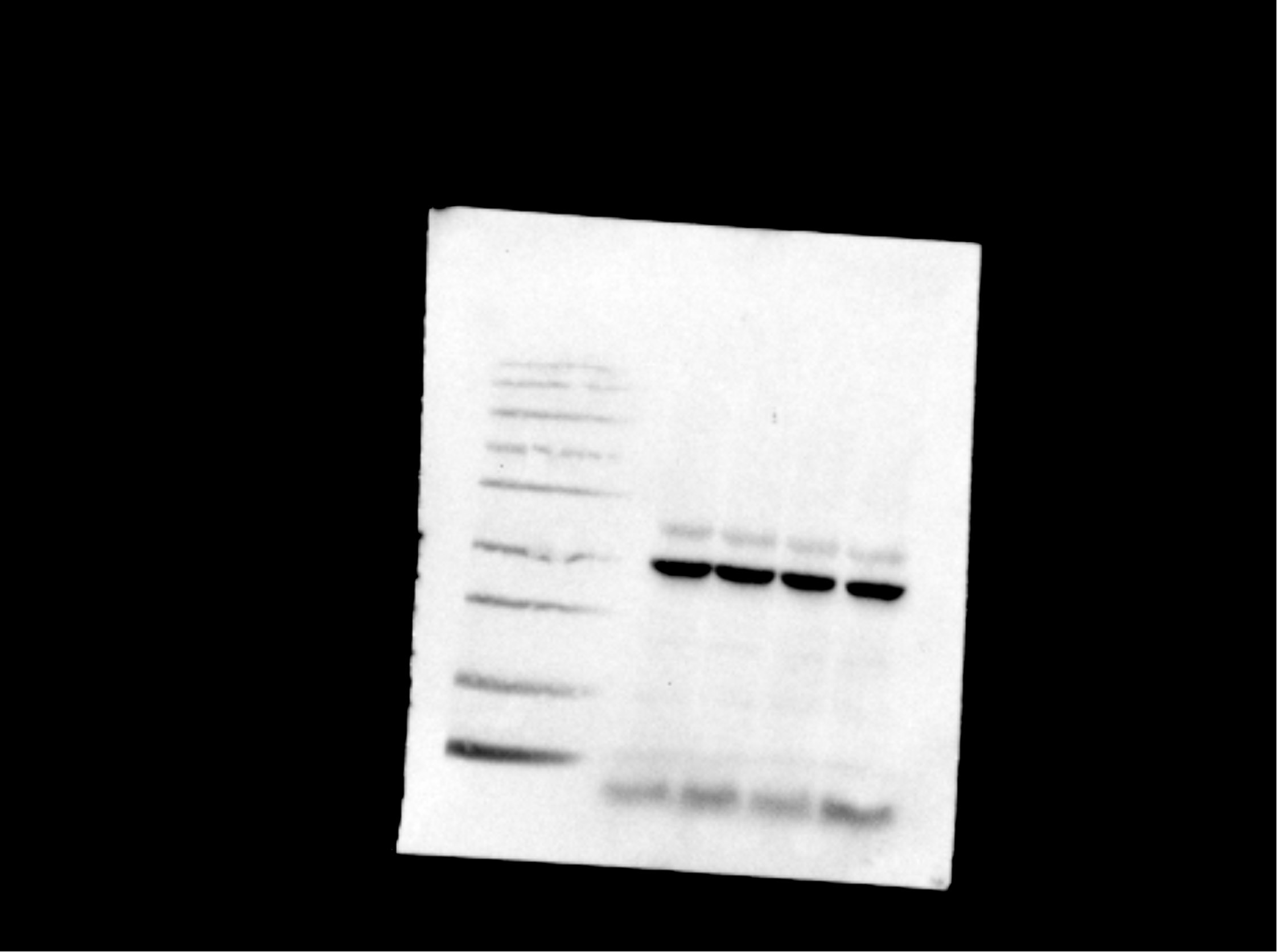


# WB bands of Figure 8F-1


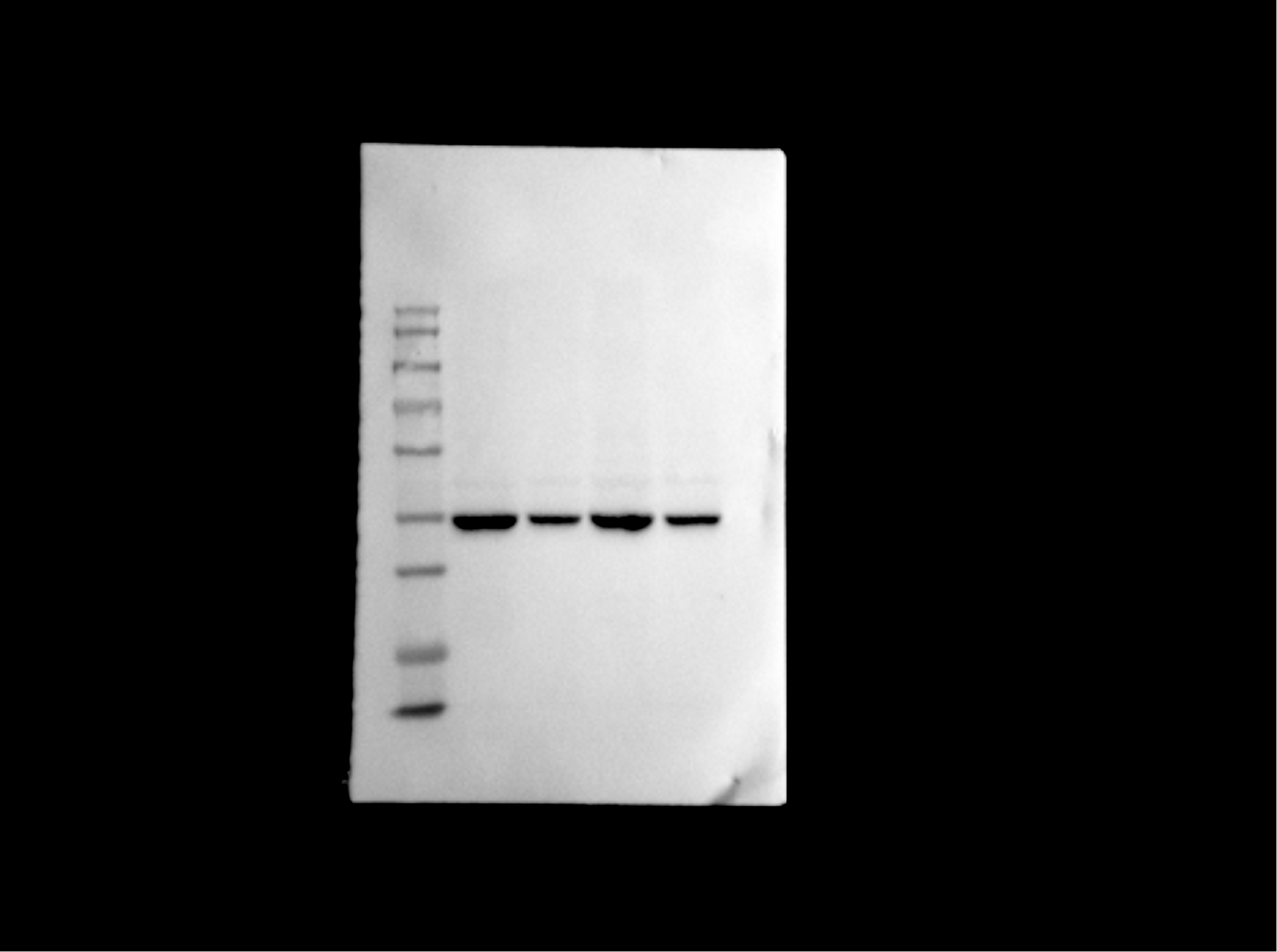


# WB bands of Figure 8F-2


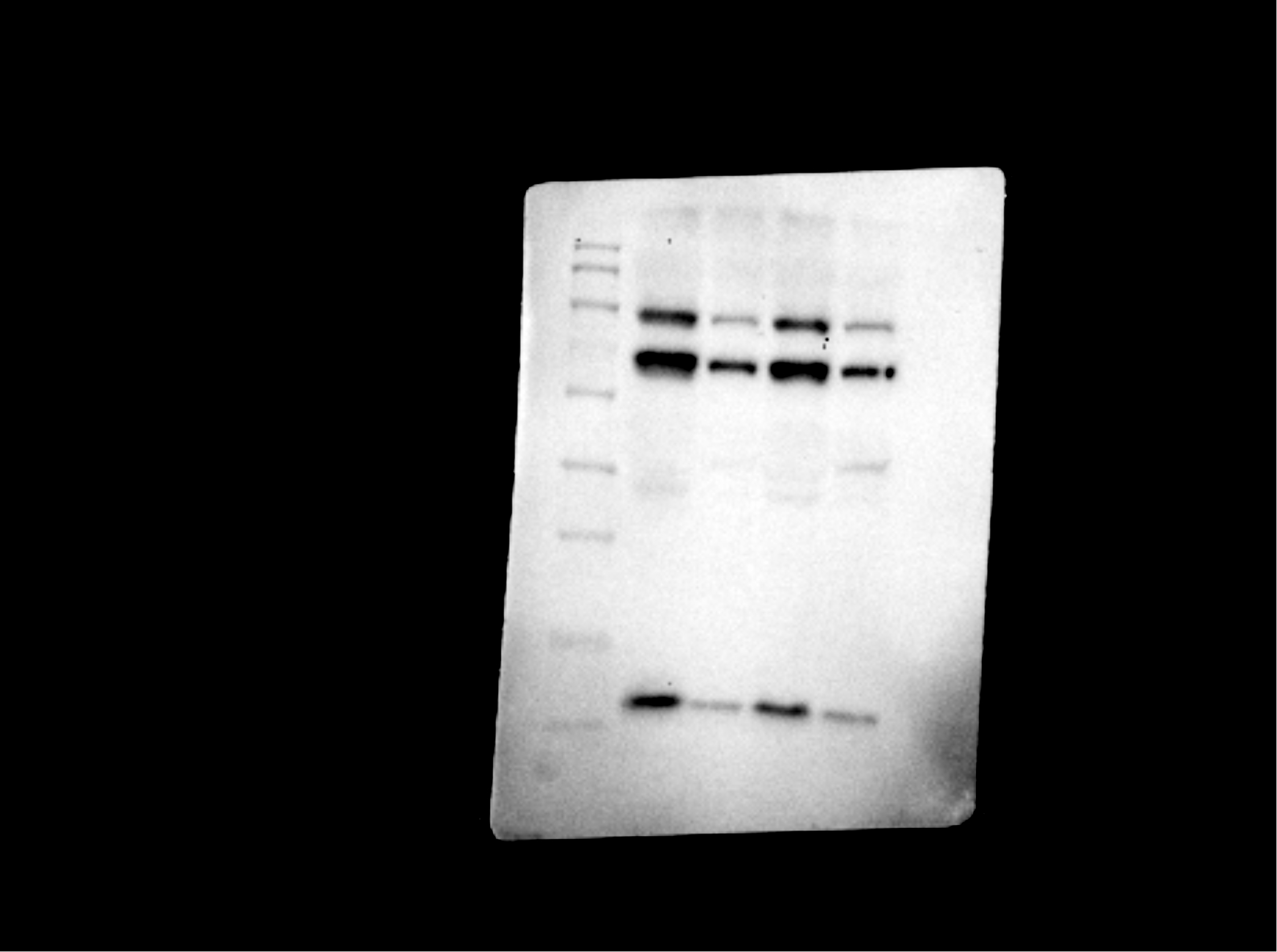


# WB bands of Figure 8F-3


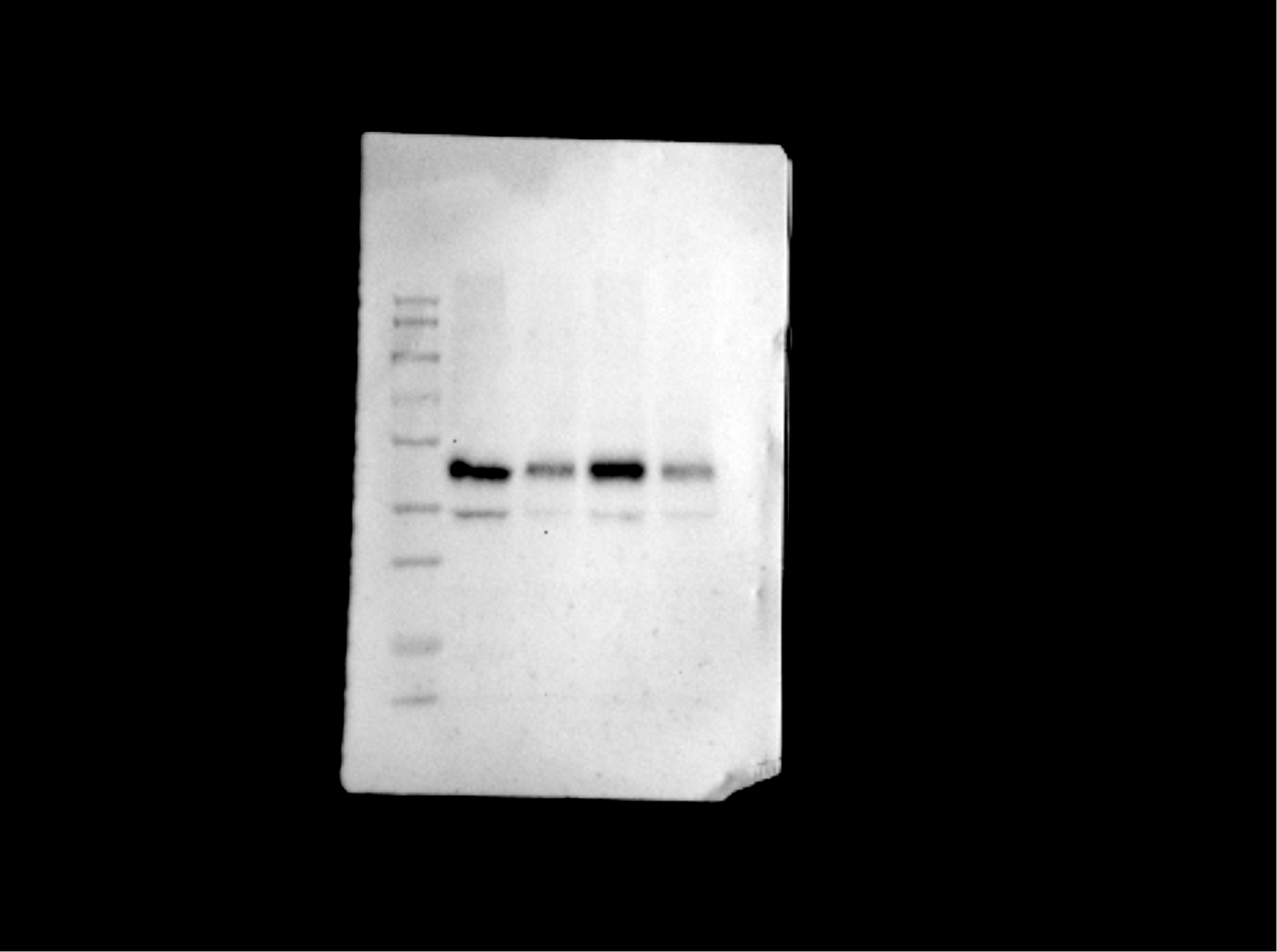


# WB bands of Figure 8F-4


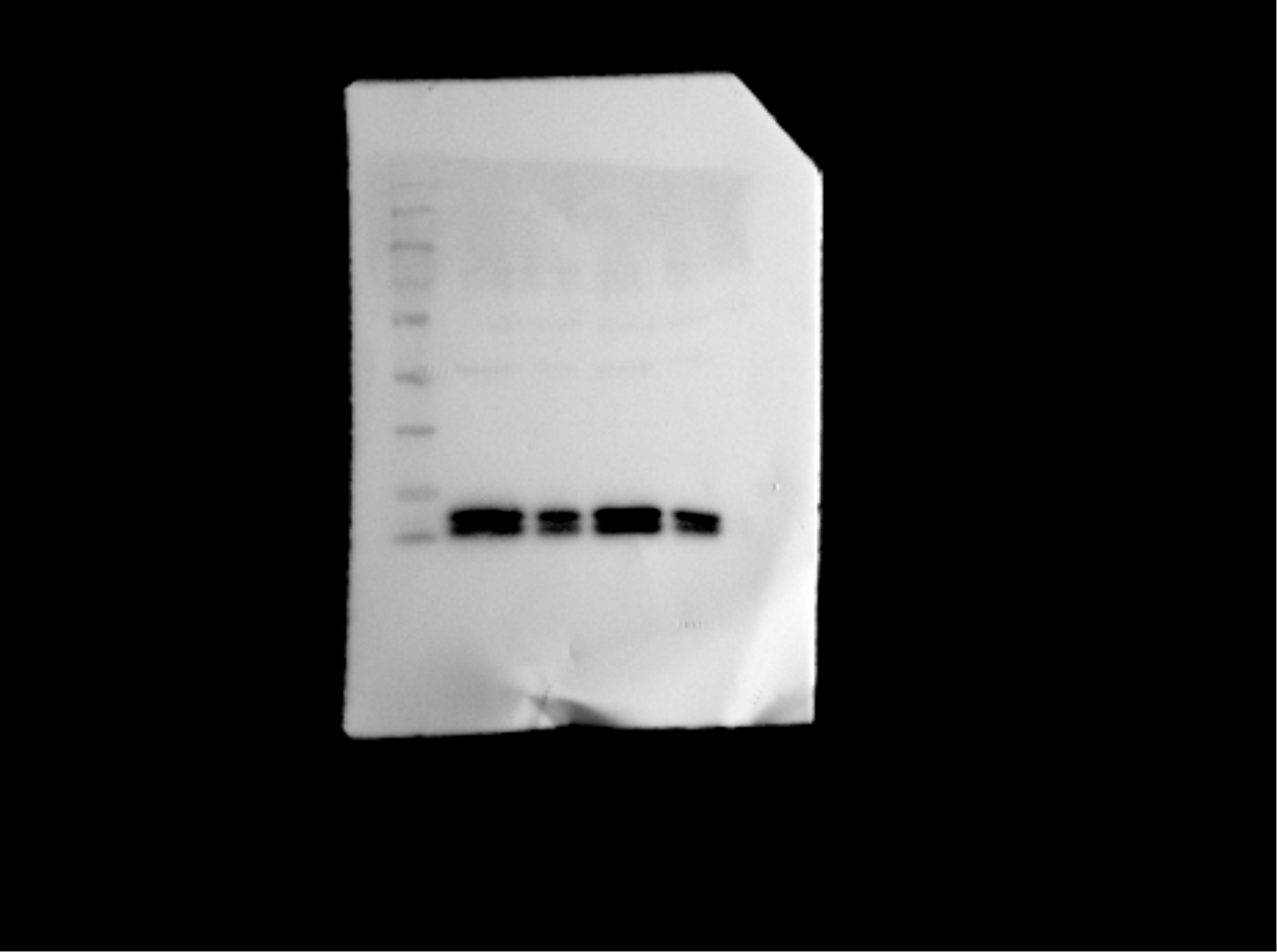


# WB bands of Figure 8F-5


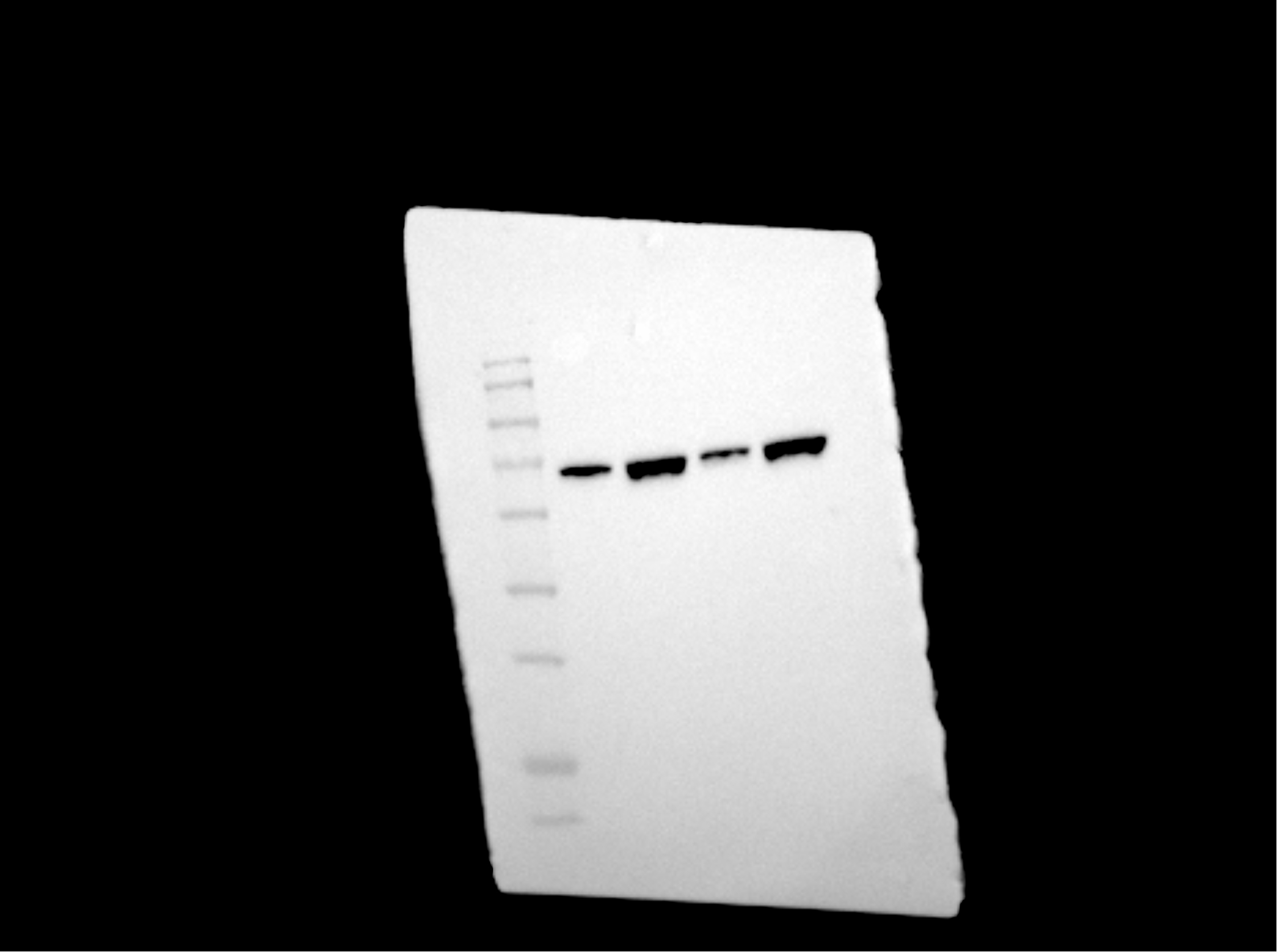


# WB bands of Figure 8F-6


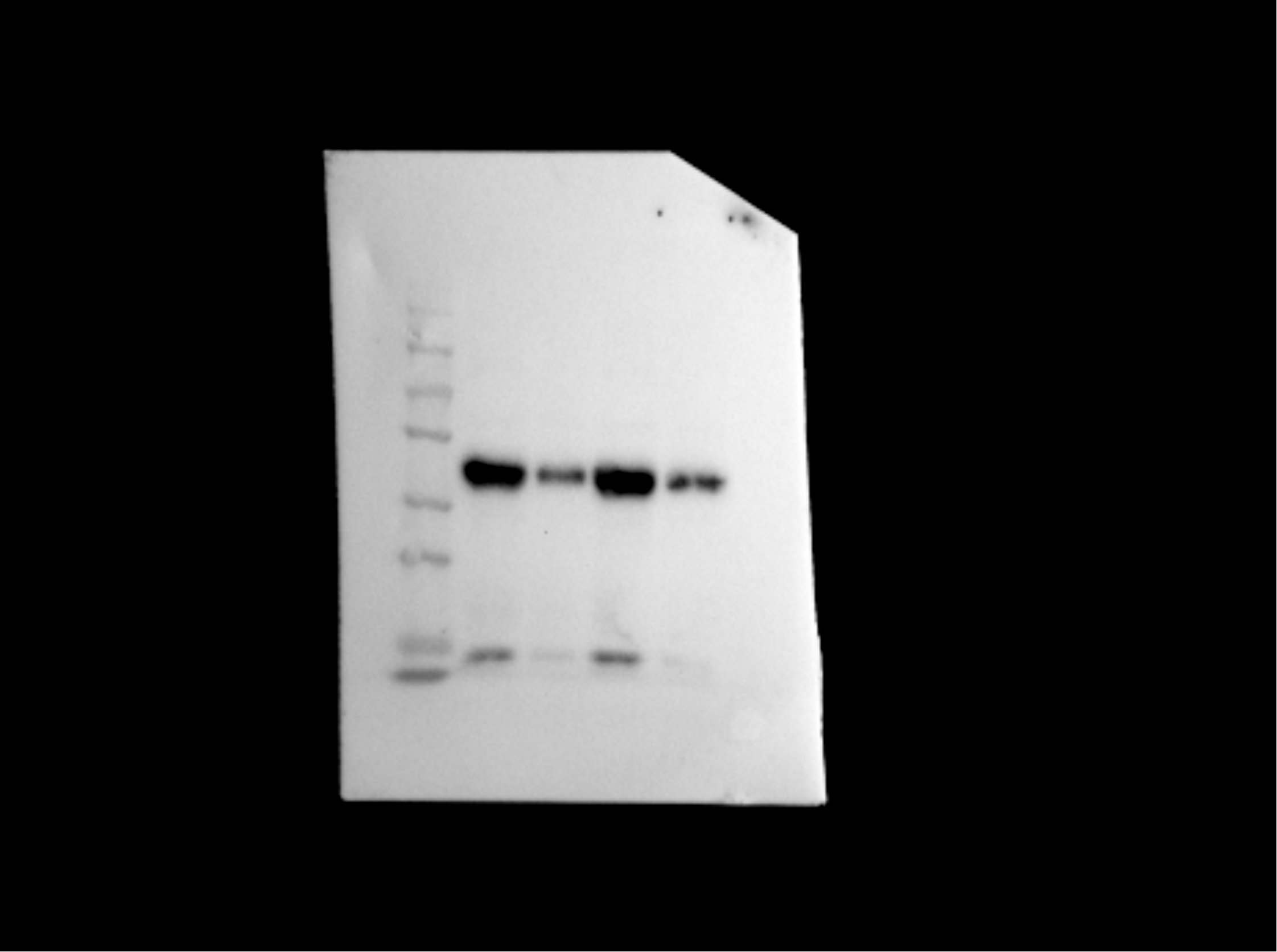


# WB bands of Figure 8F-7


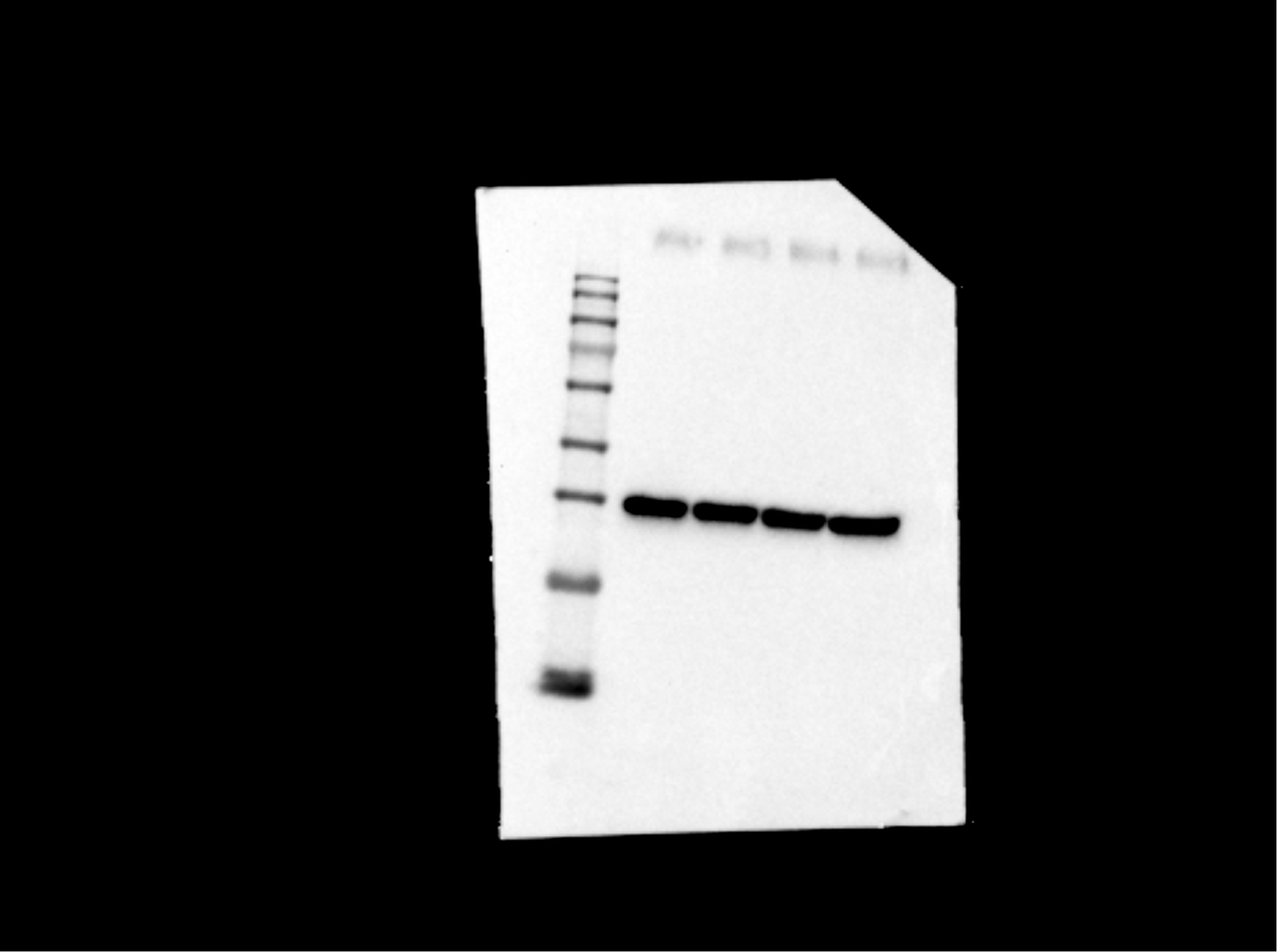


# WB bands of Figure S2D-1


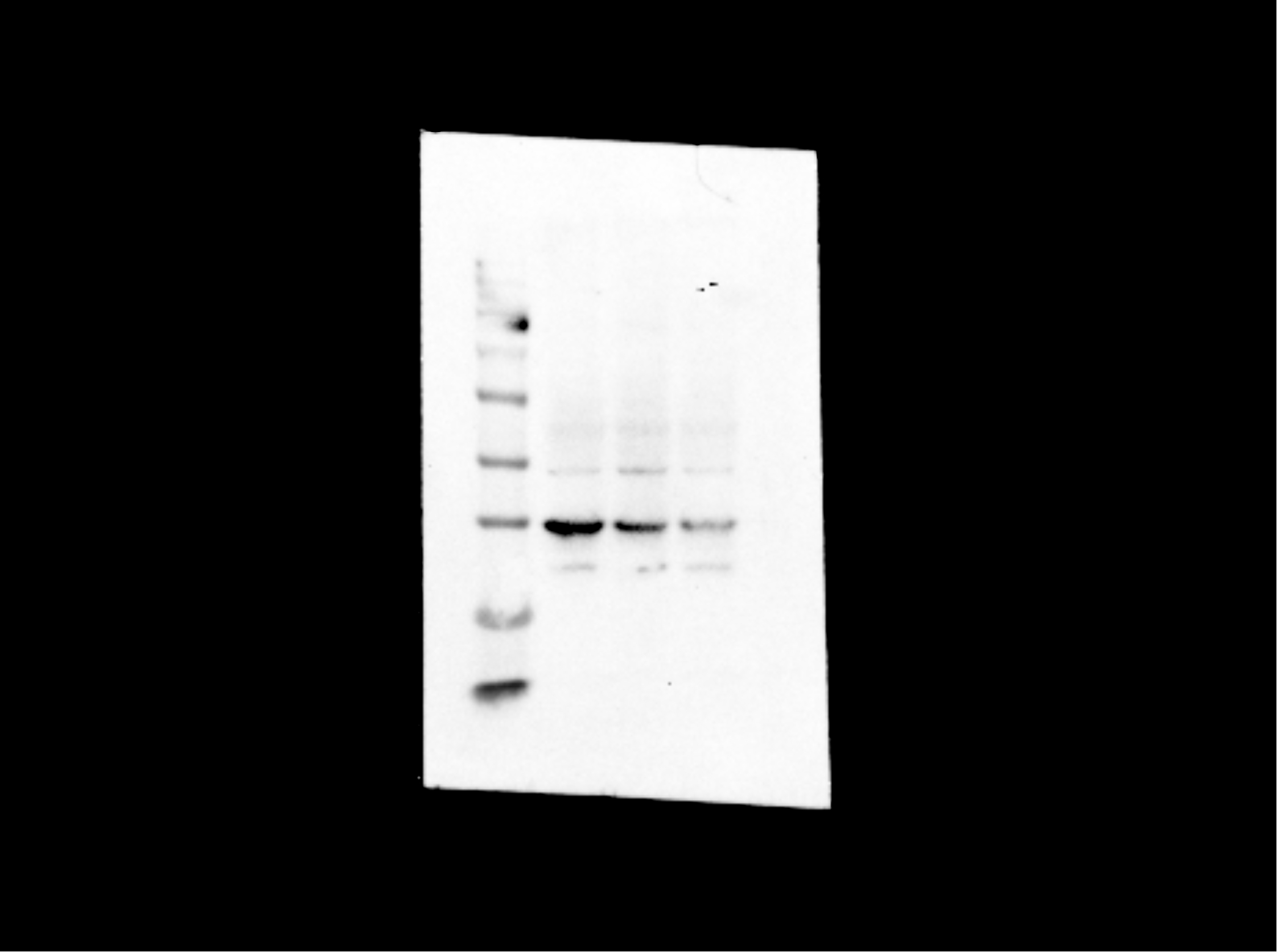


# WB bands of Figure S2D-2


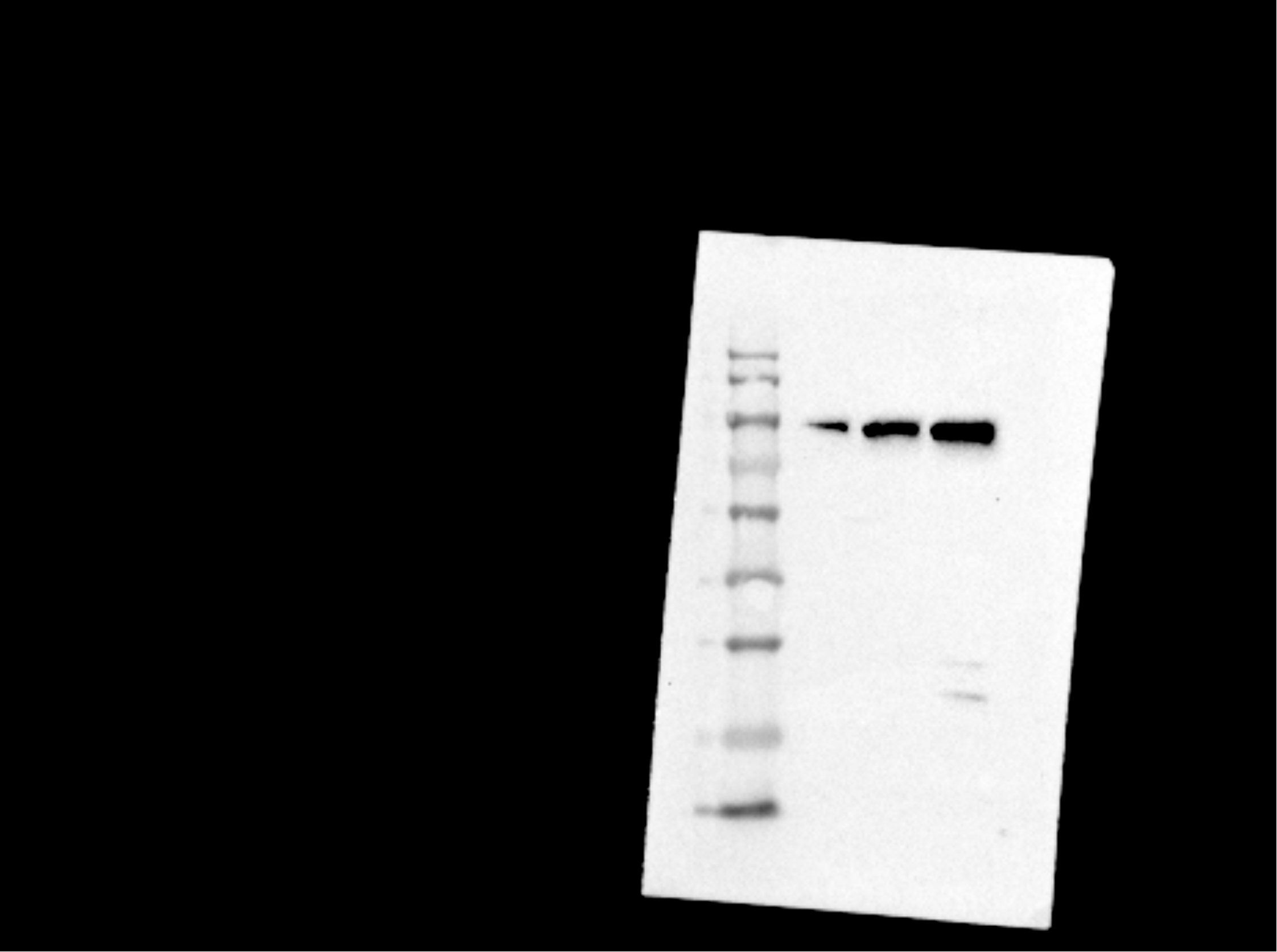


# WB bands of Figure S2D-3


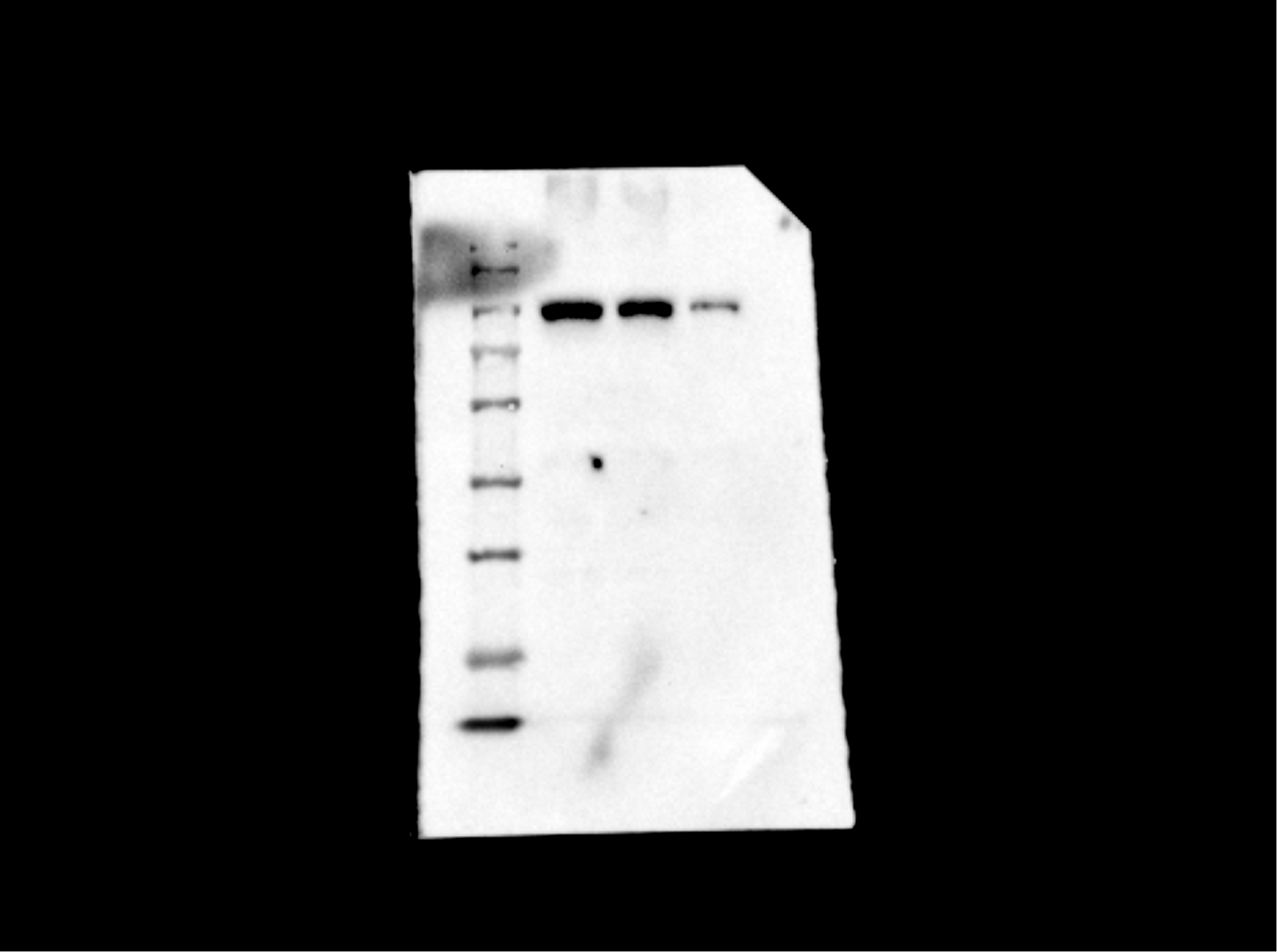


# WB bands of Figure S2D-4


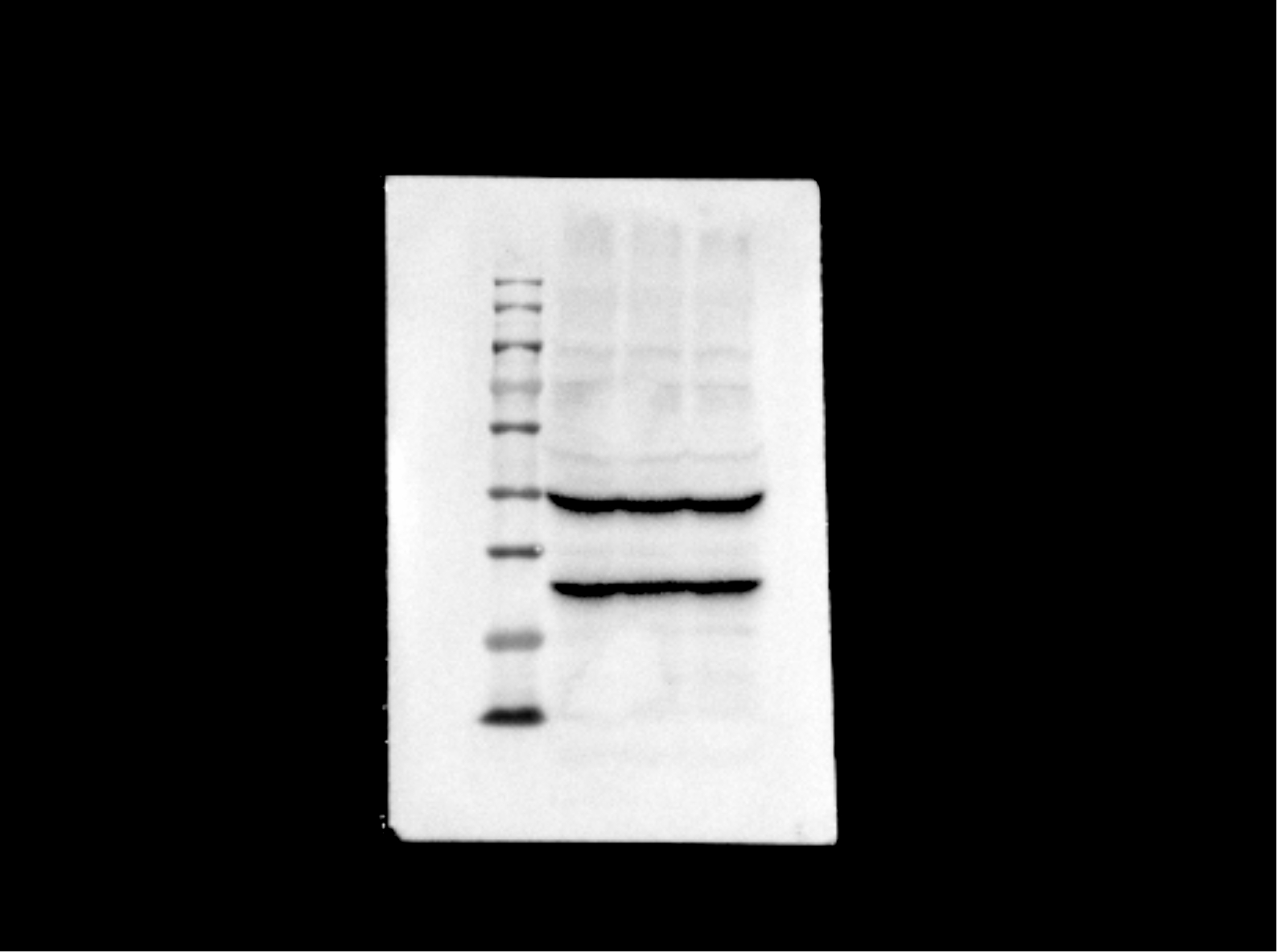


# WB bands of Figure S6A-1


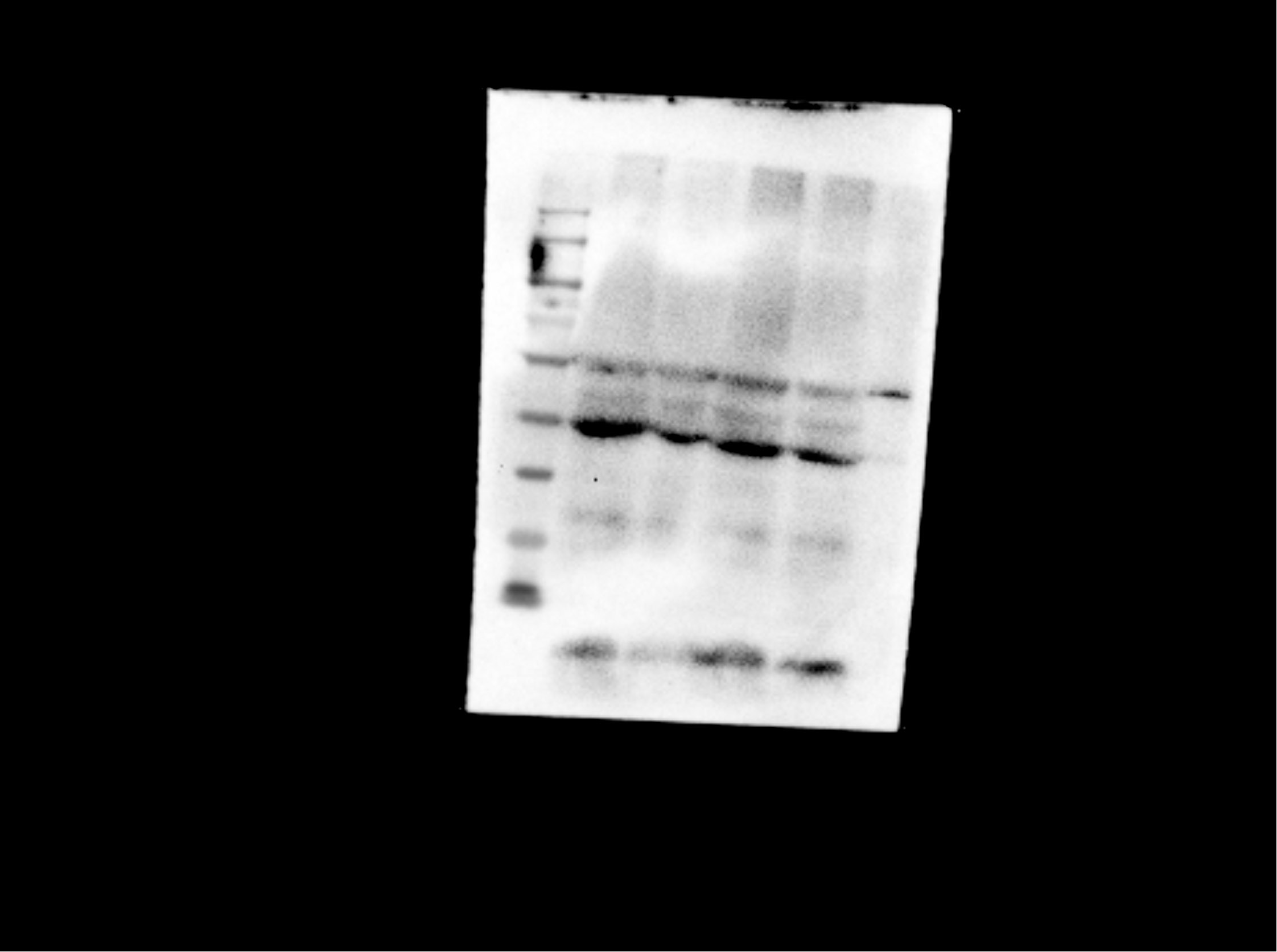


# WB bands of Figure S6A-2


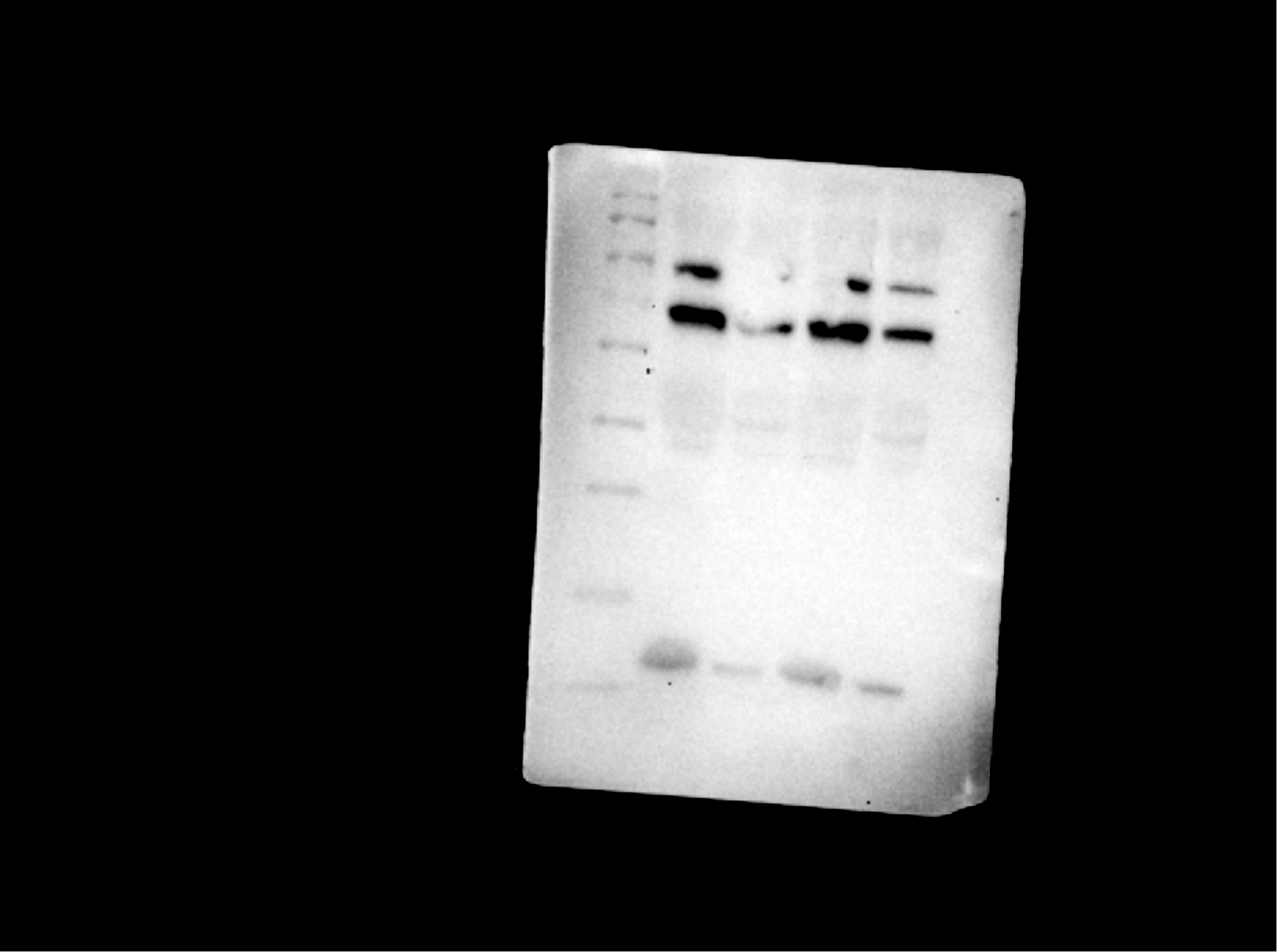


# WB bands of Figure S6A-3


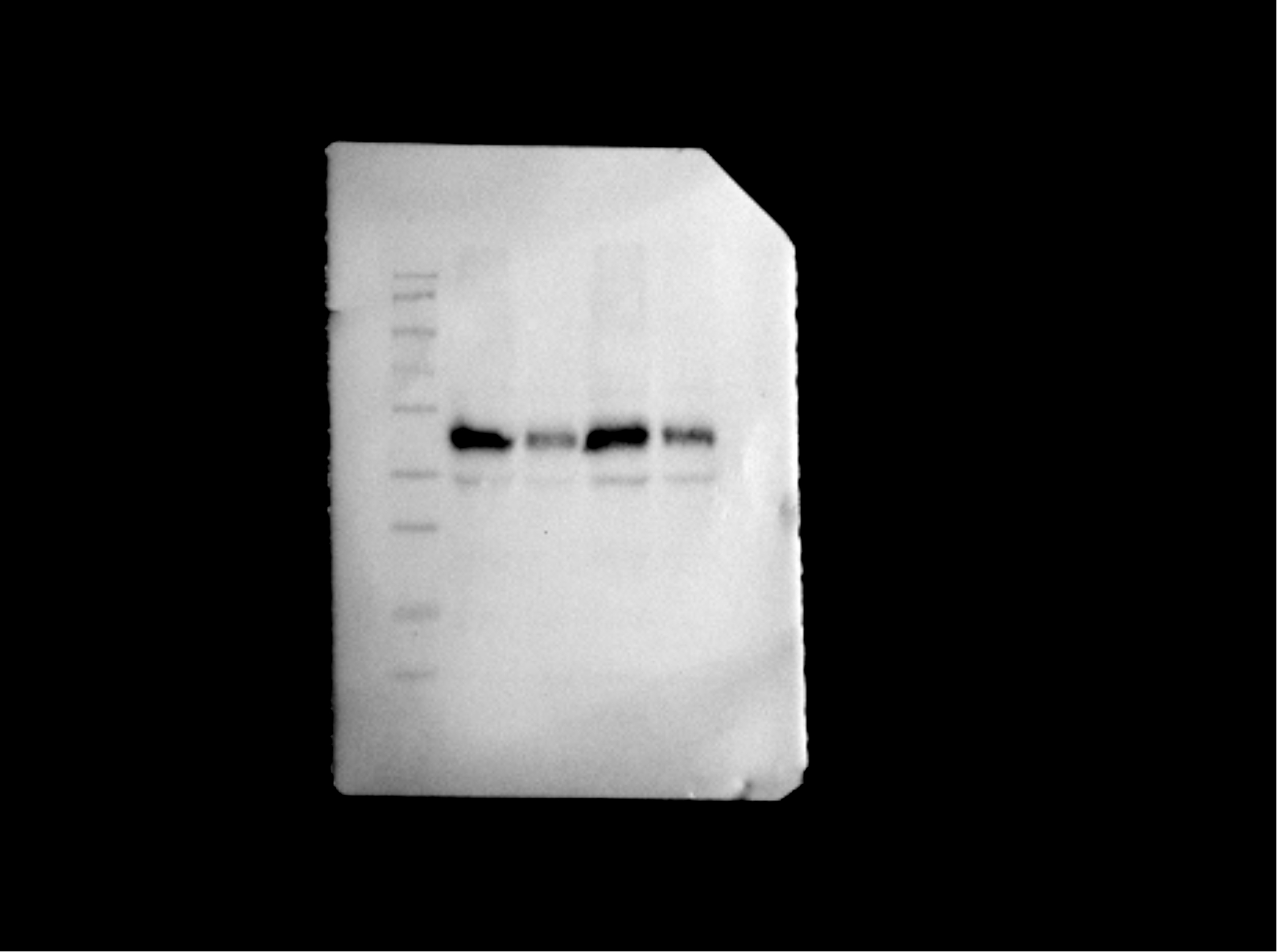


# WB bands of Figure S6A-4


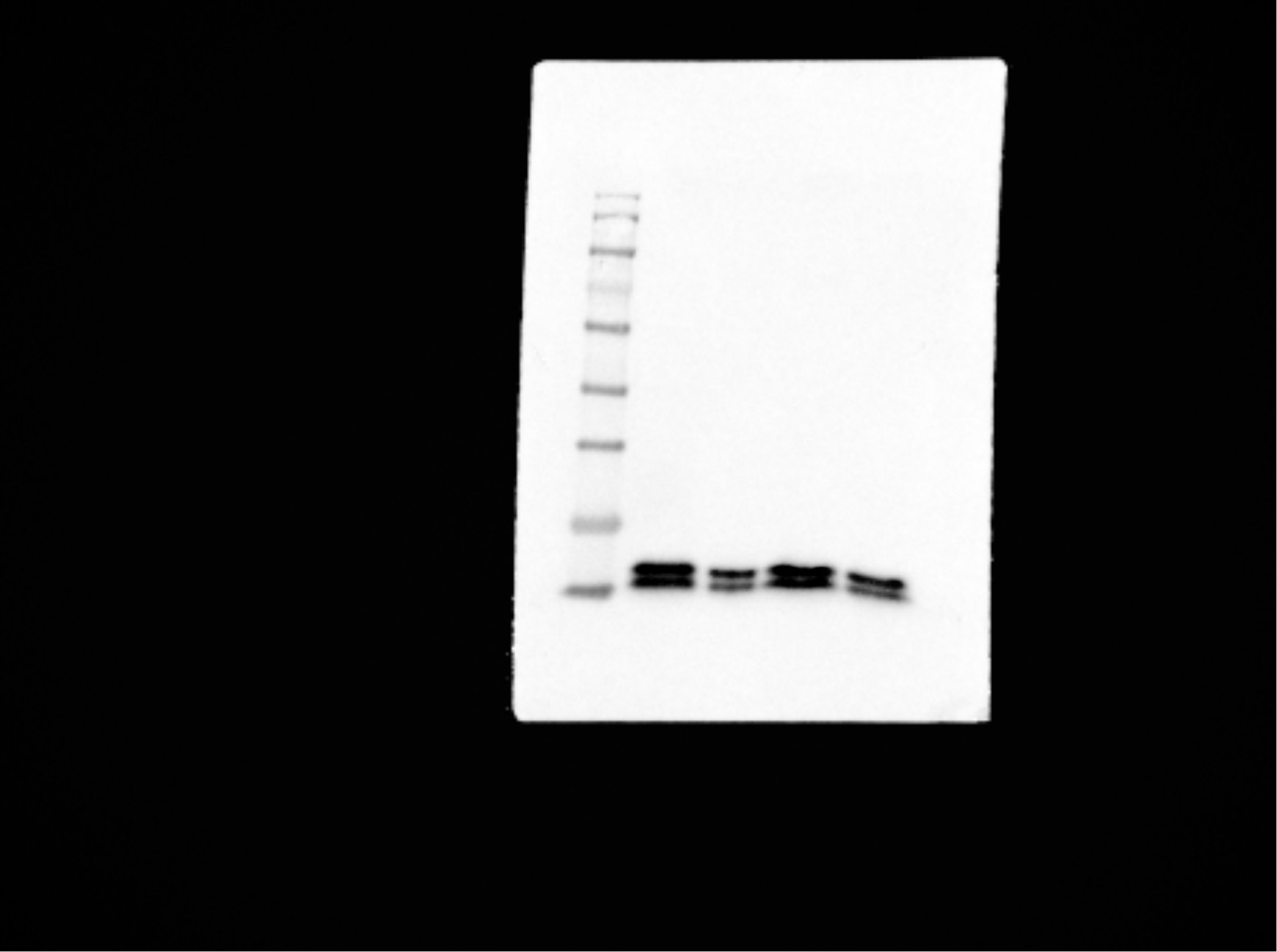


# WB bands of Figure S6A-5


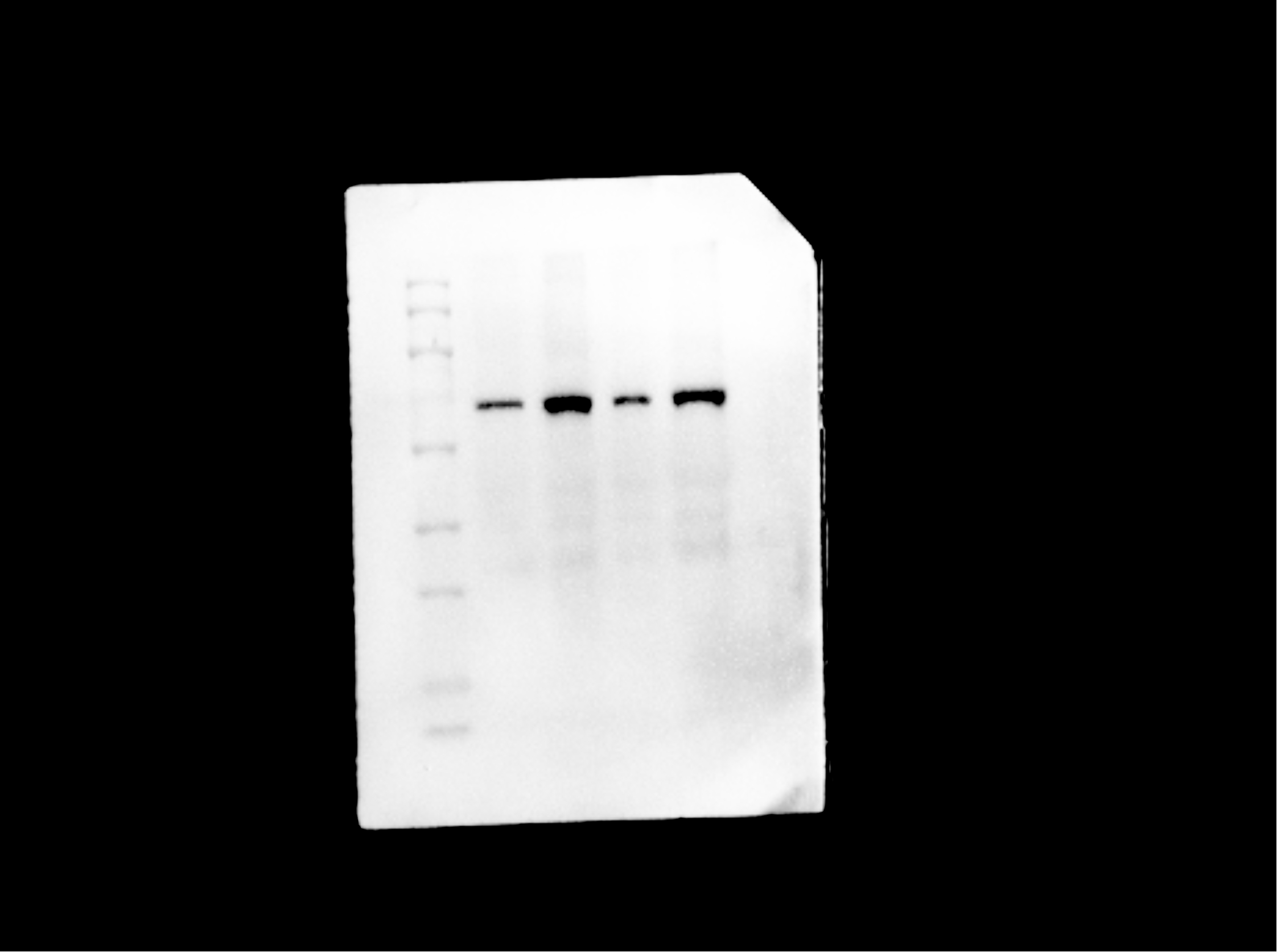


# WB bands of Figure S6A-6


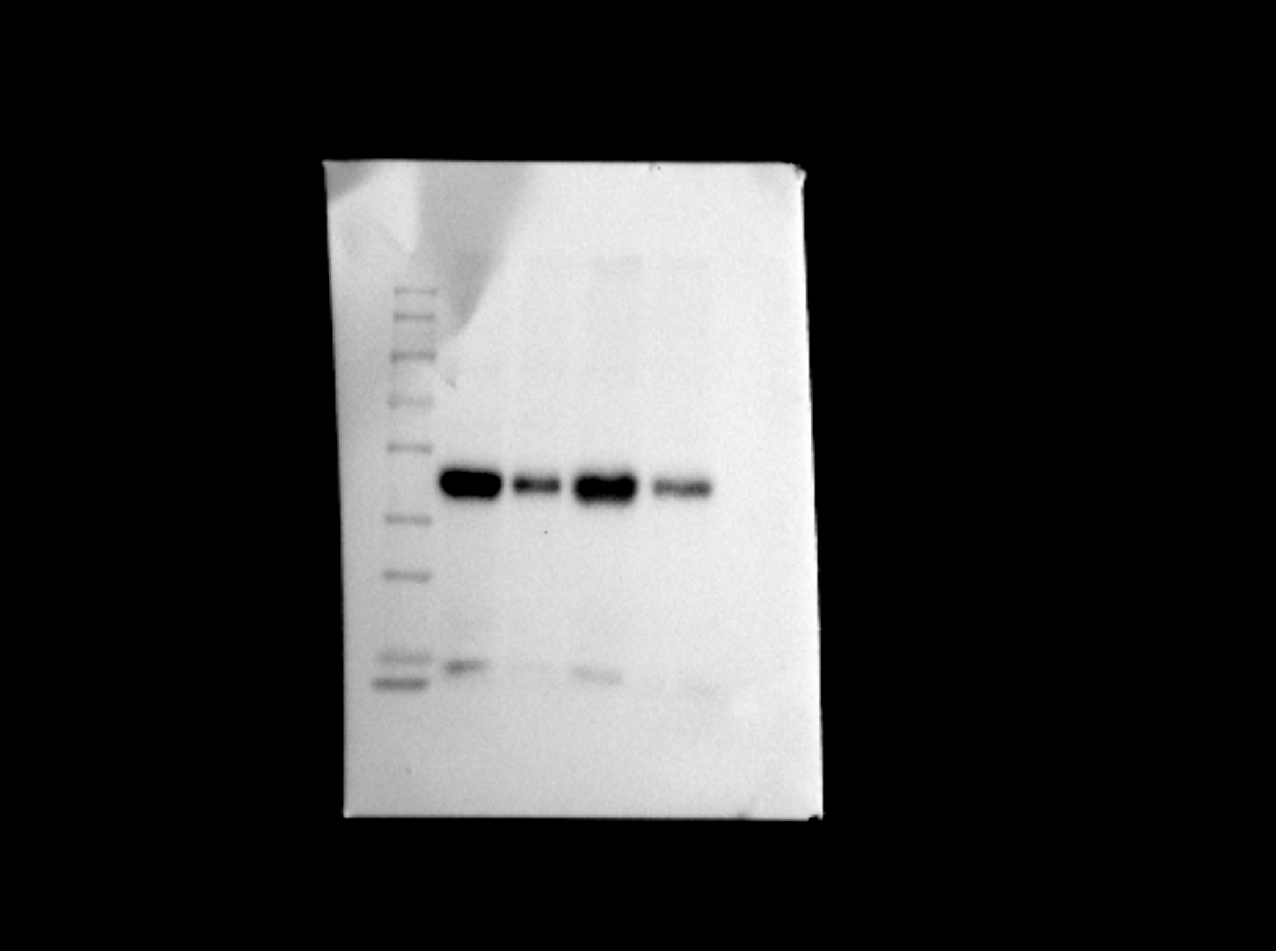


# WB bands of Figure S6A-7


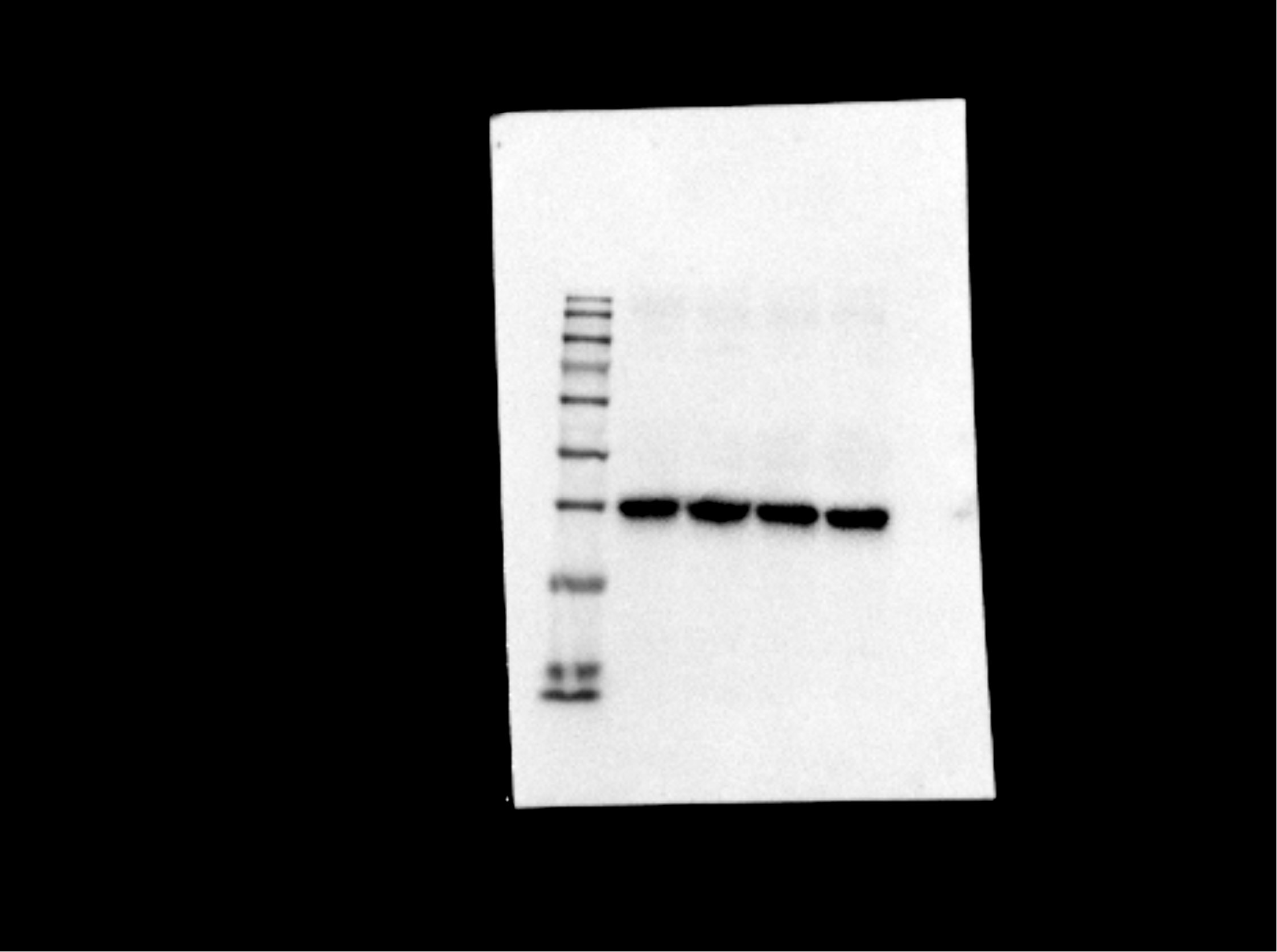

Supplement: Supplementary file 2 — Supporting Information [file ADVS-12-e15840-s001.docx]
